# Decomposition Analysis of Theoretical Raman Spectra for Efficient Interpretation of Experimental Spectra of Thin-Film Functional Materials

**DOI:** 10.3390/ijms262010237

**Published:** 2025-10-21

**Authors:** Marek Doskocz, Łukasz Laskowski, Jacek Kujawski, Agnieszka Karczmarska, Krzysztof Cpałka, Ewelina Lipiec, Magdalena Laskowska

**Affiliations:** 1 Institute of Nuclear Physics Polish Academy of Sciences, 31-342 Krakow, Poland; lukasz.laskowski@ifj.edu.pl (Ł.L.); agnieszka.karczmarska@ifj.edu.pl (A.K.); 2 Department of Organic Chemistry, Faculty of Pharmacy, Poznan University of Medical Sciences, Rokietnicka 3, 60-806 Poznań, Poland; jacekkuj@ump.edu.pl; 3 Department of Artificial Intelligence, Czestochowa University of Technology, Al. Armii Krajowej 36, 42-202 Częstochowa, Poland; krzysztof.cpalka@pcz.pl; 4 Faculty of Physics, Astronomy, and Applied Computer Science, M. Smoluchowski Institute of Physics, Jagiellonian University, Łojasiewicza 11, 30-348 Krakow, Poland; ewelina.lipiec@uj.edu.pl

**Keywords:** Raman spectroscopy, theoretical calculations, DFT, functional mesoporous silica materials, thin films

## Abstract

This study introduces a novel approach for analyzing theoretical Raman spectra, designed to facilitate spectral interpretation, particularly for complex systems such as functional mesoporous silica-based thin films. The proposed methodology relies on spectral decomposition supported by theoretical calculations, representing a step toward the development of autonomous research laboratories. The method assigns vibrational shifts to individual atoms within a molecular model and uses this information to generate partial spectra corresponding to specific atomic groupings. Unlike separate calculations for isolated components, this approach preserves the mutual interactions within the entire molecular structure, providing a more accurate representation of the vibrational environment. Decomposing the theoretical spectrum into contributions from atomic groups significantly simplifies the assignment of Raman bands to specific structural units, thereby enhancing the interpretative power of theoretical spectra and their correlation with experimental data. The method was demonstrated using real Raman spectroscopic data obtained from mesoporous SBA-15 silica thin films containing copper phosphonate groups. This work also highlights the critical role of molecular modeling and DFT calculations in Raman spectral analysis and outlines future perspectives for the use of artificial intelligence to automate and optimize the spectral interpretation process.

## 1. Introduction

There is a growing demand for the development of advanced materials with exceptional properties, and molecular engineering and nanotechnology methods make it possible to create precisely functionalized nanomaterials whose properties have the potential to revolutionize our lives [1,2,3,4,5]. Such materials are of immense importance, particularly in medicine, where targeted therapy plays a crucial role [6,7]. This is especially true in combating drug-resistant strains of bacteria or fungi [8,9]. In the case of functional nanomaterials, the synergistic interaction of the material’s individual components (such as the matrix and functional groups) is critical for achieving the desired effect, rather than the sheer quantity of active material. This is because the specific properties of such materials arise from their unique molecular configuration [10,11].

An example of a functional material of significant practical importance, but one that is extremely difficult to research, is functionalized porous silica. Functional materials based on mesoporous silicas can exhibit either disordered or ordered spatial structures, with a wide variety of pore shapes and diameters ranging from 2 to 50 nm (e.g., SBA-15, TUD-1, HMM-33, and FSM-16) [12,13,14,15,16,17,18]. The spatial structure of these matrices allows single atoms, molecules, or nanoparticles to be deposited within them, either permanently or temporarily [19,20,21]. Functional materials based on porous silicas are widely used in biomedicine [22] due to their high surface area, porosity, and biocompatibility [23], especially as drug delivery systems [24], templates in tissue engineering [25], or wound healing dressings and biosensors [26]. In environmental applications [27,28,29], they are used for purification and oxidation of volatile compounds, as catalysts for a variety of chemical reactions [30], including those used in green chemistry [31] or CO_2_ removal [32], and as sensors [33], electrodes [34,35], absorbers of chemical compounds, and sorbents [36]. Additionally, they find use in high-tech applications such as nonlinear optics [37], quantum dots [38], quantum computers [39,40,41], and memristors [42]. In the PubMed database, the number of publications from the last 10 years on SBA-15 silica alone is around 3000, which indicates its significant application potential.

Given this, the design and synthesis of new functional materials based on porous silica hold significant promise. To achieve the desired properties, the material’s molecular structure must be tailored to support them, often by ensuring the proper distribution of functional groups. However, predicting all physical phenomena within the material is challenging, and the arrangement of these groups frequently leads to specific molecular configurations that influence the material’s properties. Therefore, it is crucial to determine the exact molecular structure of the synthesized material and confirm that it aligns with the original design assumptions.

A key research challenge is verifying whether the actual molecular configuration of the nanomaterial matches the intended one and is linked to its properties. Experimental verification is not straightforward. Vibrational spectroscopy, combined with numerical simulations, provides an efficient approach for testing such materials. Raman spectroscopy, in particular, is widely used for its ability to offer molecular specificity with minimal sample preparation. It enables the identification of vibrational modes corresponding to structural features and interactions. As it offers the possibility of qualitative and quantitative analysis, it is also used in diagnostics and metabolomics [43]. However, analyzing the molecular structure of functional nanomaterials is complex and requires substantial expertise, especially for thin-film materials, where small sample thickness often leads to lower-quality spectra and more difficult interpretation [44,45].

When conducting research on functional materials, especially those that contain single molecules as functional groups, the main challenge is the correct interpretation of experimental data. Often, the spectra of similar materials differ only subtly, and an unambiguous assessment of the differences is problematic. Spectroscopic methods for studying functional nanomaterials can be greatly enhanced by incorporating theoretical calculations and artificial intelligence (AI) into the methodology [19]. Such supporting methods not only improve the quality of analysis but also significantly automate the process of interpreting measurement data. The concept of an artificial and autonomous scientist, responsible for solving various research challenges, is expected to be widely adopted in laboratories in the near future [46]. This tremendous progress in the development of AI makes it extremely important to systematically present the possibilities that artificial intelligence gives us in analytical methods such as Raman spectroscopy, among others.

**Figure 1:**
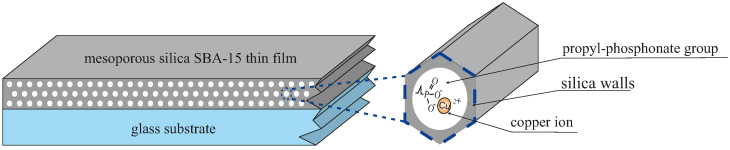
The overview diagram of the materials investigated: SBA-15 silica thin film containing propyl copper phosphonate units.

In this study, we present a novel approach for extracting information from Raman spectroscopy data, supported by theoretical calculations and spectral decomposition. This methodology relies on cutting-edge IT solutions and represents a first step towards the development of autonomous laboratories, where synthesis and measurement tasks could eventually be performed by robots [47,48]. This approach has been demonstrated using real Raman spectroscopy experimental data obtained from thin-film functional materials: mesoporous silica SBA-15 containing copper phosphonate functional groups distributed homogeneously within the pores, as illustrated in Figure 1. The sample will be referred to as SBA-POO_2_Cu+Si(CH_3_)_3_. The thin-film form of the sample additionally complicates characterization due to the significant contribution of the substrate to the spectrum. Therefore, we decided to prepare a reference sample with no copper ions (referred to as SBA-PO(OH)_2_+Si(CH_3_)_3_) and support the analysis of experimental spectra with numerical simulations (see Section 3).

## 2. Results and Discussion

An important aspect that significantly affects the analysis of Raman spectra is the proper preprocessing of the spectral data. This step involves several correction procedures, such as baseline subtraction, signal smoothing, and cosmic ray removal. Proper execution of these operations is essential for obtaining reliable and precise spectral information, which directly affects the quality of Raman spectra interpretation. Inaccuracies in preprocessing can lead to misinterpretation of vibrational modes or incorrect peak assignments, especially for complex molecular systems. This is the case with the functionalized silica composites discussed here. In addition, special attention must be paid to the calibration process and the selection of appropriate parameters during spectral acquisition, such as laser wavelength, the numerical aperture of the objective, and spectrometer configuration. The decision on settings is usually made by analyzing spectral quality at varying laser powers, exposure times, and scan numbers. Another important factor is the selection of the appropriate spectral region for analysis, which should be oriented to the nature of the sample and the scientific question under consideration. The choice of parameters and adjustments applied during spectral preparation can significantly affect the final outcome of computational and experimental comparisons. Given the multidimensional nature of spectral data and the number of variables involved, artificial intelligence offers promising tools to automate and optimize these preprocessing steps. AI-based approaches can learn from large datasets to identify optimal correction strategies, perform adaptive baseline removal, and select appropriate methods for smoothing spectra. However, the development and implementation of such AI-based methodologies will be the subject of our next publication. Here, we will focus on supporting the interpretation of experimental data with numerical calculations and on the spectral decomposition of such data.

Currently, many chemical and physical studies are supported by routine calculations of molecular structures and parameters such as interaction energy, vibrations, and charge distribution. Quantum chemistry methods enable the visualization of vibrations and the precise assignment of bands in spectroscopic spectra. These theoretical calculations can be performed using various software programs, e.g., Gaussian [49] and SCIGRESS [50], and with different levels of accuracy. Our calculations and considerations were based on the Gaussian software (Gaussian 16 Revision C.01) due to its widespread use; however, the presented methods can be adapted to results obtained from other computational programs. The more advanced the methods and the larger the base sets, the greater the computational power required. It is impossible to select a single universal theoretical method that provides results very close to the experimental data while maintaining a low computational cost [51]. Artificial intelligence is also entering this field, providing new tools for molecular simulations and predictions [52,53,54,55,56,57]. Theoretical calculations can be performed for various systems, ranging from single molecules to molecules with solvent interactions, multimolecular systems (dimers and trimers), and even crystalline structures [58]. Even a small molecule can exist in multiple conformations, and finding a single energy minimum, even a global one, does not guarantee that it is the only existing structure. By applying the Boltzmann distribution [59] and considering all potential conformations, we can approximate the conformational population. However, determining whether the identified structure possesses biological activity remains challenging [60]. The calculated conformation with the highest population is expected to contribute the most to the simulated theoretical spectra [61]. Additionally, errors arise from the limitations of computational methods and the size of the base sets. Therefore, theoretical calculations serve as a valuable tool for supporting spectral interpretation but do not replace experimental analysis.

However, all calculations must be preceded by the preparation of an appropriate model. It is important to think carefully about the components of the model. On the one hand, it should contain key components responsible for the bands observed in the experiment. On the other hand, it should not contain too many atoms. For example, the model of silica can be prepared using common knowledge that the predominant structures typically consist of silicon frameworks with silicon atoms in Q3 (H-O-Si(OSi-)_3_) and Q4 (Si(OSi-)_4_) configurations. However, if we analyze the structures of different types of silica more closely, we will notice certain differences that also affect the observed Raman spectra.

It often turns out, as in the case of SBA-15, that a model that accurately reflects reality cannot be built from a few atoms. According to the literature, characteristic microporosity of SBA-15 is observed due to the silica walls built of silica rings containing most likely five to six silicon atoms [19,44,45,62,63,64]. Some researchers have simplified the models of silica substitute Si-H fragments instead of Si-O-H [24,44,45,62,65,66]. Although most silica synthesis methods do not allow for the formation of such a system in practice, this simplification significantly reduces computational costs. However, to correctly interpret the results of the calculations, the differences in the spectra obtained as a result of this simplification must be known.

The same situation can be observed when the computational methods and basis sets are considered. To analyze the differences between the results of calculations with the use of various methods and basis sets, as well as the simplification, we performed calculations for two model systems:

- SBA-PO(OH)_2_+3(Si(CH_3_)_3_) consists of four silica rings forming both planar and three-dimensional structures, three TMS groups (O-Si(CH_3_)_3_) substituting hydroxyl groups, one hydroxyl group, and one -CH_2_CH_2_CH_2_PO(OH)_2_ group, and 17 Si-H hydrogen atoms. This model consists of 124 atoms.
- SBAH-PO(OH)_2_+3(Si(CH_3_)_3_) is similar to SBA-PO(OH)_2_+3(Si(CH_3_)_3_) but lacks Si-H groups, replacing them with Si-OH. This model consists of 107 atoms.

**Figure 2:**
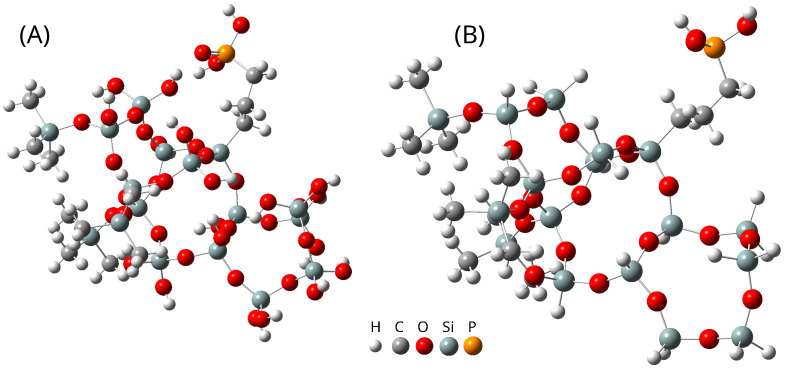
(**A**) The stable structures of SBA-PO(OH)_2_+3(Si(CH_3_)_3_). (**B**) The stable structures of SBAH-PO(OH)_2_+3(Si(CH_3_)_3_). The structures were optimized using the B3LYP functional with the def2-TZVP basis set.

The model systems are illustrated in Figure 2.

**Figure 3:**
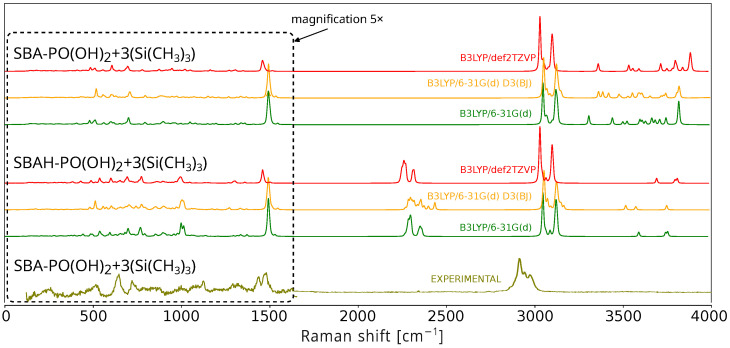
Spectra of the SBAH-PO(OH)_2_+3(Si(CH_3_)_3_) and SBA-PO(OH)_2_+3(Si(CH_3_)_3_) structures obtained using three computational methods: B3LYP/6-31G(d) (green spectra), B3LYP-D3(BJ)/ 6-31G(d) (yellow spectra), and B3LYP/def2TZVP (red spectra). The results are compared with the experimental spectrum of SBA-PO(OH)_2_+Si(CH_3_)_3_ (olive spectra).

To evaluate the vibrational properties of the SBA-PO(OH)_2_+3(Si(CH_3_)_3_) (124 atoms) and SBAH-PO(OH)_2_+3(Si(CH_3_)_3_) (107 atoms) systems, Raman spectra were simulated using three different methods: B3LYP/6-31G(d), B3LYP/6-31G(d) with D3(BJ) dispersion correction, and B3LYP/def2TZVP. Figure 3 presents the computed Raman spectra for both models along with the experimental spectrum of SBA-PO(OH)_2_+Si(CH_3_)_3_. The spectra in the range below 1500 cm^−1^ are magnified fivefold to enhance clarity. As expected, the duration of the geometry optimization is highly dependent on the initial structural configuration. Frequency analyses revealed that, in comparison to B3LYP/6-31G(d), B3LYP-D3(BJ)/6-31G(d) required approximately 25% more computational resources, whereas B3LYP/def2TZVP exhibited a more than tenfold increase in computational demand. Moreover, the B3LYP/def2TZVP method, despite significantly increasing computational costs, did not result in a commensurate improvement in spectral agreement. An obvious effect of the substitution of Si-OH groups with Si-H groups in the model system was also observed. This substitution leads to a noticeable reduction in the number of vibrational modes in the region above 3000 cm^−1^, which is consistent with the removal of O-H stretching vibrations. In contrast, the theoretical spectra for the SBAH-PO(OH)_2_+3(Si(CH_3_)_3_) model contain additional bands in the 2300–2400 cm^−1^ range from Si-H bond vibrations. These bands, for obvious reasons, are not observed in the experimental spectrum because this model is only a simplification and we do not have to deal with such formations in the samples of the materials studied. However, the reduced model leads to an approximate 50% reduction in computational time, indicating its potential utility in rapid screening while maintaining qualitative spectral features. In summary, in the case under consideration, in which the -OH groups were replaced by TMS groups during the synthesis, the SBAH-PO(OH)_2_+3(Si(CH_3_)_3_) model is more convenient and more justified in terms of calculation time.

In the spectra shown in Figure 3, there is a clear discrepancy between calculated vibrational frequencies and experimental measurements. To mitigate these discrepancies, the calculated frequencies are scaled by a correction factor [67]. This approach compensates for two main sources of error: the approximate nature of electronic structure calculations and the anharmonicity of the actual potential energy surface. The Morse potential better describes bond-stretching vibrations than the harmonic oscillator approximation for these modes. Scaling factors generally fall within the range of 0.85 to 1.1, depending on the class of compounds and the computational methodology employed. In this study, a simple scaling approach was adopted where reliably assigned reference peaks were used to determine a scaling factor minimizing the error between calculated and experimental spectra. The best agreement with experimental data was achieved by multiplying the theoretical frequency axis (x-axis) by a scaling factor of 0.9480. It is feasible to perform calculations that account for anharmonic oscillators; however, these require substantially greater computational resources. Incorporating anharmonicity results in spectral frequencies that more accurately correspond to experimental data, which are typically lower than those obtained under the harmonic oscillator approximation. Computations also include overtone transitions (e.g., v = 0 → v = 2 and v = 0 → v = 3) and combination bands, which arise from the sum or difference of frequencies of distinct vibrational modes [68,69]. For the system under investigation, even after simplifying the model to encompass only a few dozen atoms and reducing the basis set, it was not possible to carry out anharmonic spectral simulations using the Gaussian software package. An alternative approach for extending computational capabilities involves combining molecular dynamics simulations with density functional theory (DFT) calculations. This hybrid approach enables the investigation of conformational changes, as well as thermodynamic and kinetic processes. A notable example includes the simulation of surface-enhanced Raman scattering (SERS) spectra using atomic clusters [70].

Examining the parameters of the simulated spectrum (after editing the numerical simulation result file) reveals data such as frequency and intensity, which directly correspond to the spectrum. Additionally, information about atomic movement is available, aiding in visualizing the vibration. A line shape function (e.g., Voigt) is applied to each calculated frequency and intensity pair, and the sum of these individual functions yields a simulated spectrum that can be compared with experimental data.

**Figure 4:**
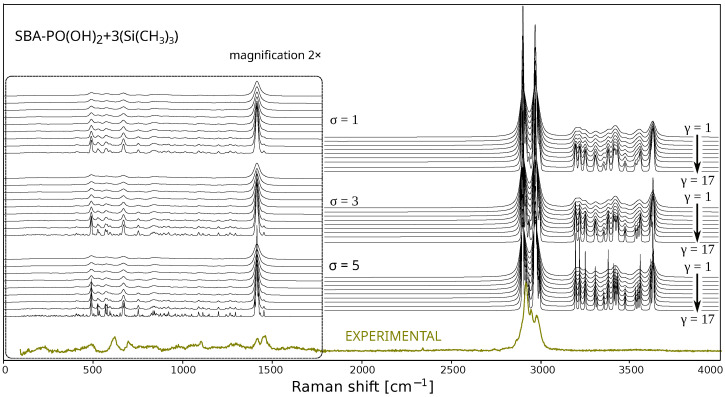
Simulated spectrum of SBA-PO(OH)_2_+3(Si(CH_3_)_3_) (B3LYP-D3(BJ)/6-31G(d)//B3LYP-D3(BJ)/6-31G(d)) obtained by applying different Voigt function parameters: *γ* from 1 to 17 in steps of 2 and *σ* = 1, 3, and 5. The offset of the theoretical spectra is achieved by multiplying the OX-axis by a scaling factor of 0.9480.

Figure 4 shows the simulated Raman spectra of SBA-PO(OH)_2_+3(Si(CH_3_)_3_), obtained using the B3LYP/6-31G(d) method with D3(BJ) scattering correction and different Voigt functions. It should be noted that the shape of the spectral lines reflects the characteristics of the adopted model: a purely damped harmonic oscillator generates Lorentz-shaped lines, but the actual lines are broadened in the direction of the Gaussian distribution by the influence of environmental factors, such as collisional broadening in the gas phase or the presence of structural defects in the crystals [71]. Therefore, in our considerations, we used the Voigt function (a convolution of Gaussian and Lorentzian distributions), believing it to be sufficient for a realistic representation of the spectra. The parameters γ and σ define the width of the Lorentzian and Gaussian components, respectively. The parameter γ represents the width of the Lorentzian component, which is usually defined as half the width at half-maximum intensity (HWHM). The parameter γ primarily influences the broadening of the spectral line wings and accounts for homogeneous broadening mechanisms such as natural radiative decay, collisional effects, and other processes related to oscillator dynamics. Conversely, the parameter σ defines the standard deviation of the Gaussian component, which predominantly shapes the central part of the spectral line profile. This parameter takes into account inhomogeneous factors, such as the influence of the instrument (e.g., spectrometer resolution), thermal oscillations, or statistical blurring of the line due to environmental dispersion [71]. The spectra shown in Figure 4 are the results of simulations using the Voigt function, with the value of the *γ* parameter modified from 1 to 17 in steps of 2, while the *σ* parameter was fixed at 1, 3, and 5 for the SBA-PO(OH)_2_+3(Si(CH_3_)_3_) model.

It is clearly evident that the appropriate choice of the broadening parameter, either *γ* (Lorentzian) or *σ* (Gaussian), is critical, as it significantly affects the shape of the simulated spectrum and its agreement with the experimental data. When spectral signals overlap, these differences become even more pronounced, potentially altering the maximum intensity of the individual peaks. It is important to note that experimental spectral features should not be treated as arising from functions with identical broadening parameters. For instance, the vibrational mode of a methyl group typically produces a sharp peak around 2980 cm^−1^, whereas -OH groups result in broader bands in the range of 3000–3400 cm^−1^, clearly illustrating the variability in spectral shapes. Additionally, the method of spectrum acquisition affects the observed profile. During measurements, resolution can be improved by using diffraction gratings of higher groove density (lines/mm). Another approach to improving spectral quality is to enhance measurement sensitivity and spatial resolution through techniques such as Surface-Enhanced Raman Spectroscopy (SERS) and Tip-Enhanced Raman Scattering (TERS) [72]. Since SERS enhances the inherently weak Raman signal with enhancement factors on the order of 10^10^–10^11^ through interactions between molecules and plasmonic nanostructures (e.g., roughened metal surfaces or nanoparticles), thereby enabling the detection of trace analytes down to the single-molecule level, TERS, which combines Raman spectroscopy with scanning probe microscopy (e.g., AFM or STM), achieves spatial resolution down to below 10 nm, thus overcoming the diffraction limit of conventional optical microscopy.

Based on the analysis of multiple spectra, we heuristically determined that the optimal fitting parameters for the employed equipment and measurement protocol are σ = 3.1 and γ = 3.1.

**Figure 5:**
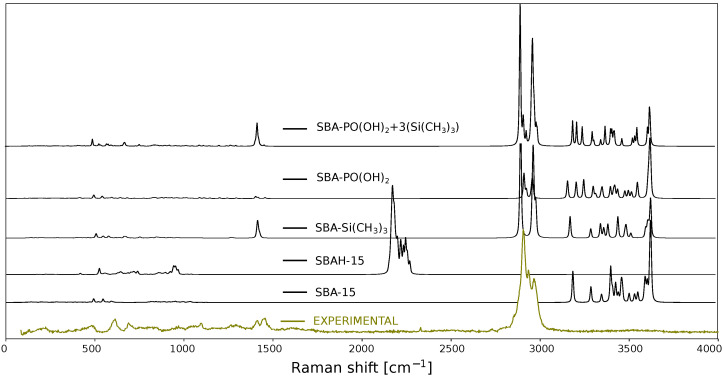
Comparison of theoretical Raman spectra determined from SBA-15, SBAH-15, SBA-Si(CH_3_)_3_, SBA-PO(OH)_2_, and SBA-PO(OH)_2_+3(Si(CH_3_)_3_) models with the experimental spectrum of the SBA-PO(OH)_2_ reference sample.

Once the theoretical spectrum has been obtained, the next crucial step is the assignment of vibrational signals to specific molecular motions or functional groups. Such assignments can be facilitated by the visualization of atomic displacements using dedicated software tools, such as GaussView 5.0 [73]. However, this approach becomes increasingly impractical for larger molecular systems due to their complexity and the sheer number of atoms involved. By analyzing vibrational modes derived from theoretical calculations, it is often possible to associate characteristic motions with well-defined functional groups [74,75]. For instance, symmetric stretching vibrations of methyl (-CH_3_) groups can be readily identified. In contrast, for more complex systems, such as silica-based materials, vibrational modes often involve collective deformations of larger structural fragments. This complexity makes direct band assignments challenging and frequently requires prior experience or detailed analysis of the full spectral region. Furthermore, when comparing calculated spectra with experimental Raman data for silica materials, notable discrepancies in signal intensities are often observed. These differences typically arise from the fact that computational models represent only a small fragment of the actual material, which limits the completeness and representativeness of the simulated spectrum. To overcome this limitation, spectral assignments can be improved by fragmenting the system into smaller, chemically meaningful units, allowing for more accurate identification of specific functional groups. Experimentally, this type of analysis can be performed by acquiring Raman spectra at various stages of the material synthesis; however, the changes observed then are not always unambiguous. Residual solvents or some impurities in the sample are enough to make the interpretation based on such a spectrum incorrect. Therefore, it is more accurate to prepare models corresponding to the material obtained in subsequent synthesis steps. This approach not only makes it easier to assign vibrations to the observed bands but also allows control of synthesis implementation correctness. Figure 5 shows the results of such calculations in the form of theoretical spectra for the SBA-15 silica structure fragment model with -OH groups and a simplified one (SBAH-15) by replacing hydroxyl groups with hydrogen atoms. Moreover, in Figure 5 are shown spectra of the SBA-Si(CH_3_)_3_ model, which contains a silica fragment in which the three -OH groups have been replaced by TMS groups (-O-Si(CH_3_)_3_), and the spectrum of the SBA-PO(OH)_2_ model, which consists of a silica fragment with a CH_2_CH_2_CH_2_PO(OH)_2_ functional group. The last spectrum in this collection is calculated for a model containing all the components (SBA-PO(OH)_2_+3(Si(CH_3_)_3_)) listed and corresponding to the experimental spectrum presented in this figure. All the models were geometrically optimized with the method B3LYP-D3(BJ)/6-31G(d), and the theoretical spectra were shifted relative to the experimental spectrum by multiplying by a scaling factor of 0.9480. Visualization of each model used for calculations of spectra presented in Figure 5 is available in the Supplementary Materials. When analyzing the aliphatic region at around 2980 cm^−1^ in the model SBA-PO(OH)_2_+3(Si(CH_3_)_3_), we see a clear overlap of signals from the methyl and propyl groups, which does not reflect the correct ratio of TMS to propyl-containing functional groups. Therefore, large systems require universal methods for controlling the quantitative proportions of functional groups.

As an alternative to such experimental and qualitative decomposition discussed above, we propose a new method combining molecular modeling and decomposition of theoretical Raman spectra, which is based on the presentation of bands derived from selected groups of model atoms in partial spectra. This approach involves assigning vibrational displacements to specific atoms within the model and using this information to generate partial spectra corresponding to individual atomic groups. Unlike separate calculations on isolated components, our method preserves the mutual interactions within the entire molecular framework, providing a more accurate representation of the vibrational environment.

**Figure 6:**
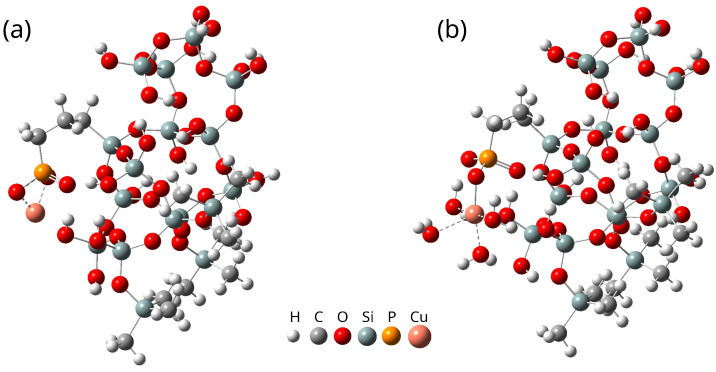
A model of the final material containing a fragment of silica with copper phosphonate functional groups (**a**) as a simple model SBA-POO_2_u+3(Si(CH_3_)_3_) and (**b**) with four molecules of water, which make the first solvation sphere.

By employing this decomposition strategy, it becomes significantly easier to assign Raman bands to specific atomic groups, thereby enhancing the interpretative power of theoretical spectra and their correlation with experimental data. Moreover, this approach requires less computational time than separate calculations of models representing materials at different synthesis stages. In this study, we focus on the preparation and characterization of a structural model representing the final material. As shown in Figure 6a, the model includes a silica fragment functionalized with copper phosphonate groups. This model is a simplified representation of the investigated system, in which molecules such as water and other components of the copper solvation environment have been intentionally omitted for methodological clarity. For comparison, Figure 6b presents an extended version of the model, incorporating four water molecules coordinated to the copper center. These water molecules form the first solvation shell, which plays a crucial role in determining the physicochemical properties of the material. A detailed discussion of the copper coordination environment and the impact of solvation effects on the system’s behavior can be found in our previous work [19].

**Figure 7:**
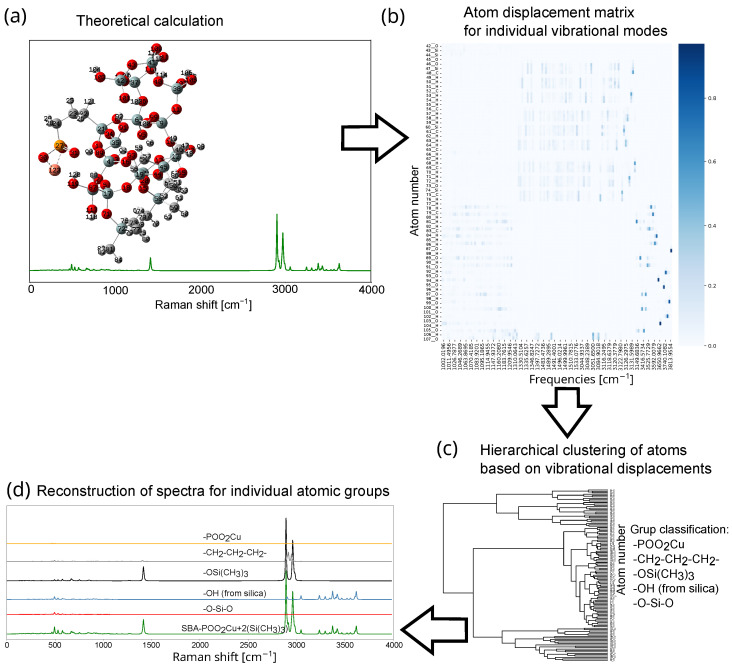
Flowchart of the procedure for decomposing the theoretical spectrum. Stages: (**a**) Structure optimization and calculation of the theoretical spectrum. (**b**) Part of atom displacement matrix for individual vibrational modes. The x-axis represents the atoms in the molecule, and the y-axis represents individual vibrations. The calculated contribution of a given atom to the vibration is indicated in color on a scale of 0–1. (**c**) Automatic grouping of atoms using hierarchical clustering (dendrogram) (or using a large language model or manually), dividing the structure into these fragments. (**d**) Reconstruction of spectra for individual atom groups. Illustrations of each of the steps are included in the Supplementary Information.

In a typical theoretical Raman spectrum for a silica-based model, over one hundred distinct vibrational modes can be observed, with each mode involving the motion of multiple atoms. The interpretation of such a large number of vibrational contributions presents a significant analytical challenge. To streamline this process, we propose a method based on a quantitative analysis of the atomic displacement vectors corresponding to each vibrational mode (Figure 7). These vectors are obtained from quantum chemistry software packages such as Gaussian [49]. It is possible to analyze individual vibrations based on redundant internal coordinates (in Gaussian calculations, this is performed using the freq = intmodes option). This provides information about normal modes, representing changes in selected redundant internal coordinates such as distances, angles, and dihedral angles, along with their calculated relative weights expressed in percent. For simple molecules, such analysis may be sufficient, but it is incomprehensible for more complex structures.

For each vibrational mode, atomic displacements are provided in the form of three-dimensional vectors (Δx, Δy, Δz). To simplify interpretation, we compute the scalar displacement magnitude for each atom using the expression $l_{NA}=\sqrt{\Delta x^{2}+\Delta y^{2}+\Delta z^{2}}$. This scalar value removes directional information, facilitating a direct comparison between atoms or atomic groups irrespective of their spatial orientation. Since each vibrational mode involves displacements of multiple atoms, we normalize each atomic displacement by the total displacement across the molecule: $l_{NA}^{\prime }=l_{NA}/\sum_{i=1}^{n}l_{NA_{i}}$ This transformation enables identification of atoms with the largest relative displacements in each mode. In many computational chemistry packages, atomic displacement data are provided in a normalized form.

The presented analysis is based on calculated vibrational amplitudes described in Cartesian coordinates [76,77,78]. Although this approach is widely used, a better method for describing vibrational modes is the application of internal coordinates. Internal coordinates more accurately represent curvilinear motions, facilitate the analysis of vibrational energy, and improve the intuitive assignment of vibrations [76,77,78]—especially when using primitive internal coordinates of the Wilson type (e.g., bond lengths, bond angles, and dihedral angles) [79]. However, the use of internal coordinates is also associated with certain limitations, which result from their complexity and potential ambiguity, particularly in the case of large molecules. In this study, because the analysis centers on individual vibrational modes and their association with specific atomic groups, the use of Cartesian coordinates offers a clearer and more straightforward framework for interpretation. Examining atomic displacements in three-dimensional space enables the precise identification of each atom’s contribution to a given vibration, yielding assignments of vibrational modes that are equally accurate to those obtained using internal coordinates.

The proposed approach can be further refined by incorporating atom-type-specific normalization factors or weighting schemes (another solution could be to use a different Voigt function depending on the atom). Similar strategies have been successfully employed in correlating transmission electron microscopy (TEM) images with molecular modeling, where atomic radii were adjusted to best match experimental observations [80]. By applying this method across all vibrational frequencies, we achieve an unambiguous assignment of vibrational motions to specific atoms, thereby enhancing the interpretability of complex vibrational spectra.

The flowchart of the spectrum decomposition procedure is illustrated in Figure 7 and consists of four main steps. First, the molecular geometry is optimized, and the theoretical vibrational spectrum is computed (Figure 7a). Subsequently, a displacement matrix is generated for all atoms involved in each vibrational mode (Figure 7b). Based on the displacement vectors, atoms are grouped using hierarchical clustering (dendrogram) analysis (Figure 7c), allowing the system to be partitioned into functionally meaningful fragments (e.g., CH_3_ groups). This classification can be performed either manually or through automated clustering algorithms, which identify atoms with similar vibrational characteristics. In addition, we explored the application of a large language model (e.g., LLaMA [81]) to identify molecular fragments such as -CH_2_CH_2_CH_2_- and -Si(CH_3_)_3_ directly from Cartesian coordinates. The model showed promising results in recognizing common molecular motifs, although occasional misclassifications were observed. The final step in the procedure involves reconstructing the vibrational spectrum for selected molecular fragments. This is achieved by summing the individual contributions of the selected atoms to the intensity of each vibrational mode. The result is then convolved with a Voigt profile to generate a smooth and interpretable fragment-specific spectrum (Figure 7d).

**Figure 8:**
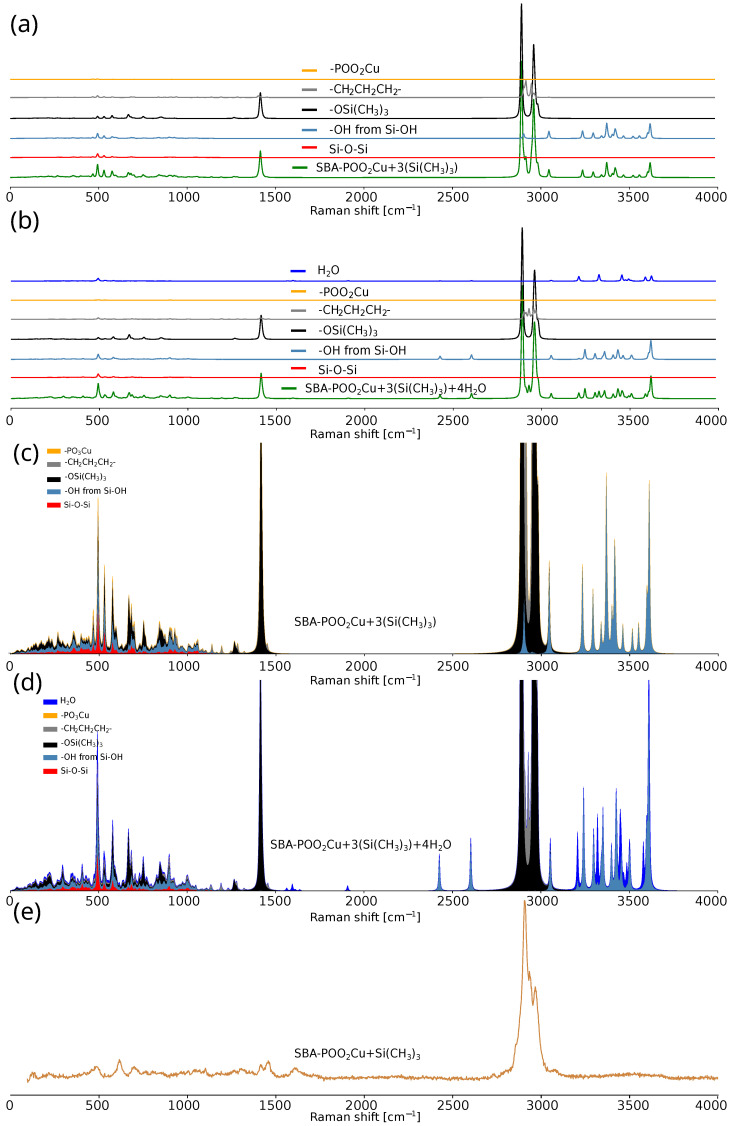
Theoretical spectra of SBA-POO_2_Cu+3(Si(CH_3_)_3_) (**a**) and SBA-POO_2_Cu+3(Si(CH_3_)_3_)+4H_2_O (**b**) split into specific fragments, cumulative stacked area chart of SBA-POO_2_Cu+3(Si(CH_3_)_3_) (**c**) and SBA-POO_2_Cu+3(Si(CH_3_)_3_)+4H_2_O (**d**) theoretical spectra, and experimental spectrum of SBA-POO_2_Cu+Si(CH_3_)_3_ (**e**).

The comparison of theoretical and experimental Raman spectra of SBA-POO_2_Cu+Si(CH_3_)_3_-based materials is presented in Figure 8. Figure 8a,b show the theoretical Raman spectra of SBA-POO_2_Cu+3(Si(CH_3_)_3_) and SBA-POO_2_Cu+3(Si(CH_3_)_3_)+4H_2_O, respectively, with decomposition into fragment-specific contributions. Molecules analyzed are shown in Figure 6. Figure 8c,d present the same theoretical data as cumulative stacked area charts, which facilitate the visualization of vibrational mode contributions from individual molecular fragments. The experimental spectrum of SBA-POO_2_Cu+Si(CH_3_)_3_ is provided in Figure 8e for comparison.

The differences between the theoretical spectra for the models with and without the first solvation shell (H_2_O molecules) highlight the influence of weakly polarizable water molecules, whose contribution to the total Raman signal remains minimal. The silica scaffold, relative to its number of atoms in the model, exhibits weak Raman intensity. Interestingly, the -POO_2_Cu moiety exhibits exceptionally low Raman activity. Most of the calculated vibrational intensities originate from aliphatic chains and labile groups, suggesting their dominant role in the overall spectral profile. The relative contributions of the trimethylsilyl and propyl groups appear disproportionate, which can be corrected by rescaling their spectral intensities. Moreover, incorporation of the solvation shell causes noticeable shifts in the vibrational modes of the propyl group, emphasizing the importance of local environment in spectral interpretation. In summary, the visualization of individual vibrational modes significantly facilitates the assignment of bands in experimental spectra. This approach enhances interpretability by directly linking spectral features to specific molecular fragments and their motions, thus improving the accuracy and confidence of vibrational mode attribution.

**Figure 9:**
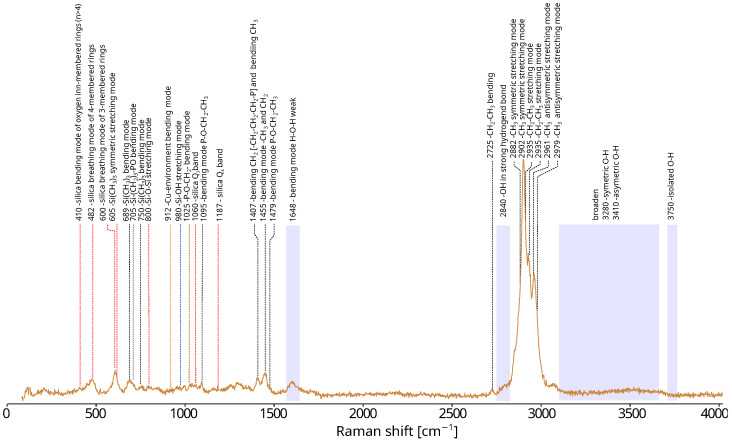
Experimental spectrum of silica SBA-POO_2_Cu thin film with annotated vibrational band assignments.

In conclusion, our proposed method—decomposing the theoretical spectrum into components corresponding to the individual atomic groups—enables rapid spectral analysis and accurate assignment of vibrational bands in the experimental data. Its practical usefulness is demonstrated by the example of the analysis of the experimental spectrum of a copper phosphonate-functionalized silica thin film (Figure 9).

The Raman spectrum of the SBA-POO_2_Cu+Si(CH_3_)_3_ composite reveals characteristic bands associated with the silica framework, primarily in the 400–1200 cm^−1^ region, corresponding to various vibrational modes of silica-based ring structures (macrocyclic siloxane units). Bands from the bending vibrations of oxygen atoms in rings containing more than four silicon atoms can be observed near 410 cm^−1^, along with bands associated with the breathing mode of four-membered siloxane rings (at 490 cm^−1^) and a band corresponding to the breathing vibrations of three-membered silica rings (at 605 cm^−1^). In the spectral range between 850 and 1060 cm^−1^, several peaks can be identified and are commonly ascribed to tetrahedrally coordinated silicon atoms bonded to four (850 cm^−1^), three (900 cm^−1^), two (950–1000 cm^−1^), or one (1050–1100 cm^−1^) bridging oxygen atom. Additionally, the signal at approximately 980 cm^−1^ is typically linked to the vibrational mode of hydroxyl (-OH) groups bound to silicon atoms. The incorporation of trimethylsilyl groups is evidenced by bands at 615, 695, 763, 1102, 1271, 1417, 2910, and 2970 cm^−1^. Notably, bands in the 2900–3000 cm^−1^ range overlap with signals attributed to copper phosphonate groups, suggesting the coexistence of these functional entities within the material structure. Broad bands in the 3000–3700 cm^−1^ region reflect the presence of water molecules and hydroxyl groups—a common feature in silica materials such as SBA-15. The band near 2840 cm^−1^ likely arises from a hydroxyl group engaged in strong hydrogen bonding, either as part of a dimer involving phosphorus-bound -OH groups or due to a water molecule interacting with the phosphonic acid moiety. A weak band around 1600 cm^−1^ further supports the presence of molecular water, corresponding to O–H bending vibrations.

The presented example demonstrates the effectiveness of the theoretical spectral decomposition method, which eliminates the need for large-scale modeling or step-by-step molecular construction calculations. We successfully validated the proposed spectral decomposition approach in simulations involving various concentrations of copper phosphonate groups, in the assignment of signals in mesoporous materials [19,20], in mixed SiO_2_–TiO_2_ systems [17], and in the analysis of Raman spectra related to reactive oxygen species (article in preparation). Additionally, it was applied during the initial development stage of the AI Agent system in an autonomous Raman laboratory (article in preparation) and in the analysis of IR spectra (article in preparation). Technical details of the method, along with a practical example in the form of a Jupyter-lab notebook, are provided in the Supplementary Materials. Importantly, a single decomposition model can approximate spectra across different stages of synthesis. The methodology can also be extended in the same manner to the analysis of IR spectra. With a sufficiently large dataset of spectral fragments, neural networks could be employed for fully automated spectral prediction, bypassing the need for conventional molecular modeling. This example highlights how appropriate computational methods can significantly accelerate the spectral analysis process.

## 3. Materials and Methods

In order to demonstrate the method of analyzing the structure of functional nanomaterials based on molecular modeling, we prepared a precisely functionalized material based on porous silica. This is mesoporous silica SBA-15, containing copper phosphonate functional groups distributed homogeneously within the pores prepared in the form of a thin film (SBA-POO_2_Cu+Si(CH_3_)_3_), as illustrated in Figure 1. A reference sample, labeled SBA-PO(OH)_2_+Si(CH_3_)_3_, was also prepared as a silica thin film, representing the material at the penultimate stage of the synthesis. The preparation procedure of the material involved a few steps to obtain copper phosphonate groups inside the silica matrix based on literature reports [82,83]. The thin films were deposited using a dip-coating method. This method involves submerging a substrate in a coating solution and then pulling it out to create a uniform film. In this case, glass supports were used. The sol solution was prepared according to the following procedure. In the first step, a solution of ethanol and water in a weight ratio of 2.5:2 was prepared, followed by adjusting the pH to 1.25 by adding HCl. Next, to this solution (5 mL), tetraethyl orthosilicate (TEOS, Sigma-Aldrich, Darmstadt, Germany) and phosphonate propyl diethyl triethoxysilane (PPTES, Sigma-Aldrich) were added in molar proportions of 85.31:4.49. After that, the solution was kept stirring for 2 h. Simultaneously, a second solution consisting of 70 mL of a 3 mM ethanol solution of triblock copolymer Pluronic P123 (EO_20_PO_70_EO_20_, where EO is poly-ethylene oxide and PO is poly-propylene oxide, Sigma-Aldrich) was prepared in a separate container. The second solution was also kept stirring for 2 h. Both solutions were combined, and the obtained sol was stirred for another 3 h. After this time, 4 g of deionized water with pH 1.25 (adjusted by adding HCl) was added to the solution. After aging the sol for 2 h, the dip-coating deposition of the thin film started.

To obtain thin films with the desired thickness and good quality, the relevant parameters of the dipping procedure (relative humidity of 75% and a temperature of 22 ^∘^C) were set in the closed-chamber dip-coater. The glass substrates were dipped into the sol solution and withdrawn at a constant speed of 15 cm per minute. After that, the substrates were left for 20 min in the chamber for proper thin-film formation. Next, the thin films obtained were aged overnight at 100 ^∘^C. Samples obtained in this procedure are the thin silica films with the surfactant and precursors of functional groups inside the pores. To complete the functionalization, firstly, the surfactant was removed by ethanol extraction. At this stage, the silica matrix contains phosphonic ester groups, which should be transformed in the next steps to copper phosphonate groups. These steps involve a silylation procedure (substitution of -OH groups with trimethylsilyl groups (TMS) groups) and hydrolysis.

During the silylation procedure, silica thin films were immersed in 221 mM chlorotrimethylsilane (ClTMS) toluene solution for 12 h. After that, samples were washed with toluene and dried. Next, hydrolysis was performed to convert ester units into phosphonic acid groups. Silica samples on glass substrates were immersed in a 1M solution of HCl for one hour and then rinsed with distilled water and dried. The last step is the introduction of copper ions to the silica matrix. In this step, samples were put into a saturated solution of Cu(acac)_2_ in tetrahydrofuran for 12 h. The samples were then washed with hot tetrahydrofuran for 5 h to remove excess copper salts. Then the samples were dried under vacuum at 100 ^∘^C.

Raman spectra were recorded using a WITec confocal Raman microscope (CRM alpha 300) equipped with an air-cooled solid-state laser (λ = 532 nm) and a CCD camera (ANDOR iVac DR-316B-LDC-DD-35B, Oxford Instruments, Abingdon, UK). An air Zeiss EC Epiplan-Neofluar Dic, Carl Zeiss Microscopy GmbH, Jena, Germany 100×/0.9 lens was used. Raman scattered light was focused onto a multimode fiber and a monochromator with a 600 line per mm grating. The spectra were collected at room temperature. To avoid the strong influence of the glass substrate on the recorded Raman spectra, thin-film samples were scraped from the substrate and observed in a substrate-free form.

Numerical models of the molecules assumed to exist in the materials studied were used to correctly interpret the Raman spectra. The models were built in the program GaussView [73] and visualized. Due to the nature of the SBA-15 material, which is mesoporous, molecular models were constructed by placing SiO_4_ groups in a way that closely resembles a stochastic arrangement, while avoiding repeated elements. The resulting model was then optimized with -OH groups attached. Subsequently, appropriate groups were added to the surface, or hydroxyl groups were replaced by hydrogen atoms, followed by molecular optimization. No molecular parameters, such as distances or angles, were constrained during this process. For systems containing copper ions, two models were prepared: one including only the copper atom and the other featuring a copper atom surrounded by four water molecules simulating the first solvation shell. Water molecules play an important role in the environment surrounding metal ions [19,84,85]. Geometry optimization and vibrational spectra calculations of the model molecules were carried out with the Gaussian G16 Revision C.01 program package [49] using Becke’s three-parameter (B3) hybrid functional [86] with the Lee–Yang–Parr [87] correlation functional with Grimme’s dispersion and the Becke–Johnson damping parameter with standard 6-31G(d) and the def2TZVP basis set [88]. The absence of imaginary vibrational frequencies confirms that the optimized molecular geometries are located at a local minimum on the potential energy surface. Numerical models of the molecules assumed to exist in the materials studied were used to correctly interpret the Raman spectra. The detailed procedure, including computational files, structures, and a sample notebook, is provided in the Supplementary Materials.

All data analysis was performed using Python version 3.11.2 and standard scientific packages such as Pandas 2.3.3, SciPy 1.14.0, NumPy 2.3.0, Pylab 3.10.0, Matplotlib 3.10.0, and JupyterLab 4.3.4 [89,90,91,92].

## 4. Conclusions

Raman spectroscopy is an efficient analytical technique for studying functional materials. However, given the specific nature of materials containing insignificant amounts of functional groups, interpreting Raman vibration spectra is often complicated due to the lack of appropriate references. The solution is to use molecular modeling, which allows us to obtain theoretical structures that we predict exist in the material. Molecular modeling enables the visualization and identification of molecular vibrations in the structure. By superimposing experimental and theoretical spectra, the process of analyzing the structure of the material under investigation is greatly facilitated. However, this does not guarantee complete accuracy in the analysis, especially for materials that give a relatively weak Raman signal, as is the case with thin films. To increase its accuracy, we have proposed the use of decomposition-based analysis of theoretical Raman spectra for enhanced interpretation of experimental data.

In this work, we present an efficient and versatile method for the decomposition of theoretical Raman spectra into components specific to atomic groups. By separating the vibrational contributions of individual molecular fragments, this approach allows us to link specific parts of the spectrum to defined structural elements. In this way, each spectral feature can be clearly linked to a specific molecular mode. As a result, interpreting experimental data becomes easier and more accurate. The proposed methodology significantly optimizes computational resources, minimizing the need for large-scale molecular modeling, and can be adapted to a wide range of functionalized nanomaterials.

Furthermore, the modular design of the decomposition structure ensures its applicability beyond Raman spectroscopy, also offering a unified vibrational analysis strategy for IR spectroscopy. The integration of this method with advanced computational techniques, such as density functional theory (DFT) and machine learning models, holds great promise for the development of autonomous laboratories capable of high-throughput spectral prediction and materials discovery. Overall, our approach advances the field of computational spectroscopy by providing a powerful tool to accelerate and improve spectral analysis processes.

## Data Availability

Source data are available on request.

## Supplementary Materials

The following supporting information can be downloaded at https://www.mdpi.com/article/10.3390/ijms262010237/s1.

## Abbreviations

The following abbreviations are used in this manuscript:

AI	Artificial intelligence
B3LYP	Becke, 3-parameter, Lee–Yang–Parr
D3(BJ)	Grimme’s dispersion and the Becke–Johnson damping parameter
DFT	Density functional theory
HWHM	Half-width at half-maximum
LLM	Large language model
NA	Numerical aperture
SBA-15	Santa Barbara Amorphous-15
SERS	Surface-Enhanced Raman Spectroscopy
TEOS	Tetraethyl orthosilicate
TEM	Transmission electron microscopy
TERS	Tip-Enhanced Raman Scattering
TMS	Trimetylosilan
PPTES	Phosphonate propyl diethyl triethoxysilane
P123	(Poly(ethylene glycol)_20_-poly(propylene glycol)_70_-poly(ethylene glycol)_20_
