# Supplementary material for: Decomposition Analysis of Theoretical Raman Spectra for Efficient Interpretation of Experimental Spectra of Thin-Film Functional Materials
Source: Int J Mol Sci. 2025 Oct 21;26(20):10237. doi: 10.3390/ijms262010237 (PMC12564597; doi:10.3390/ijms262010237)
Supplement: Supplementary file 1 [file ijms-26-10237-s001.zip › ijms-3899220-supplementary.pdf]

Supplementary Information for:

Decomposition analysis of theoretical Raman spectra for efficient interpretation of experimental spectra of thin-film functional materials

Marek Doskocz, Łukasz Laskowski, Jacek Kujawski, Agnieszka Karczmarska, Krzysztof Cpałka, Ewelina Lipiec, and Magdalena Laskowska

October 20, 2025

This PDF file includes:

Contents

1 Step one - Preparing calculations **2**

2 Step two - Calculation **7**

3 Step three - Analysis of the movements ot the atoms **11**

4 Step four - Grouping of atoms **12**

5 Step five - Visualization data **13**

6 Example as a Jupyter-Lab notebook **14**

7 Structural database of structures from publication **121**

7.1 SBA-15 121
7.2 SBAH-15 124
7.3 SBA-$Si(CH_3)_3$ 126
7.4 SBA-$PO(OH)_2$ 130
7.5 SBA-$PO(OH)_2$+3($Si(CH_3)_3$) 133
7.6 SBA-POO_2Cu+3($Si(CH_3)_3$)+4H_2O 137
7.7 SBA-POO_2Cu+3($Si(CH_3)_3$) 141

We present the most important stages of preparing and analyzing calculations RAMAN and IR.

1 Step one - Preparing calculations

We can download the structure from the database/publication [1, 3, 4], or construct a model for calculations ourselves. Then we need to perform calculations in the Gaussian[2] program. First is optymalization, the second step is calculation of frequency.

Below is an example input file used for calculations in the Gaussian program.

```
%chk=SBA15_Dh2o_D4_173_50_FREQ_2_Cu_4H2O_FREQ.chk
#p freq=raman ub3lyp/6-31g(d)  empiricaldispersion=gd3bj

for pub AI Raman , Marek Doskocz 2025

0 2
 O                  0.81108600   -1.74794000   -2.08954200
 O                  2.86317800   -0.16764000   -2.69549300
 Si                 3.77922500   -0.60775200   -1.41403400
 Si                 1.21325800   -0.28447700   -2.70797400
 O                  3.20732200    0.19127400   -0.09076800
 Si                -2.62733600   -0.17381500   -1.98386800
 O                 -2.70586000    0.59494300   -0.54794200
 O                 -1.78984000   -1.55719700   -1.87769200
 Si                -3.17744700    1.53630800    0.68311700
 O                 -2.20757200    2.84481300    0.77176600
 O                 -4.71625200    2.06261100    0.44537600
 Si                -1.61027700    3.90743700   -0.33605900
 O                 -0.79500400    3.05541700   -1.49587600
 Si                 0.73750900    2.43026100   -1.43981700
 O                  1.67196000    3.10941000   -2.56445800
 O                  0.60626200    0.82872500   -1.65347800
 Si                 3.86840000    0.64088600    1.34497100
 O                  2.86389100    1.76831000    1.99616600
 Si                 1.32665700    2.15828700    1.56363200
 O                  1.34573300    2.74550500    0.04600700
 Si                -0.44836600   -2.17330900   -1.09633800
 C                 -0.77553000   -3.99490900   -0.82354400
 C                 -0.62891700   -4.50231100    0.64255600
 C                  0.61377900   -5.37162600    0.93941000
 H                 -1.51719900   -5.08153900    0.90585300
 H                 -0.62644400   -3.65572300    1.33449000
 P                  2.12230900   -4.64831700    0.22574300
 H                  0.73612700   -5.49133700    2.02136100
 H                  0.50923500   -6.37127700    0.50530100
 O                  3.38972600   -4.82911400    1.15705500
 O                  1.83474200   -3.10229600    0.15074300
 O                  2.38156700   -5.26830200   -1.15426600
 O                  0.36745700    0.85329100    1.70554500
 O                 -0.38896500   -1.29604100    0.28895500
 Si                 0.12719900   -0.76394800    1.71483800
 O                 -4.14901800   -0.57472800   -2.45366600
```

Si	-5.11873500	-1.86403000	-2.16688400
Si	-6.15065100	1.28830400	0.38484100
O	-4.68591200	-2.64259600	-0.80275700
O	-5.85769500	-0.29378200	0.70211700
Si	-6.89031300	-1.46646700	1.27034500
Si	-4.45354900	-2.97307900	0.77872400
O	-5.90193400	-2.74448500	1.55493600
O	0.75418800	3.37131000	2.48776800
Si	0.13771800	3.41557000	4.07438200
C	-1.70692800	3.70996400	3.94556500
H	-2.14224200	3.90390600	4.93328800
H	-1.92253300	4.57086100	3.30324300
H	-2.21494200	2.84374200	3.51160600
C	1.02063300	4.84742700	4.90344700
H	0.67698400	4.98566200	5.93541400
H	2.10291500	4.67599200	4.92868500
H	0.84217900	5.78378900	4.36241500
C	0.52567300	1.78125000	4.91499600
H	-0.05673600	0.95914800	4.48657600
H	1.58868800	1.52804800	4.82370300
H	0.28974300	1.83582300	5.98462200
Si	1.76359300	4.63931000	-3.27482100
C	1.89100500	5.91460800	-1.90150100
H	1.92896200	6.93115300	-2.31175200
H	1.03242000	5.85864000	-1.22255500
H	2.79656000	5.75876800	-1.30478600
C	0.20436600	4.89478700	-4.28749400
H	0.09284300	4.10300700	-5.03734900
H	-0.68307200	4.87671900	-3.64513500
H	0.22141800	5.85670000	-4.81376700
C	3.30381400	4.60110100	-4.34207600
H	4.19342800	4.41157200	-3.73122400
H	3.23594800	3.80396000	-5.09072400
H	3.45008300	5.55119600	-4.86951600
O	5.34885700	1.26350100	1.04962000
Si	5.79148300	2.87672000	0.72092500
C	4.77416900	3.44095700	-0.75222100
H	3.70310700	3.43756000	-0.52824700
H	5.05091600	4.46017500	-1.04842000
H	4.92386900	2.79360800	-1.62472400
C	5.45955600	3.92255100	2.23935400
H	4.39333900	3.91393000	2.48729000
H	6.01198500	3.54406800	3.10682600
H	5.76450300	4.96265500	2.07336800
C	7.61723900	2.78903800	0.30140000
H	8.19312200	2.37125700	1.13464200
H	7.79638500	2.16092800	-0.57957500
H	8.01761500	3.78567800	0.08119500
O	-0.67641900	4.97661700	0.47664000
H	-0.11269400	4.58080700	1.17144400
O	3.98453000	-0.64409400	2.37010700
H	3.05801700	-0.99642300	2.50339600
O	1.50145000	-1.56208500	2.14566100
H	1.70123000	-2.25667200	1.41292700
O	-1.11048400	-0.93857400	2.80773300
H	-1.71832900	-1.66887000	2.56364200
O	0.64707500	0.00324000	-4.20914800
H	-0.29970200	0.24892500	-4.17603200
O	-1.97781000	0.85263600	-3.10438900

H	-1.72222500	1.71341700	-2.71904800
O	-2.78939300	4.66335400	-1.18514000
H	-3.48389900	5.08254200	-0.65868500
O	-3.17041800	0.68778900	2.08863200
H	-2.31252100	0.34848200	2.44079000
O	-3.30058800	-2.04461600	1.49071500
H	-3.53126900	-1.09477500	1.59009800
O	-3.95430200	-4.52545200	0.93556100
H	-4.21512300	-5.09839000	0.20103000
O	-7.26422700	1.89477000	1.42427200
H	-7.52624800	1.27168900	2.12580900
O	-6.75373900	1.41300400	-1.16138800
H	-7.63551000	1.81148300	-1.20309300
O	-8.09551200	-1.72074800	0.20974800
H	-7.77740800	-1.70742700	-0.72178300
O	-7.57502100	-0.91694100	2.67040000
H	-7.17682900	-1.26147000	3.48197900
O	-6.65557700	-1.24494600	-2.09030500
H	-6.66880900	-0.27188200	-1.94214900
O	-5.05769600	-3.02297600	-3.32551800
H	-5.39125500	-2.75176200	-4.19197000
O	5.39992200	-0.25269800	-1.57260200
H	5.68223100	0.40661200	-0.91271500
O	3.75571400	-2.21981400	-1.18919300
H	2.86296200	-2.55251700	-0.79314500
H	-1.80809100	-4.15025800	-1.15751000
H	-0.13932000	-4.57466200	-1.50199500
Cu	4.87890000	-3.83220000	0.42217100
O	6.31786800	-2.48765900	-0.04827600
H	6.13404600	-1.82876200	-0.75615100
O	4.99982100	-3.15447400	2.27198600
H	4.20336300	-3.60253600	2.62014800
O	4.76848600	-4.66970700	-1.39305900
H	3.76833600	-5.07094800	-1.39580800
O	6.85905600	-5.14861200	0.16958100
H	7.02000600	-5.90677900	0.75107300
H	6.39103900	-5.49274200	-0.61595800
H	7.14572700	-2.95822500	-0.23811700
H	4.82969500	-2.17864300	2.33462900
H	4.65509700	-3.87828700	-1.95417200

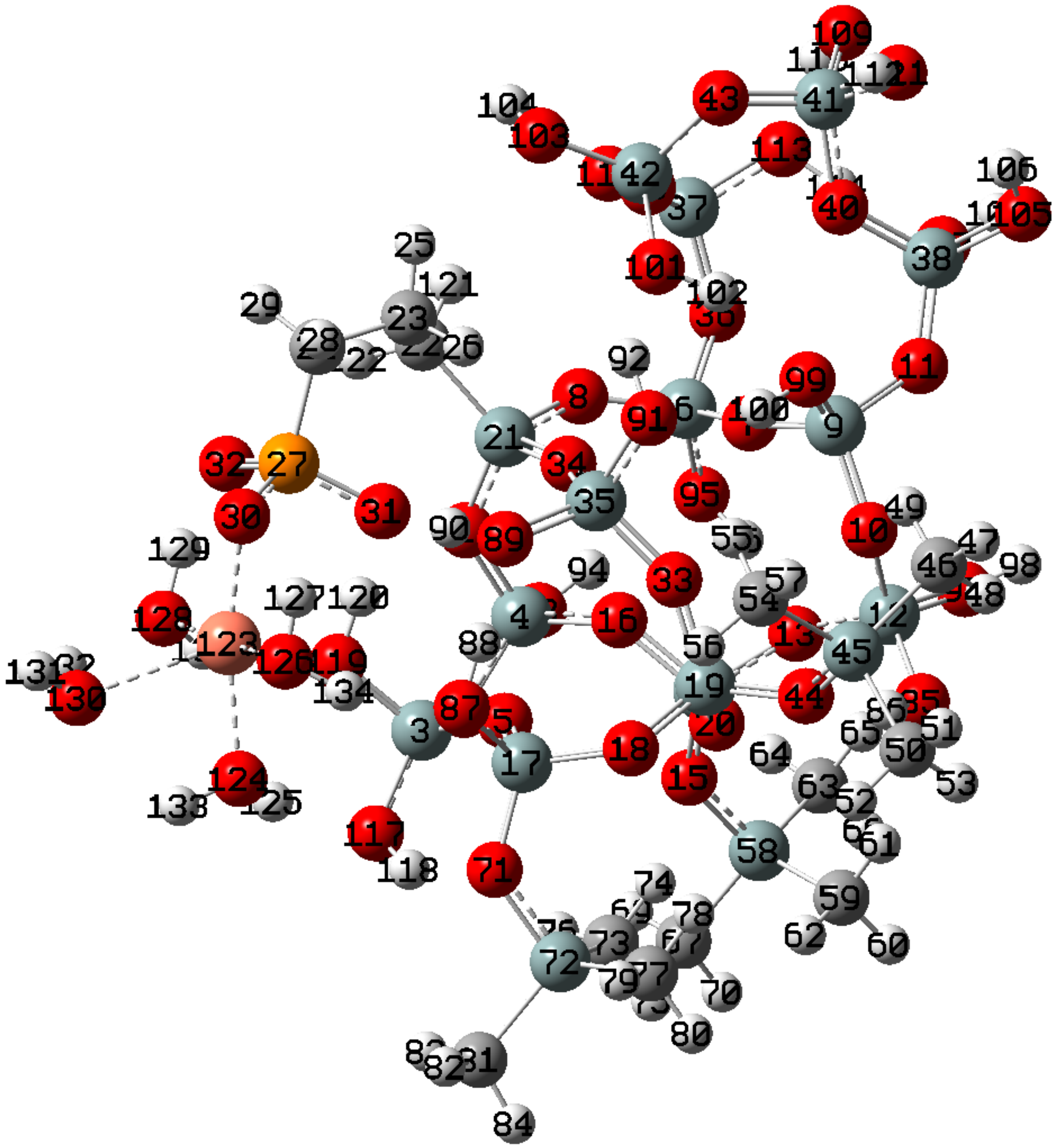

Figure S1: The stable structures of SBA-POO_2Cu+3($Si(CH_3)_3$)+4H_2O.

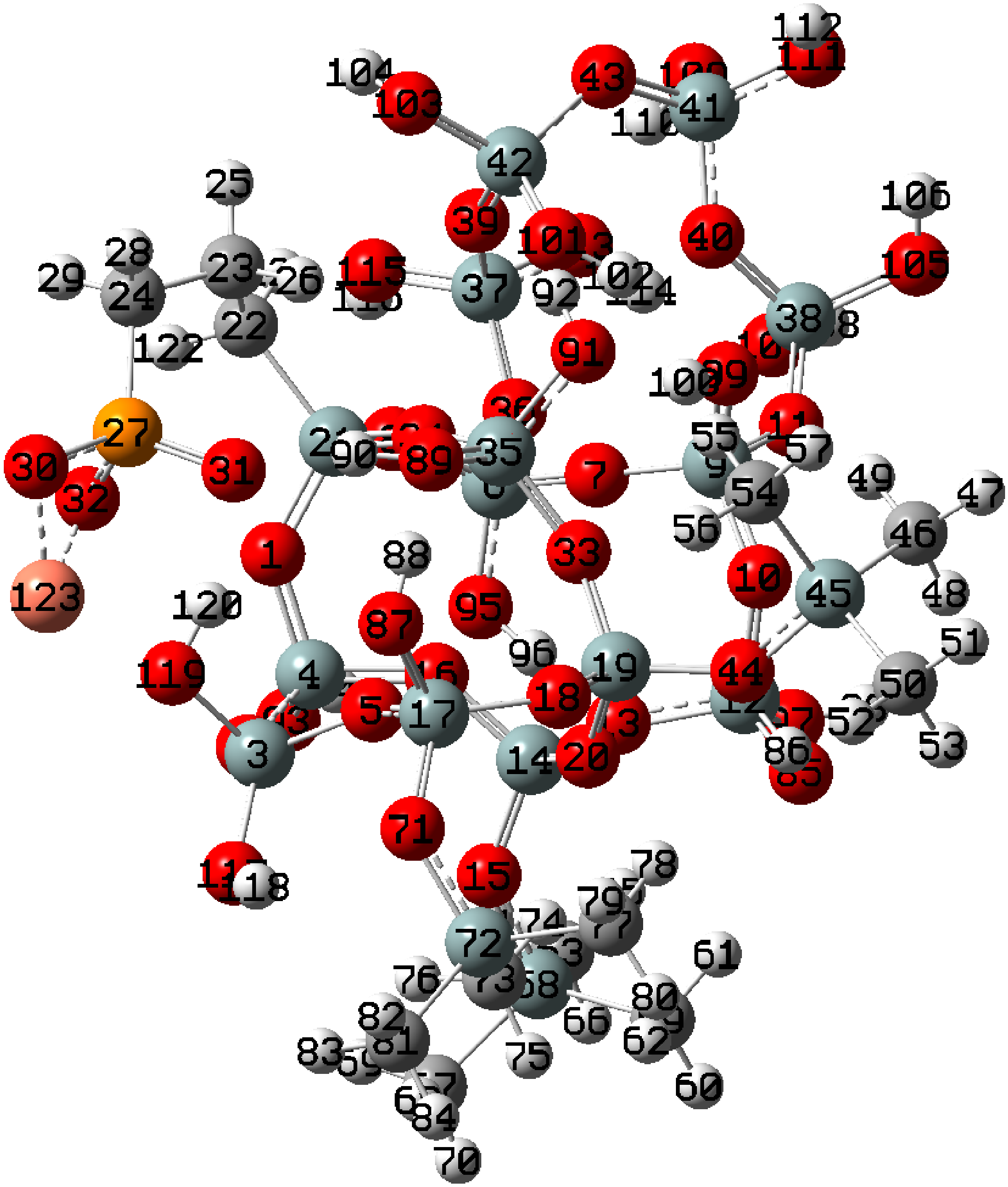

Figure S2: The stable structures of SBA-POO_2Cu+3($Si(CH_3)_3$)

2 Step two - Calculation

After performing the calculations, we should receive a log result file, which will contain the simulation results. We check whether we do not have an imaginary frequency. If we do, we need to correct the system and perform optimization and frequency calculations again.

Below is an example of a fragment of a output file containing information about the calculated frequencies.

```
Leave Link  703 at Thu Apr 24 15:17:02 2025, MaxMem=  8053063680 cpu:    30894.2 elap:    647.9
(Enter /opt/exp_soft/local/skylake/gaussian/g16/C.01/l716.exe)
Dipole        = 2.18142217D+00 9.54102590D-01 1.42869262D+00
Polarizability= 6.29144821D+02 5.61597620D+00 5.83652331D+02
               -8.00418798D-01 1.60468010D+00 5.22938965D+02
HyperPolar    = 3.31244891D+02-6.02457264D+01 7.56000357D+01
                1.40693342D+02 5.75618097D+01-3.30657182D+01
               -1.57105711D+02-1.79111643D+01-1.70238389D+00
                1.25981463D+02
Full mass-weighted force constant matrix:
Low frequencies ---   -2.2722   -1.4143   -0.0068   -0.0053   -0.0043    1.9805
Low frequencies ---   13.6277   18.0998   22.8399
Diagonal vibrational polarizability:
     948.4622909     817.3931614     459.9435610
Diagonal vibrational hyperpolarizability:
     210.5766220    1113.1346365     -52.2608700
Harmonic frequencies (cm**-1), IR intensities (KM/Mole), Raman scattering
activities (A**4/AMU), depolarization ratios for plane and unpolarized
incident light, reduced masses (AMU), force constants (mDyne/A),
and normal coordinates:
                     1                      2                      3
                     A                      A                      A
Frequencies --     13.6203                18.0943                22.8358
Red. masses --      4.3716                 3.9545                 7.5408
Frc consts  --      0.0005                 0.0008                 0.0023
IR Inten    --      0.3158                 0.2056                 1.0682
Raman Activ --      0.1749                 0.0587                 0.0650
Depolar (P) --      0.6833                 0.7492                 0.7457
Depolar (U) --      0.8118                 0.8566                 0.8544
 Atom  AN      X      Y      Z        X      Y      Z        X      Y      Z
    1   8     0.00   0.00   0.01    -0.01  -0.02  -0.00    -0.01   0.00  -0.05
    2   8     0.00  -0.01  -0.01    -0.00  -0.01   0.00     0.00  -0.00  -0.02
    3  14     0.00  -0.02  -0.01     0.00   0.01   0.00     0.00  -0.01  -0.03
    4  14     0.00  -0.01  -0.01    -0.00  -0.02   0.00     0.00   0.00  -0.03
    5   8     0.01  -0.01  -0.01     0.02   0.03  -0.01     0.00  -0.02  -0.02
    6  14    -0.01  -0.00   0.00     0.00  -0.01   0.01    -0.02  -0.00  -0.03
    7   8    -0.00   0.01  -0.00     0.02  -0.01   0.00    -0.03  -0.02  -0.02
    8   8    -0.00   0.00   0.00    -0.01  -0.01  -0.00    -0.02  -0.01  -0.03
    9  14    -0.03   0.01  -0.01     0.01   0.01  -0.01     0.03  -0.03   0.01
   10   8    -0.05   0.02  -0.01     0.00   0.01  -0.02     0.06  -0.06   0.01
   11   8    -0.03  -0.02  -0.03     0.01  -0.00  -0.01     0.03   0.01   0.05
   12  14    -0.01   0.01   0.00     0.01   0.00  -0.02    -0.01  -0.03  -0.01
   13   8    -0.01  -0.01   0.01     0.01  -0.01  -0.01    -0.01   0.00  -0.04
   14  14    -0.01   0.01  -0.02     0.01  -0.02  -0.01    -0.01   0.01  -0.03
   15   8    -0.04   0.03  -0.03     0.02  -0.03  -0.01    -0.00   0.01  -0.03
   16   8     0.01   0.01  -0.03    -0.00  -0.02   0.00     0.00   0.01  -0.04
```

17	14	0.01	-0.01	-0.01	0.00	0.01	0.01	-0.00	-0.00	-0.02
18	8	0.01	-0.00	-0.02	-0.03	-0.02	0.02	-0.01	-0.00	-0.04
19	14	0.02	0.01	-0.03	-0.01	-0.01	-0.01	-0.01	-0.01	-0.03
20	8	0.02	0.00	-0.03	0.01	-0.01	-0.01	-0.01	-0.00	-0.03
21	14	-0.00	0.00	0.01	-0.01	-0.02	-0.00	-0.01	0.00	-0.04
22	6	-0.00	0.01	0.02	-0.01	-0.02	-0.01	0.02	-0.00	-0.04
23	6	-0.01	0.02	0.02	0.00	-0.03	-0.01	-0.00	0.01	-0.04
24	6	-0.01	0.01	0.03	0.01	-0.02	-0.02	-0.01	0.00	-0.02
25	1	-0.01	0.02	0.02	0.01	-0.03	-0.01	-0.01	0.02	-0.04
26	1	-0.00	0.02	0.01	-0.00	-0.03	-0.01	0.00	0.01	-0.04
27	15	-0.01	0.00	0.02	0.00	-0.00	-0.02	0.01	-0.01	0.01
28	1	-0.01	0.02	0.03	0.02	-0.02	-0.02	-0.03	-0.00	-0.02
29	1	-0.01	0.01	0.04	0.02	-0.02	-0.02	-0.01	0.00	-0.02
30	8	-0.01	-0.00	0.02	0.01	-0.00	-0.04	-0.02	0.01	0.06
31	8	0.00	0.00	0.02	-0.01	-0.00	-0.01	0.01	-0.01	-0.04
32	8	-0.01	-0.01	0.03	-0.01	0.01	-0.03	0.06	-0.05	0.03
33	8	0.00	0.02	-0.02	-0.02	-0.00	-0.02	-0.00	-0.01	-0.02
34	8	-0.00	0.01	0.00	-0.01	-0.02	0.00	0.01	0.00	-0.04
35	14	-0.00	0.02	-0.00	-0.01	-0.01	-0.01	0.01	-0.02	-0.04
36	8	-0.01	-0.01	0.01	-0.01	-0.00	0.02	-0.02	0.01	-0.05
37	14	0.01	-0.02	0.01	-0.00	-0.00	0.02	-0.06	0.05	-0.03
38	14	-0.03	-0.02	-0.03	0.00	0.00	0.01	0.02	0.02	0.10
39	8	0.01	-0.01	0.01	-0.00	-0.00	0.02	-0.04	0.03	-0.05
40	8	-0.02	-0.02	-0.01	0.00	0.00	0.02	0.03	0.01	0.05
41	14	-0.01	-0.02	-0.01	-0.00	0.01	0.02	0.04	0.01	0.04
42	14	0.00	-0.00	0.01	-0.01	0.01	0.03	0.00	-0.01	-0.06
43	8	-0.00	-0.01	0.01	-0.01	0.00	0.02	0.03	-0.01	-0.02
44	8	0.03	0.01	-0.03	-0.02	-0.00	-0.02	-0.01	-0.01	-0.03
45	14	0.04	0.01	-0.02	-0.02	0.01	-0.02	-0.00	-0.02	-0.02
46	6	0.04	0.01	-0.00	-0.01	0.03	-0.02	-0.01	-0.05	-0.00
47	1	0.04	-0.01	0.00	-0.01	0.04	-0.02	0.00	-0.05	-0.00
48	1	0.03	0.02	0.01	-0.00	0.03	-0.02	-0.03	-0.06	-0.01
49	1	0.04	0.01	-0.01	-0.02	0.03	-0.01	0.00	-0.06	0.01
50	6	0.05	0.01	-0.03	0.00	-0.00	-0.02	-0.02	-0.00	-0.03
51	1	0.06	0.02	-0.03	0.01	0.00	-0.02	-0.01	-0.01	-0.03
52	1	0.05	0.02	-0.04	0.00	-0.02	-0.02	-0.01	0.02	-0.04
53	1	0.04	0.01	-0.03	0.01	-0.00	-0.02	-0.04	-0.01	-0.03
54	6	0.05	0.01	-0.02	-0.04	0.00	-0.01	0.03	-0.01	-0.03
55	1	0.04	0.01	-0.01	-0.04	0.01	-0.02	0.04	-0.02	-0.02
56	1	0.05	0.01	-0.04	-0.04	-0.01	-0.01	0.04	0.01	-0.03
57	1	0.07	0.02	-0.02	-0.04	0.00	-0.01	0.04	-0.02	-0.02
58	14	-0.01	0.09	0.09	0.05	-0.03	-0.00	0.02	0.03	0.01
59	6	0.07	-0.02	0.17	0.06	-0.04	0.00	0.01	-0.00	0.04
60	1	0.12	0.01	0.24	0.08	-0.04	0.01	0.03	0.01	0.07
61	1	0.07	-0.02	0.18	0.05	-0.03	-0.00	-0.01	-0.00	0.02
62	1	0.07	-0.10	0.16	0.05	-0.06	0.01	-0.01	-0.03	0.06
63	6	-0.03	0.22	0.15	0.06	-0.00	-0.01	0.04	0.07	-0.00
64	1	-0.07	0.29	0.08	0.05	0.00	-0.02	0.05	0.10	-0.03
65	1	-0.01	0.18	0.17	0.05	0.00	-0.01	0.03	0.04	-0.02
66	1	-0.01	0.27	0.24	0.07	0.00	-0.00	0.04	0.09	0.03

Below is an example of a fragment of a output file containing information about the normal modes. Gaussian calculation with # freq=(Raman, InternalModes)

```
                          ----------------------------
                          ! Normal Mode   361        !
 --------------------------                            --------------------------
 ! Name  Definition              Value          Relative Weight (%)            !
 --------------------------------------------------------------------------------
 ! R48   R(27,30)               -0.026                 2.1                     !
 ! R51   R(30,34)                1.0595               85.1                     !
 ! A85   A(24,27,30)            -0.018                 1.4                     !
 ! D158  D(30,27,32,33)         -0.0181                1.5                     !
 --------------------------------------------------------------------------------
                          ----------------------------
                          ! Normal Mode   362        !
 --------------------------                            --------------------------
 ! Name  Definition              Value          Relative Weight (%)            !
 --------------------------------------------------------------------------------
 ! R63   R(40,109)               0.0278                2.1                     !
 ! R116  R(99,100)               0.0107                0.8                     !
 ! R119  R(105,106)              0.0124                0.9                     !
 ! R120  R(107,108)             -0.0201                1.5                     !
 ! R121  R(109,110)             -1.0591               80.5                     !
 ! R123  R(113,114)              0.0163                1.2                     !
 ! R124  R(115,116)              0.0291                2.2                     !
 ! R125  R(117,118)              0.0182                1.4                     !
 ! A109  A(11,40,109)            0.0092                0.7                     !
 ! A111  A(42,40,109)            0.0093                0.7                     !
 ! A112  A(107,40,109)          -0.0172                1.3                     !
 ! D63   D(9,11,40,109)         -0.0157                1.2                     !
 ! D183  D(109,40,42,43)         0.0157                1.2                     !
 --------------------------------------------------------------------------------
                          ----------------------------
                          ! Normal Mode   363        !
 --------------------------                            --------------------------
 ! Name  Definition              Value          Relative Weight (%)            !
 --------------------------------------------------------------------------------
 ! R71   R(44,105)              -0.0286                1.9                     !
 ! R119  R(105,106)              1.0352               68.6                     !
 ! R123  R(113,114)             -0.0737                4.9                     !
 ! R125  R(117,118)             -0.2157               14.3                     !
 ! A123  A(41,44,105)            0.0131                0.9                     !
 ! A126  A(103,44,105)          -0.0163                1.1                     !
 ! D192  D(39,41,44,105)         0.0121                0.8                     !
 ! D207  D(105,44,45,43)        -0.0181                1.2                     !
 --------------------------------------------------------------------------------
                          ----------------------------
                          ! Normal Mode   364        !
 --------------------------                            --------------------------
 ! Name  Definition              Value          Relative Weight (%)            !
 --------------------------------------------------------------------------------
 ! R60   R(39,117)              -0.0294                1.9                     !
 ! R116  R(99,100)              -0.0117                0.8                     !
 ! R119  R(105,106)              0.2104               13.6                     !
 ! R121  R(109,110)              0.0192                1.2                     !
 ! R123  R(113,114)             -0.0748                4.8                     !
 ! R125  R(117,118)              1.0359               67.1                     !
 ! A105  A(41,39,117)           -0.0168                1.1                     !
 ! A106  A(115,39,117)           0.0107                0.7                     !
```

```
! D171  D(6,38,39,117)          0.0171                  1.1                    !
! D177  D(117,39,115,116)      -0.0138                  0.9                    !
--------------------------------------------------------------------------------
```

3 Step three - Analysis of the movements ot the atoms

Next, we analyze the motions of the atoms. This is a large version of Figure 7b from the publication.

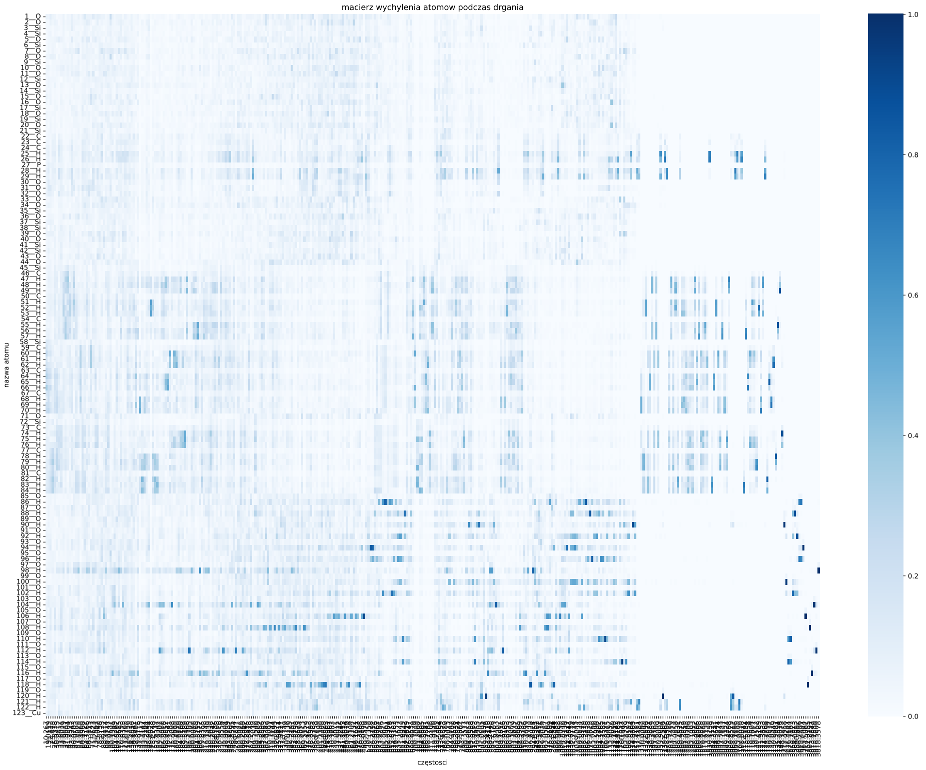

Figure S3: The atom displacement matrix for individual vibrational modes (Large version of Figure 7b from publication.)

4 Step four - Grouping of atoms

We group atoms, for example, by clustering. For each group of atoms, we obtain a fragment of the spectrum. It corresponds to Figure 7c in the publication.

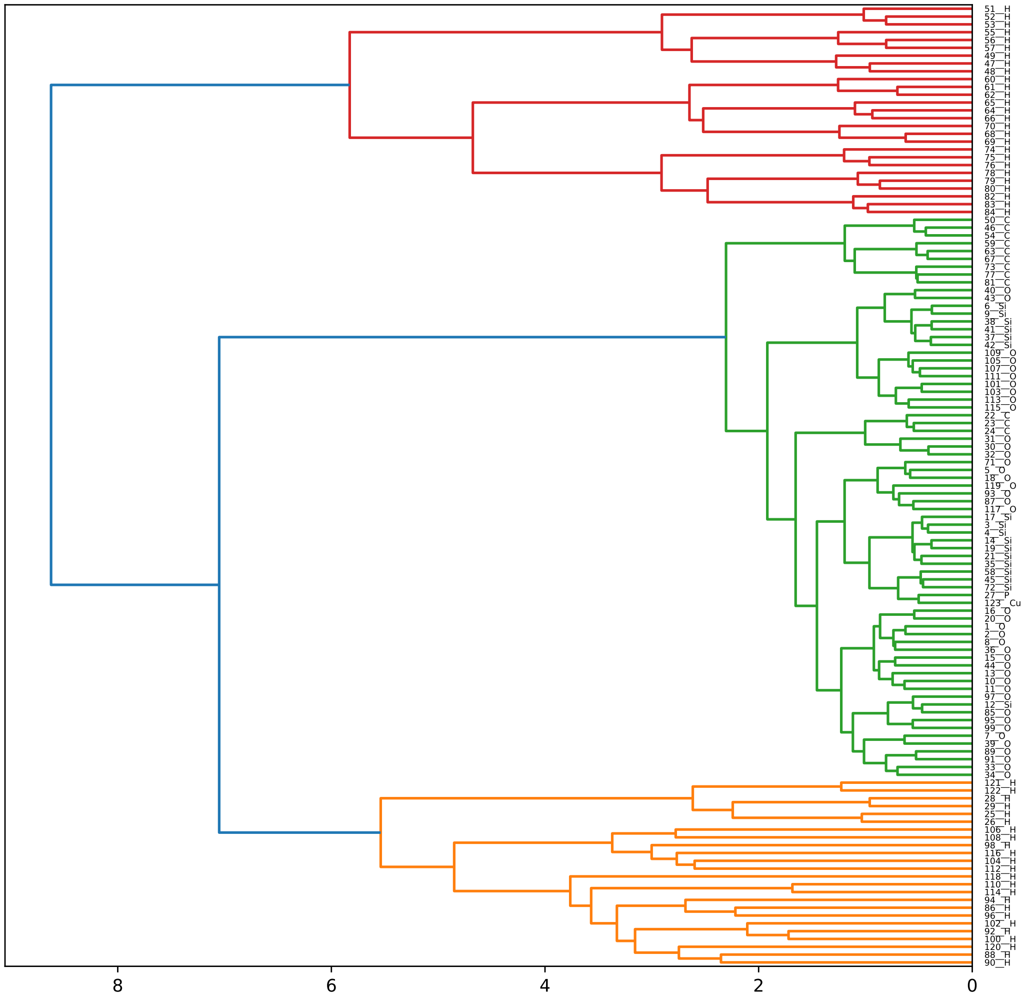

Figure S4: Gruping atoms by clustering

5 Step five - Visualization data

We visualize it using a stack plot or a cumulative layer chart.

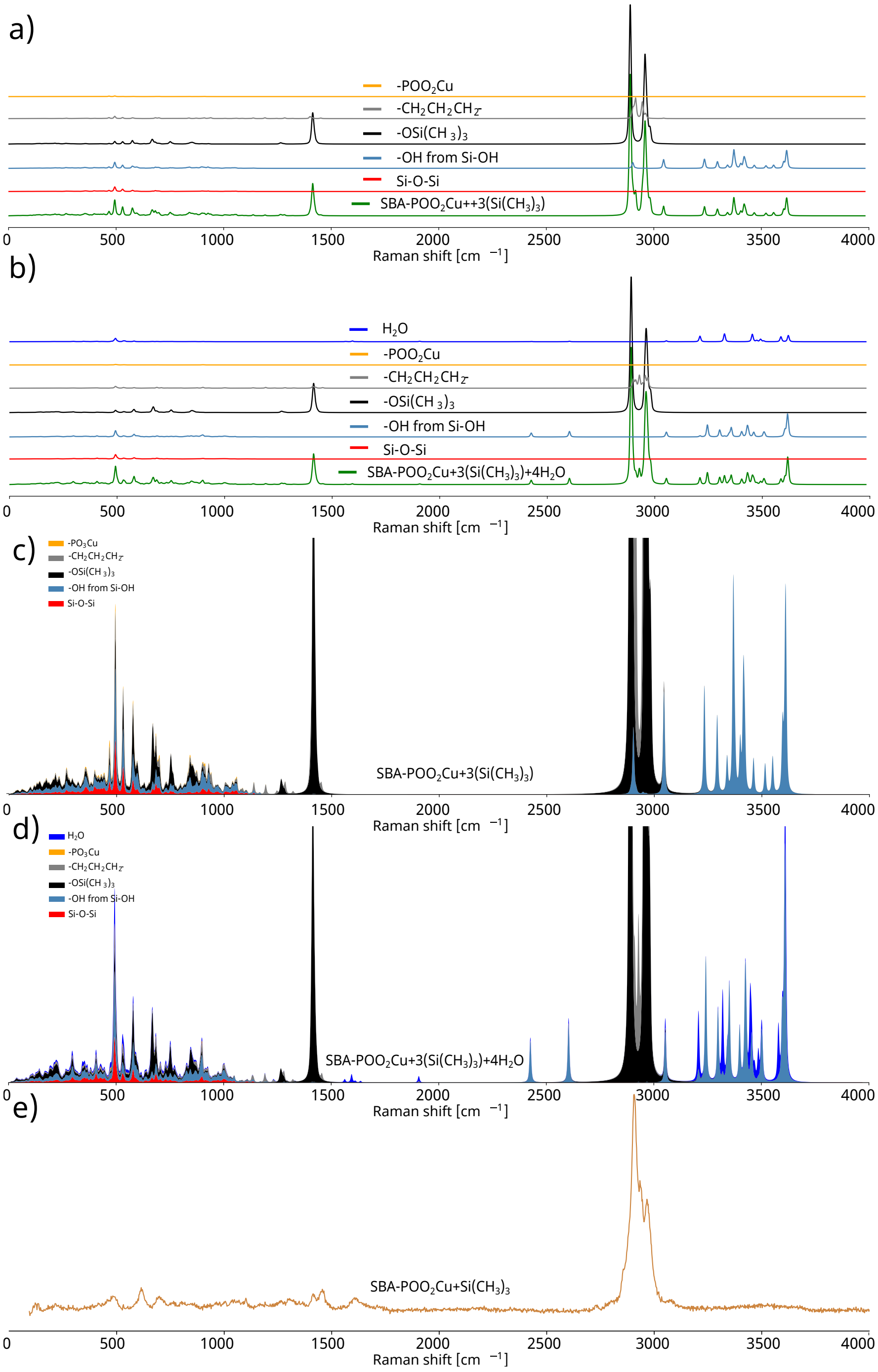

Figure S5: Theoretical spectra of SBA-POO$_2$Cu+3(Si(CH$_3$)$_3$) (a) and SBA-POO$_2$Cu+3(Si(CH$_3$)$_3$)+4H$_2$O (b) split into specific fragments, cumulative stacked area chart of SBA-POO$_2$Cu+3(Si(CH$_3$)$_3$) (c) and SBA-POO$_2$Cu+3(Si(CH$_3$)$_3$)+4H$_2$O (d) theoretical spectra, experimental spectrum of SBA-POO$_2$Cu+Si(CH$_3$)$_3$ (e).

6 Example as a Jupyter-Lab notebook

Example of a notebook in which we conducted a working analysis. If you want to repeat the analysis, please cite it. If you want to try out the method, but you lack experience, write to us, we will be happy to help.

[1]:
```
pwd
```

[1]: '/home/MarekD/RAMAN/2025_RAMAN_IR_Biocinal'

[2]:
```
PLIK_DO_ANALIZY =␣
  ↪"gv5_SBA15_Dh2o_D4_173_50_FREQ_2_Cu_4H2O_FREQ_17_FREQ.log"␣
  ↪#
KATALOG = "2025_RAMAN_DEC_SBA_P002_Cu_4H2O"
```

[3]:
```
#
import numpy as np
from matplotlib import pyplot as plt
import rampy
from sklearn import preprocessing
import time
import scipy # For data manipulation
import random
import pandas as pd
import matplotlib.pyplot as plt # For doing the plots
from math import sqrt
import matplotlib.pyplot as plt
import seaborn as sns
import os
import py3Dmol
from scipy.special import wofz
from scipy.cluster.hierarchy import dendrogram, linkage

!mkdir $KATALOG
!cp $PLIK_DO_ANALIZY $KATALOG/$PLIK_DO_ANALIZY
!cd $KATALOG
os.chdir(KATALOG)
```

```
!pwd
NAZWAPLIKU = PLIK_DO_ANALIZY #␣
 ↪'gv5_SBA15_Dh2o_D4_173_50_FREQ_2.log' # 'A01.log'
```

```
,,2025_RAMAN_DEC_SBA_P002_Cu_4H2O'': Plik
/home/MarekD/RAMAN/2025_RAMAN_IR_Biocinal/
 ↪2025_RAMAN_DEC_SBA_P002_Cu_4H2O
```

[4]:
```
#

def generowanie_plikow_z_obliczen_freq(NAZWAPLIKU):
    FILENAMElog = NAZWAPLIKU
    #FILENAMElog = 'A01.log'
    FILENAME = FILENAMElog.replace(".log", "")
    print(FILENAME)

    #
    plik_odczyt = open(FILENAMElog, 'rt')
    #linia = plik_odczyt.readline()
    #print(f"Pierwsza linia: {linia}", end = '')
    #print("Następne linie:")
    #linie = []
    l = 1
    ii = 0
    naglowek = 0
    naglowekKONIEC = 0
    naglowekZMATRIX = 0
    NAtoms = []
    pierwszy=0  #

    #
    #
    while True:
        linia = plik_odczyt.readline()
        #  linii:
        if not linia:
```

```
            break

        else:
            if (linia.find('and normal coordinates:') != -1): ␣
↪ #
                print("LINIA PLIKU - start FREQ PART", l,":")
                print(linia)
                naglowek = l
                ii += 1
            else:
                #print(" ")
                a = 0

            if (linia.find('Thermochemistry') != -1):    #
                print("LINIA PLIKU - koniec FREQ PART", l,":")
                print(linia)
                naglowekKONIEC = l
                ii += 1

            if (linia.find('Symbolic Z-matrix:') != -1):    #
                print("LINIA PLIKU Z-Matrix", l,":")
                print(linia)
                naglowekZMATRIX = l
                ii += 1

            if (linia.find('NAtom') != -1):    #
                if pierwszy ==0:
                    #print(" LINE:", l,":")
                    print(linia)
                    robimytablice = linia.split()
                    print(robimytablice[1], "<-  ")
                    NAtoms = int(robimytablice[1])
                    #print(f"Linia {l}: {linia}", end = '')
                    ii += 1
                    pierwszy=1
```

```
        else:
            #print("")
            a = 0


        if ii == 1000:
            break
    l += 1
print("L NAtoms", NAtoms)
plik_odczyt.close()





######### 2
#STEP  Z-MATRIX

plik_odczyt = open(FILENAMElog, "r")
linie = plik_odczyt.readlines()[:]

NAGLOEWEKZMATRIX = pd.DataFrame()
LPall = []

#
#

nazwaatomu = []

for iii in range(0,NAtoms):
    #print(linie[naglowekZMATRIX+1+iii:
↪naglowekZMATRIX+2+iii])
    #print("JESTEM NA ETAPIE:", iii)
    LPall = []
    LP = linie[naglowekZMATRIX+1+iii:naglowekZMATRIX+2+iii]
```

```
        LPall += (LP[0].split())
        #print(LP)
        NAGLOEWEKZMATRIX[iii] = pd.DataFrame(LPall)
        #print(iii, "AAAAAAA")
        #NAGLOEWEK.to_csv("cc.csv")#, index=False)
        test = str(iii+1)+"__"+LPall[0]
        nazwaatomu.append(test)
        #print(nazwaatomu[iii])

    NAGLOEWEKZMATRIX =NAGLOEWEKZMATRIX.transpose()
    NAGLOEWEKZMATRIX.columns = ["Atom", "x", "y", "z"]
    print(NAGLOEWEKZMATRIX)
    #NAGLOEWEKZMATRIX.to_txt(FILENAME+"XYZ.xyz", index=False)

    plik_odczyt.close()

    with open(FILENAME+"_XYZ.xyz", 'w') as f:
        df_string = NAGLOEWEKZMATRIX.to_string(header=False,␣
↪index=False)
        f.write(df_string)

    # For  XANES
    # X,Y,Z


    #########ETAP 3
    #

    NAGLOEWEKZMATRIX['x'] = pd.
↪to_numeric(NAGLOEWEKZMATRIX['x'], errors='coerce')
    NAGLOEWEKZMATRIX['y'] = pd.
↪to_numeric(NAGLOEWEKZMATRIX['y'], errors='coerce')
    NAGLOEWEKZMATRIX['z'] = pd.
↪to_numeric(NAGLOEWEKZMATRIX['z'], errors='coerce')
```

```
    # x, y, z
    xmin = NAGLOEWEKZMATRIX['x'].min()
    ymin = NAGLOEWEKZMATRIX['y'].min()
    zmin = NAGLOEWEKZMATRIX['z'].min()

    #

    NAGLOEWEKZMATRIX['x'] = NAGLOEWEKZMATRIX['x']-xmin
    NAGLOEWEKZMATRIX['y'] = NAGLOEWEKZMATRIX['y']-ymin
    NAGLOEWEKZMATRIX['z'] = NAGLOEWEKZMATRIX['z']-zmin

    #
    xmax = NAGLOEWEKZMATRIX['x'].max()
    ymax = NAGLOEWEKZMATRIX['y'].max()
    zmax = NAGLOEWEKZMATRIX['z'].max()

    df_string = str(NAtoms)
    df_string += "\n\n"

    with open(FILENAME+"_XYZcenter.xyz", 'w') as f:  # 'a' is␣
↪appeding , w is write
        df_string += NAGLOEWEKZMATRIX.to_string(header=False,␣
↪index=False)
        f.write(df_string)
    #




    FILENAMElog = NAZWAPLIKU
    FILENAME = FILENAMElog.replace(".log", "")
    print(FILENAME)




    plik_odczyt = open(FILENAMElog, "r")
```

```
linie = plik_odczyt.readlines()[:]
#linie =open(FILENAME, "r").readlines()[:]

NAGLOEWEK = pd.DataFrame()
#LPall = []
#LPallpd = pd.DataFrame()
for iii in range(0,7):
    #print(":", iii)
    LPall = []
    #LPall = pd.DataFrame()
    drukujOD = naglowek+1 +iii +1
    drukujDO = naglowek+2 +iii +1
    ii = 1
    i=1
    while drukujDO < naglowekKONIEC-3:
        #print(ii)
        #print(linie[drukujOD:drukujDO])
        LP = linie[drukujOD:drukujDO]
        LP[0] = LP[0].replace("--", "")
        LP[0] = LP[0].replace("Frequencies", "")
        LP[0] = LP[0].replace("Red. masses", "")
        LP[0] = LP[0].replace("Frc consts", "")
        LP[0] = LP[0].replace("IR Inten", "")
        LP[0] = LP[0].replace("Raman Activ", "")
        LP[0] = LP[0].replace("Depolar (P)", "")
        LP[0] = LP[0].replace("Depolar (U)", "")
        LP[0] = LP[0].replace('\n', "")
        LPall += (LP[0].split())
        #print(LP)
        drukujOD += (2+8+NAtoms)
        drukujDO += (2+8+NAtoms)
        ii += 3
        i += 1
    #print(LPall,"------")
    NAGLOEWEK[iii] = pd.DataFrame(LPall)
```

```
        #print(iii, "AAAAAAA")
        #NAGLOEWEK.to_csv("cc.csv")#, index=False)
    #print(NAGLOEWEK)
    NAGLOEWEK.columns = ["Frequencies","Red_masses",␣
↪"Frc_consts", "IR_Inten", "Raman_Activ", "Depolar_P",␣
↪"Depolar_U"]
    NAGLOEWEK.to_csv(FILENAME+"_FREQ.csv")#, index=False)

    plik_odczyt.close()




    plik_odczyt = open(FILENAMElog, "r")
    linie = plik_odczyt.readlines()[:]

    #
    naglowekindex = naglowek
    NAGLOEWEK = pd.DataFrame()
    NAGLOEWEKL = pd.DataFrame()  #
    NAGLOEWEKiii = 0
    while naglowekindex < naglowekKONIEC:
        #print(linie[naglowekindex])
    #
        if (linie[naglowekindex].find('Atom') != -1):
            liczbaX=0
            #print("Line: Atom")
            # #  X (1,2,3)
            for i in linie[naglowekindex].split():
                if (i == "X"):
                    liczbaX +=1
     #             print(liczbaX, "a")
            #
            X1 = []
            Y1 = []
```

```
            Z1 = []
            L1 = []
            X2 = []
            Y2 = []
            Z2 = []
            L2 = []
            X3 = []
            Y3 = []
            Z3 = []
            L3 = []
            for i in range (naglowekindex+1,␣
↪naglowekindex+NAtoms+1):
                temp = (linie[i].split())
                X1.append(temp[2])
                Y1.append(temp[3])
                Z1.append(temp[4])
                L1.append(sqrt(float(temp[2])**2 +␣
↪float(temp[3])**2 + float(temp[4])**2 ) )
            NAGLOEWEK = pd.concat([NAGLOEWEK, pd.DataFrame(X1).
↪T, pd.DataFrame(Y1).T, pd.DataFrame(Z1).T  ])
            NAGLOEWEKL = pd.concat([NAGLOEWEKL, pd.
↪DataFrame(L1).T ])
            if (liczbaX >1):
                for i in range (naglowekindex+1,␣
↪naglowekindex+NAtoms+1):
                    temp = (linie[i].split())
                    X2.append(temp[2+3])
                    Y2.append(temp[3+3])
                    Z2.append(temp[4+3])
                    L2.append(sqrt(float(temp[2+3])**2 +␣
↪float(temp[3+3])**2 + float(temp[4+3])**2 ) )
                NAGLOEWEK = pd.concat([NAGLOEWEK, pd.
↪DataFrame(X2).T, pd.DataFrame(Y2).T, pd.DataFrame(Z2).T  ])
                NAGLOEWEKL = pd.concat([NAGLOEWEKL, pd.
↪DataFrame(L2).T ])
```

```
          if (liczbaX >2):
              for i in range (naglowekindex+1, naglowekindex+NAtoms+1):
                  temp = (linie[i].split())
                  X3.append(temp[2+6])
                  Y3.append(temp[3+6])
                  Z3.append(temp[4+6])
                  L3.append(sqrt(float(temp[2+6])**2 + float(temp[3+6])**2 + float(temp[4+6])**2 ) )
              NAGLOEWEK = pd.concat([NAGLOEWEK, pd.DataFrame(X3).T, pd.DataFrame(Y3).T, pd.DataFrame(Z3).T  ])
              NAGLOEWEKL = pd.concat([NAGLOEWEKL, pd.DataFrame(L3).T ])

      naglowekindex +=1


  NAGLOEWEKL.to_csv(FILENAME+"_FREQ_Atom_Long.csv") #, index=False)
  NAGLOEWEK.to_csv(FILENAME+"_FREQ_Atom.csv") #, index=False)
  NAGLOEWEK.columns = NAGLOEWEKZMATRIX.iloc[:,0]
  NAGLOEWEK.to_csv(FILENAME+"_FREQ_Atom_colname.csv") #, index=False)

  NAGLOEWEKL.columns = nazwaatomu #NAGLOEWEKZMATRIX.iloc[:,0]
  NAGLOEWEKL.to_csv(FILENAME+"_FREQ_Atom_Long_colname.csv") #, index=False)

  NAGLOEWEKZMATRIX.iloc[:,0].to_csv(FILENAME+"_Atom_Name.csv") #, index=False)
  print(nazwaatomu) #.iloc[:,:])
  plik_odczyt.close()
```

```python
def Gauss(x, mu, sigma, A = 1):
    # This def returns the Gaussian function of x
    # x is an array
    # mu is the expected value
    # sigma is the square root of the variance
    # A is a multiplication factor
    gaussian = A/(sigma * np.sqrt(2*np.pi)) * np.exp(-0.5*((x-mu)/sigma)**2)
    return gaussian
def G(X, alpha):
    return np.sqrt(np.log(2) / np.pi)/ alpha* np.exp(-(x/alpha)**2 *np.log(2))
def L(x,gamma):
    return gamma / np.pi / (x**2 + gamma**2)
def V(x, alpha, gamma):
    sigma = alpha / np.sqrt(2*np.log(2))
    return np.real(wofz((x+1j*gamma)/sigma/np.sqrt(2)))/sigma/np.sqrt(2*np.pi)

def visualization_shape():
    alpha, gamma = 0.1, 0.1
    x = np.linspace(-500, 500,12000) # start stop number
    alpha = 0.21
    plt.plot(x, G(x,alpha), ls=':', label='Gaussian: ' + 'alpha =' + str(alpha) )
    gamma = 0.14
    plt.plot(x, L(x,gamma+0.04), ls='--', label='Lorentzian: ' + 'gamma =' + str(gamma))
    alpha, gamma = 1.2, 1.2
    plt.plot(x, V(x,alpha, gamma), ls='-', label='Voigt: ' + 'alpha =' + str(alpha) +  ' gamma =' + str(gamma))
    plt.hlines(0, xmin=0, xmax=10, color='b', lw=-.1)
    plt.xlim(-20,20)
    plt.legend()
```

```
    xtemp = np.linspace(-8200, 8200,(8200)*2*10) #
    alpha = 3.1 #10 #3.1
    gamma = 3.1 #10 #3.1
    ytemp = V(xtemp,alpha=alpha, gamma=gamma)

    x = np.linspace(0, 4200,42000)
    #
    #
    plt.show()
    plt.plot(xtemp, ytemp, ls='-', label='Voigt')
    plt.hlines(0, xmin=-1000, xmax=1000, color='b', lw=1)
    plt.xlim(-50,50)
```

[5]:

```


NAZWAPLIKU = PLIK_DO_ANALIZY #␣
 ↪'gv5_SBA15_Dh2o_D4_173_50_FREQ_2.log' # 'A01.log'

#Part 1 -
generowanie_plikow_z_obliczen_freq(NAZWAPLIKU)



FILENAMElog = NAZWAPLIKU
FILENAME = FILENAMElog.replace(".log", "")
print(FILENAME)

view(width=450, height=450)
#view.addModel(open('5RH2.pdb', 'r').read(),'pdb')
#view.addModel(open("../RAMANG/SPECTRA_FREQ/propoane.xyz",␣
 ↪'r').read(), "xyz")
view.setStyle({'sphere':{}}, viewer=(0,2))
#view.addModel(open("../RAMANG/SPECTRA_FREQ/
 ↪SBA_15_Cu_model_XYZ.xyz", 'r').read(), "xyz")
view.addModel(open(FILENAME+"_XYZcenter.xyz", 'r').read(),␣
 ↪"xyz")
```

```
#view.setBackgroundColor('white')
#view.setStyle({'chain':'A'}, {'cartoon': {'color':'purple'}})
#view.addStyle({'resn':'UH7'}, {'stick': {'colorscheme':
↪'yellowCarbon'}})
#view.addStyle({'within':{'distance':'5', 'sel':{'resn':
↪'UH7'}}}, {'stick': {}})
view.setStyle({'stick':{'colorscheme':'default'}},␣
↪viewer=(0,1))
#view.setStyle({'sphere':{}}, viewer=(0,2))

licznik =0
with open(FILENAME+"_XYZcenter.xyz", 'r') as plik:
    linie = plik.readlines()  #
    for linia in linie[2:]:  #
        licznik += 1
        podzielone = linia.strip().split()
        #print(str(licznik) + "  " +podzielone[0])
        #print(str(licznik)+ "__"+linia.strip())
        view.addLabel(str(licznik) +" "+podzielone[0],{'font':
↪'sans-serif','fontSize':12,'fontColor':
↪'black','fontOpacity':1,'borderThickness':0.0,'borderColor':
↪'red','borderOpacity':0.0,'backgroundColor':
↪'black','backgroundOpacity':0.0,'position':{'x':
↪float(podzielone[1]),'y':float(podzielone[2]),'z':
↪float(podzielone[3])},'inFront':'true','showBackground':
↪'true'});
        #print(licznik)


view.zoomTo()
view.render()
```

```python
#view.png()

view.animate({'loop': "forward"})
view.show()
#view.png()
view.savefig(FILENAME+'_czasteczka.png')
#view.savefig('czasteczka.png')
view.write_html(FILENAME+'_czasteczka.html')
```

```
gv5_SBA15_Dh2o_D4_173_50_FREQ_2_Cu_4H2O_FREQ_17_FREQ
LINIA PLIKU Z-Matrix 105 :
 Symbolic Z-matrix:

 NAtoms=     135 NQM=       135 NQMF=       0 NMMI=       0 NMMIF=
 ↪      0

135 <-  TO jest nasza liczba atomów
LINIA PLIKU - start FREQ PART 2450 :
 and normal coordinates:

LINIA PLIKU - koniec FREQ PART 21738 :
 - Thermochemistry -

LINIA PLIKU NAtoms 135
    Atom        x         y         z
0      O  0.81109  -1.74794  -2.08954
1      O  2.86318  -0.16764  -2.69549
2     Si  3.77923  -0.60775  -1.41403
3     Si  1.21326  -0.28448  -2.70797
4      O  3.20732   0.19127  -0.09077
..   ...      ...       ...       ...
130    H  7.02001  -5.90678   0.75107
131    H  6.39104  -5.49274  -0.61596
132    H  7.14573  -2.95823  -0.23812
133    H   4.8297  -2.17864   2.33463
134    H   4.6551  -3.87829  -1.95417
```

```
[135 rows x 4 columns]
gv5_SBA15_Dh2o_D4_173_50_FREQ_2_Cu_4H2O_FREQ_17_FREQ
['1__O', '2__O', '3__Si', '4__Si', '5__O', '6__Si', '7__O',␣
 ↪'8__O', '9__Si',
'10__O', '11__O', '12__Si', '13__O', '14__Si', '15__O',␣
 ↪'16__O', '17__Si',
'18__O', '19__Si', '20__O', '21__Si', '22__C', '23__C',␣
 ↪'24__C', '25__H',
'26__H', '27__P', '28__H', '29__H', '30__O', '31__O', '32__O',␣
 ↪'33__O', '34__O',
'35__Si', '36__O', '37__Si', '38__Si', '39__O', '40__O',␣
 ↪'41__Si', '42__Si',
'43__O', '44__O', '45__Si', '46__C', '47__H', '48__H', '49__H',␣
 ↪'50__C',
'51__H', '52__H', '53__H', '54__C', '55__H', '56__H', '57__H',␣
 ↪'58__Si',
'59__C', '60__H', '61__H', '62__H', '63__C', '64__H', '65__H',␣
 ↪'66__H', '67__C',
'68__H', '69__H', '70__H', '71__O', '72__Si', '73__C', '74__H',␣
 ↪'75__H',
'76__H', '77__C', '78__H', '79__H', '80__H', '81__C', '82__H',␣
 ↪'83__H', '84__H',
'85__O', '86__H', '87__O', '88__H', '89__O', '90__H', '91__O',␣
 ↪'92__H', '93__O',
'94__H', '95__O', '96__H', '97__O', '98__H', '99__O', '100__H',␣
 ↪'101__O',
'102__H', '103__O', '104__H', '105__O', '106__H', '107__O',␣
 ↪'108__H', '109__O',
'110__H', '111__O', '112__H', '113__O', '114__H', '115__O',␣
 ↪'116__H', '117__O',
'118__H', '119__O', '120__H', '121__H', '122__H', '123__Cu',␣
 ↪'124__O', '125__H',
'126__O', '127__H', '128__O', '129__H', '130__O', '131__H',␣
 ↪'132__H', '133__H',
```

```
'134__H', '135__H']
gv5_SBA15_Dh2o_D4_173_50_FREQ_2_Cu_4H2O_FREQ_17_FREQ
```

[6]:

```
view = py3Dmol.view(width=350, height=350)

#view.addModel(open("../RAMANG/SPECTRA_FREQ/propoane.xyz",␣
↪'r').read(), "xyz")
view.setStyle({'sphere':{}}, viewer=(0,2))
#view.addModel(open("../RAMANG/SPECTRA_FREQ/
↪SBA_15_Cu_model_XYZ.xyz", 'r').read(), "xyz")
view.addModel(open(FILENAME+"_XYZcenter.xyz", 'r').read(),␣
↪"xyz")

view.zoomTo()
view.render()
#view.png()
xtemp = np.linspace(-8200, 8200,(8200)*2*10) #
alpha = 3.1# 2.1# 10 #3.1
gamma = 3.1 #2.1#10 #3.1
ytemp = V(xtemp,alpha=alpha, gamma=gamma)
#ytemp = L(xtemp,gamma=0.14)
x = np.linspace(0, 4200,42000)
#
#
df3 =  pd.read_csv(FILENAME+'_FREQ_Atom_Long_colname.csv').T  ␣
↪                 #  row: atomy, Col   x,y,z, jako wektor
Y_decomposition_new = pd.DataFrame() #
plt.figure(figsize=(20,26))
df1 = pd.read_csv(FILENAME+'_FREQ.csv')
XX = df1.iloc[:,1] # freq
YY = df1.iloc[:,5] # int ramana [:,5] #df1.iloc[:,4] # int. IR␣
↪             ############# RAMAN - 5, IR -4
```

```
for iz in range (0,df3.shape[0]):      #
    Y = x*0 #  Y
    for i in range(0,XX.shape[0],1) :   #
        freq  = XX[i]
        intensity = YY[i]
        #print(str(freq) + "   " + str(intensity))
        if (freq > 0):   #
            #plt.vlines(freq, ymin=0.0, ymax=intensity,␣
 ↪color='b')
            y1 = x*0
            y1 = ytemp[int(round(-freq*10 +164000/2)):
 ↪int(round(-freq*10 +164000/2))+len(x)]*intensity*df3.
 ↪iloc[iz,i]
            #y1[int(round(freq*100,0)):
 ↪int(round(freq*100,0)+len(xtemp))] = ytemp*intensity*df3.
 ↪iloc[49,i] #y1[int(round(freq*100,0)):
 ↪int(round(freq*100,0)+len(xtemp))]+2
            #print(df3.iloc[1,i])
            Y = Y + y1
            y1 = y1*10 +200
            #plt.plot(x,y1, lw=0.1)
    #plt.savefig(FILENAME+'SUMA11.pdf')
    if iz == 0:   #
            print(" ")
    else:
            plt.plot(x, Y+.2*iz, ls='-', c="red", lw=0.4 )␣
 ↪#label='SUMA alpha=11, gamma=11')
    #Y_decomposition.loc[len(Y_decomposition.index)] = Y
    #Y_decomposition.loc[iz] = Y
    Y_decomposition_new = pd.concat([Y_decomposition_new, pd.
 ↪DataFrame(Y).T])
Y_decomposition_new.index = df3.index
Y_decomposition_new = Y_decomposition_new.drop('Unnamed: 0',␣
 ↪axis=0)
Y_decomposition_new.columns = x
plt.yticks([])
```

```
plt.title("OX - cm-1, OY  ")
plt.savefig(FILENAME+'_decomposition_RAMAN.pdf')
pd.DataFrame(Y_decomposition_new).to_csv(FILENAME+"_decomposition_RAMAN.
 ↪csv")
# RAMAN
plt.figure(figsize=(20,12))
plt.subplot(2, 1, 1)
#
df1 = pd.read_csv(FILENAME+'_FREQ.csv')
XX = df1.iloc[:,1] # freq
YY = df1.iloc[:,5] #
Y = x*0 #   Y
for i in range(0,XX.shape[0]) :
    freq  = XX[i]
    intensity = YY[i]
    if (freq > 0):
        plt.vlines(freq, ymin=0.0, ymax=intensity, color='b',␣
 ↪lw=0.2)
        y1 = x*0
        y1 = ytemp[int(round(-freq*10 +164000/2)):
 ↪int(round(-freq*10 +164000/2))+len(x)]*intensity
        Y = Y + y1
        y1 = y1*10 +max(YY)
        plt.plot(x,y1, lw=0.2)
plt.title("RAMAN -  ")
plt.subplot(2, 1, 2)
#plt.show()
#plt.figure(figsize=(20,5))
plt.plot(x, Y_decomposition_new.sum(), ls='-', label='Voigt␣
 ↪function: '+'alpha='+str(alpha)+ ', gamma='+ str(gamma),␣
 ↪c="red")
plt.legend()
plt.title("RAMAN - spectrum")
plt.savefig(FILENAME+'_ALL_RAMAN.pdf')
plt.savefig(FILENAME+'_ALL_RAMAN.png')
pd.DataFrame(Y_decomposition_new.sum()).
```

```
↪to_csv(FILENAME+"_ALL_RAMAN.csv")
#
#
alpha = 7.2 #3.1
gamma = 6.9 #3.1
ytemp = V(xtemp,alpha=alpha, gamma=gamma)
df3 =  pd.read_csv(FILENAME+'_FREQ_Atom_Long_colname.csv').T  ␣
 ↪                #
#Y_DECtemp = pd.read_csv(FILENAME+"_ALL_IR.csv", header=0)    ␣
 ↪               #
#print( (Y_DECtemp.iloc[:,0:1].T).shape )
#Y_decomposition = Y_DECtemp.iloc[:,1:2].T*0    #
Y_decomposition_new = pd.DataFrame() #
plt.figure(figsize=(20,26))
df1 = pd.read_csv(FILENAME+'_FREQ.csv')                        ␣
 ↪                #   ROW: freq , Col: intesity of freq, rama,␣
 ↪act, depolar
XX = df1.iloc[:,1] # freq
YY = df1.iloc[:,4] # int Raman [:,5] #df1.iloc[:,4] # Int IR   ␣
 ↪          ############# RAMAN - 5, IR -4     Ramana
maxOY = 0
for iz in range (0,df3.shape[0]):      #
    Y = x*0 #  Y
    for i in range(0,XX.shape[0],1) :   #
        freq  = XX[i]
        intensity = YY[i]
        #print(str(freq) + "   " + str(intensity))
        if (freq > 0):   #
            #plt.vlines(freq, ymin=0.0, ymax=intensity,␣
 ↪color='b')
            y1 = x*0
            y1 = ytemp[int(round(-freq*10 +164000/2)):
 ↪int(round(-freq*10 +164000/2))+len(x)]*intensity*df3.
 ↪iloc[iz,i]
            #y1[int(round(freq*100,0)):
 ↪int(round(freq*100,0)+len(xtemp))] = ytemp*intensity*df3.
```

```
↪iloc[49,i] #y1[int(round(freq*100,0)):
↪int(round(freq*100,0)+len(xtemp))]+2
            #print(df3.iloc[1,i])
            Y = Y + y1
            y1 = y1*10 +200
            #plt.plot(x,y1, lw=0.1)
    #plt.savefig(FILENAME+'SUMA11.pdf')
    if iz == 0:   #
            print(" ")
    else:
            plt.plot(x, Y+.2*iz, ls='-', c="blue", lw=0.4 )␣
↪#label='SUMA alpha=11, gamma=11')
            maxOY = max([maxOY,max(Y+.2*iz)])
    #Y_decomposition.loc[len(Y_decomposition.index)] = Y
    #Y_decomposition.loc[iz] = Y
    Y_decomposition_new = pd.concat([Y_decomposition_new, pd.
↪DataFrame(Y).T])
Y_decomposition_new.index = df3.index
Y_decomposition_new = Y_decomposition_new.drop('Unnamed: 0',␣
↪axis=0)
Y_decomposition_new.columns = x
plt.ylim(maxOY,0)
plt.xlim(4220,-20)
plt.yticks([])
plt.title("OX - cm-1, OY  ")
plt.savefig(FILENAME+'_decomposition_IR.pdf')
pd.DataFrame(Y_decomposition_new).to_csv(FILENAME+"_decomposition_IR.
↪csv")
# IR
plt.show()
plt.figure(figsize=(20,12))
plt.subplot(2, 1, 1)
# Czytamy
 df1 = pd.read_csv(FILENAME+'_FREQ.csv')
XX = df1.iloc[:,1] #
YY = df1.iloc[:,4] #  df1.iloc[:,5], df1.iloc[:,4] #   IR
```

```
Y = x*0 #     Y
for i in range(0,XX.shape[0]) :
    freq  = XX[i]
    intensity = YY[i]
    if (freq > 0):
        plt.vlines(freq, ymin=0.0, ymax=intensity, color='b',␣
 ↪lw=0.2)
        y1 = x*0
        #
        y1 = ytemp[int(round(-freq*10 +164000/2)):
 ↪int(round(-freq*10 +164000/2))+len(x)]*intensity
        Y = Y + y1
        y1 = y1*10 +max(YY)
        plt.plot(x,y1, lw=0.2)
plt.title("IR - czestosci obliczone")
plt.ylim(1400, min(Y) )    #
plt.xlim(max(x), min(x) )
#plt.savefig(FILENAME+'SUMA_details_IR.pdf')
#plt.show()
plt.subplot(2, 1, 2)
#plt.figure(figsize=(20,5))
Y = Y_decomposition_new.sum()
plt.plot(x, Y, ls='-', label='Voigt function:␣
 ↪'+'alpha='+str(alpha)+ ', gamma='+ str(gamma), c="blue")
plt.legend()
plt.title("IR - widmo z sumy czestosci obliczonej")
plt.ylim(max(Y), min(Y) )
plt.xlim(max(x), min(x) )
plt.savefig(FILENAME+'_ALL_IR.pdf')
plt.savefig(FILENAME+'_ALL_IR.png')
pd.DataFrame(Y).to_csv(FILENAME+"_ALL_IR.csv")
#
#Y_decomposition = (pd.DataFrame(Y)).T
```

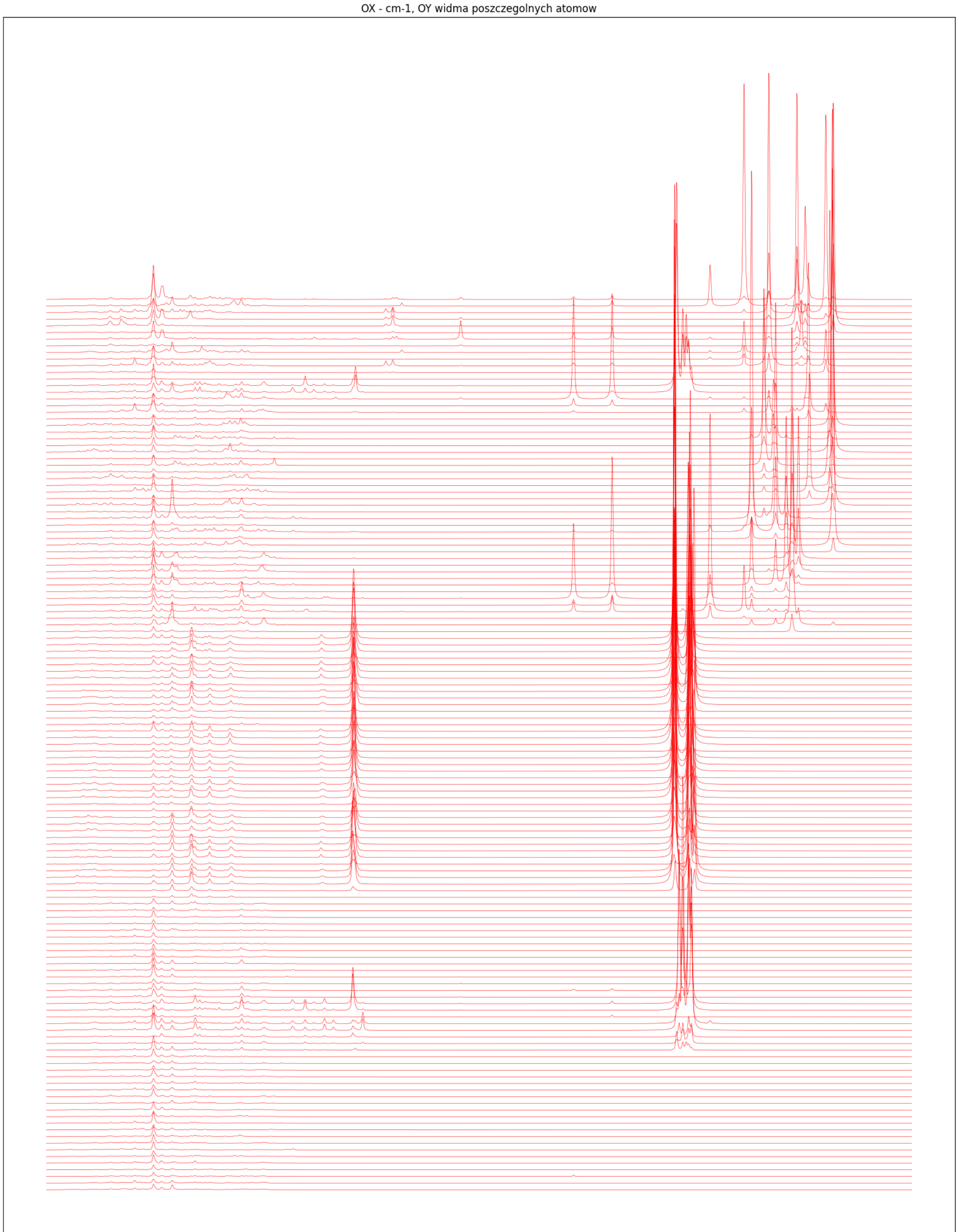
OX - cm-1, OY widma poszczegolnych atomow
0
1000
2000
3000
4000

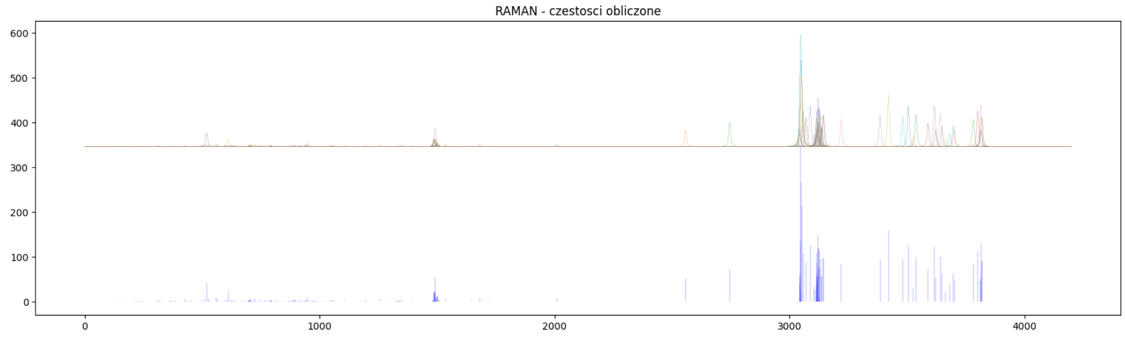
RAMAN - czestosci obliczone
600
500
400
300
200
100
0
0
1000
2000
3000
4000

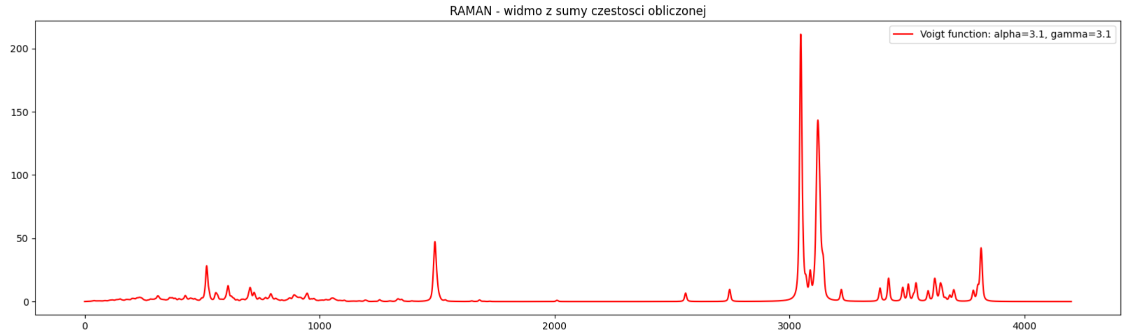
RAMAN - widmo z sumy czestosci obliczonej
Voigt function: alpha=3.1, gamma=3.1
200
150
100
50
0
0
1000
2000
3000
4000

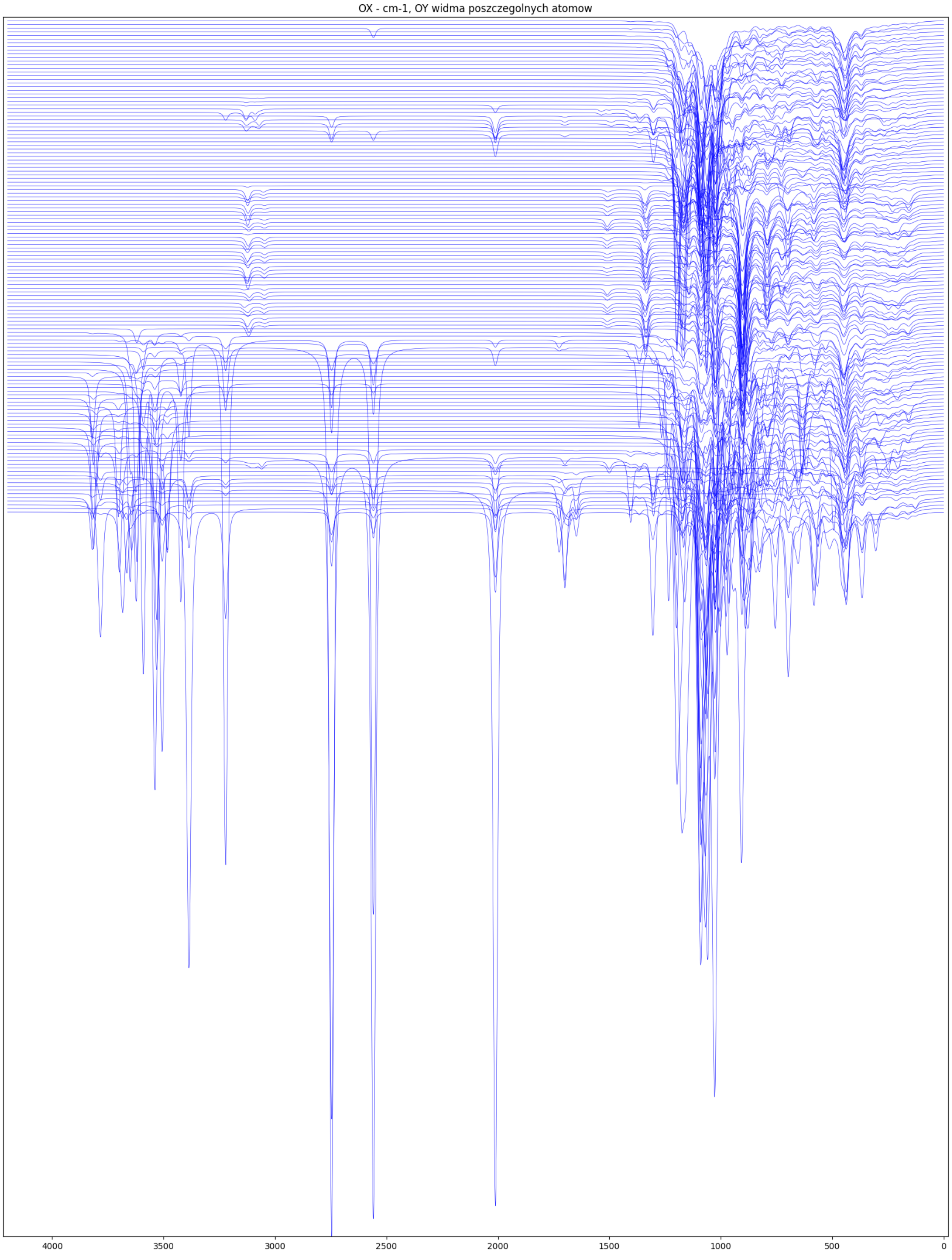
OX - cm-1, OY widma poszczegolnych atomow
4000
3500
3000
2500
2000
1500
1000
500
0

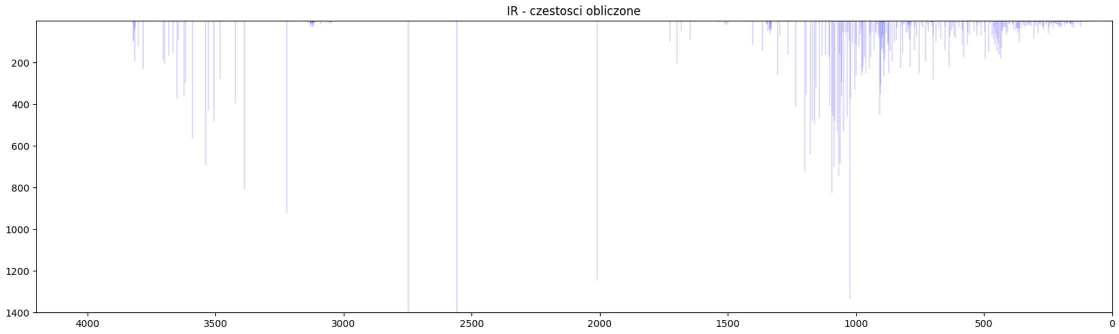

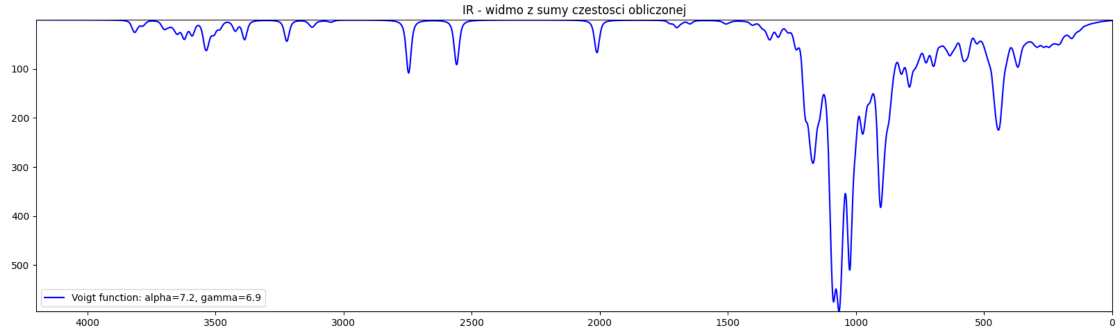

AUTOMATYCZNIE

[7]:
```
#


# Czytamy wychylenie atomow
df2 = pd.read_csv(FILENAME+'_FREQ_Atom_Long_colname.csv').T
df2.iloc[:,:]
df2 = df2.drop(["Unnamed: 0"])  #
df2.columns =df1.iloc[:,1] # nadajemy nazwe kolumn z atomami
plt.imshow(df2.loc[:,:])
plt.title("macierz wychylenia atomow odczas drgania")
plt.xlabel("numer porządkowy częstosci")
plt.ylabel("numer porządkowy atomu")
```

[7]:
```
Text(0, 0.5, 'numer porządkowy atomu')
```

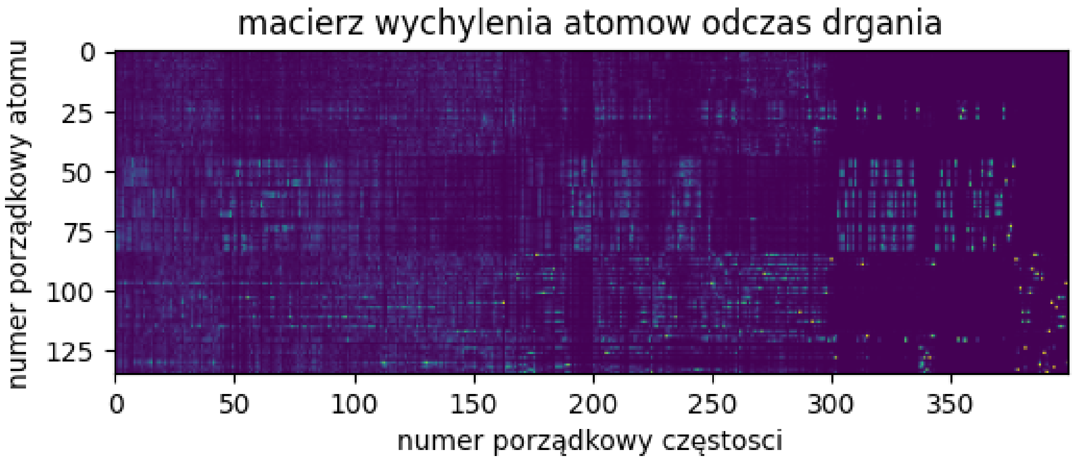

[8]:
```
#
#
from scipy.cluster.hierarchy import dendrogram, linkage
#calculate linkage matrix
linked = linkage(df2, method='ward')
plt.figure(figsize=(10,10))
dendrogram(linked, orientation='left',␣
  ↪distance_sort='descending', show_leaf_counts=True,␣
  ↪labels=df2.index)
```

[8]:
```
{'icoord': [[5.0, 5.0, 15.0, 15.0],
  [25.0, 25.0, 35.0, 35.0],
  [10.0, 10.0, 30.0, 30.0],
  [45.0, 45.0, 55.0, 55.0],
  [50.0, 50.0, 65.0, 65.0],
  [20.0, 20.0, 57.5, 57.5],
  [75.0, 75.0, 85.0, 85.0],
  [80.0, 80.0, 95.0, 95.0],
  [105.0, 105.0, 115.0, 115.0],
  [87.5, 87.5, 110.0, 110.0],
  [38.75, 38.75, 98.75, 98.75],
  [125.0, 125.0, 135.0, 135.0],
```

```
[145.0, 145.0, 155.0, 155.0],
[130.0, 130.0, 150.0, 150.0],
[165.0, 165.0, 175.0, 175.0],
[140.0, 140.0, 170.0, 170.0],
[68.75, 68.75, 155.0, 155.0],
[185.0, 185.0, 195.0, 195.0],
[190.0, 190.0, 205.0, 205.0],
[215.0, 215.0, 225.0, 225.0],
[197.5, 197.5, 220.0, 220.0],
[235.0, 235.0, 245.0, 245.0],
[240.0, 240.0, 255.0, 255.0],
[208.75, 208.75, 247.5, 247.5],
[265.0, 265.0, 275.0, 275.0],
[270.0, 270.0, 285.0, 285.0],
[295.0, 295.0, 305.0, 305.0],
[300.0, 300.0, 315.0, 315.0],
[277.5, 277.5, 307.5, 307.5],
[228.125, 228.125, 292.5, 292.5],
[111.875, 111.875, 260.3125, 260.3125],
[325.0, 325.0, 335.0, 335.0],
[330.0, 330.0, 345.0, 345.0],
[355.0, 355.0, 365.0, 365.0],
[360.0, 360.0, 375.0, 375.0],
[337.5, 337.5, 367.5, 367.5],
[385.0, 385.0, 395.0, 395.0],
[405.0, 405.0, 415.0, 415.0],
[390.0, 390.0, 410.0, 410.0],
[352.5, 352.5, 400.0, 400.0],
[425.0, 425.0, 435.0, 435.0],
[430.0, 430.0, 445.0, 445.0],
[437.5, 437.5, 455.0, 455.0],
[376.25, 376.25, 446.25, 446.25],
[465.0, 465.0, 475.0, 475.0],
[485.0, 485.0, 495.0, 495.0],
[470.0, 470.0, 490.0, 490.0],
```

```
[505.0, 505.0, 515.0, 515.0],
[510.0, 510.0, 525.0, 525.0],
[480.0, 480.0, 517.5, 517.5],
[411.25, 411.25, 498.75, 498.75],
[535.0, 535.0, 545.0, 545.0],
[555.0, 555.0, 565.0, 565.0],
[540.0, 540.0, 560.0, 560.0],
[575.0, 575.0, 585.0, 585.0],
[550.0, 550.0, 580.0, 580.0],
[565.0, 565.0, 595.0, 595.0],
[605.0, 605.0, 615.0, 615.0],
[625.0, 625.0, 635.0, 635.0],
[610.0, 610.0, 630.0, 630.0],
[645.0, 645.0, 655.0, 655.0],
[650.0, 650.0, 665.0, 665.0],
[620.0, 620.0, 657.5, 657.5],
[580.0, 580.0, 638.75, 638.75],
[675.0, 675.0, 685.0, 685.0],
[680.0, 680.0, 695.0, 695.0],
[705.0, 705.0, 715.0, 715.0],
[687.5, 687.5, 710.0, 710.0],
[609.375, 609.375, 698.75, 698.75],
[725.0, 725.0, 735.0, 735.0],
[730.0, 730.0, 745.0, 745.0],
[755.0, 755.0, 765.0, 765.0],
[737.5, 737.5, 760.0, 760.0],
[654.0625, 654.0625, 748.75, 748.75],
[455.0, 455.0, 701.40625, 701.40625],
[775.0, 775.0, 785.0, 785.0],
[795.0, 795.0, 805.0, 805.0],
[780.0, 780.0, 800.0, 800.0],
[815.0, 815.0, 825.0, 825.0],
[820.0, 820.0, 835.0, 835.0],
[827.5, 827.5, 845.0, 845.0],
[790.0, 790.0, 836.25, 836.25],
```

```
[855.0, 855.0, 865.0, 865.0],
[860.0, 860.0, 875.0, 875.0],
[885.0, 885.0, 895.0, 895.0],
[905.0, 905.0, 915.0, 915.0],
[890.0, 890.0, 910.0, 910.0],
[925.0, 925.0, 935.0, 935.0],
[930.0, 930.0, 945.0, 945.0],
[900.0, 900.0, 937.5, 937.5],
[867.5, 867.5, 918.75, 918.75],
[813.125, 813.125, 893.125, 893.125],
[578.203125, 578.203125, 853.125, 853.125],
[955.0, 955.0, 965.0, 965.0],
[960.0, 960.0, 975.0, 975.0],
[967.5, 967.5, 985.0, 985.0],
[995.0, 995.0, 1005.0, 1005.0],
[1000.0, 1000.0, 1015.0, 1015.0],
[976.25, 976.25, 1007.5, 1007.5],
[1025.0, 1025.0, 1035.0, 1035.0],
[1030.0, 1030.0, 1045.0, 1045.0],
[991.875, 991.875, 1037.5, 1037.5],
[1055.0, 1055.0, 1065.0, 1065.0],
[1060.0, 1060.0, 1075.0, 1075.0],
[1014.6875, 1014.6875, 1067.5, 1067.5],
[715.6640625, 715.6640625, 1041.09375, 1041.09375],
[186.09375, 186.09375, 878.37890625, 878.37890625],
[1085.0, 1085.0, 1095.0, 1095.0],
[1090.0, 1090.0, 1105.0, 1105.0],
[1115.0, 1115.0, 1125.0, 1125.0],
[1120.0, 1120.0, 1135.0, 1135.0],
[1097.5, 1097.5, 1127.5, 1127.5],
[1145.0, 1145.0, 1155.0, 1155.0],
[1150.0, 1150.0, 1165.0, 1165.0],
[1112.5, 1112.5, 1157.5, 1157.5],
[1175.0, 1175.0, 1185.0, 1185.0],
[1180.0, 1180.0, 1195.0, 1195.0],
```

```
 [1205.0, 1205.0, 1215.0, 1215.0],
 [1210.0, 1210.0, 1225.0, 1225.0],
 [1187.5, 1187.5, 1217.5, 1217.5],
 [1235.0, 1235.0, 1245.0, 1245.0],
 [1240.0, 1240.0, 1255.0, 1255.0],
 [1202.5, 1202.5, 1247.5, 1247.5],
 [1135.0, 1135.0, 1225.0, 1225.0],
 [1265.0, 1265.0, 1275.0, 1275.0],
 [1270.0, 1270.0, 1285.0, 1285.0],
 [1295.0, 1295.0, 1305.0, 1305.0],
 [1300.0, 1300.0, 1315.0, 1315.0],
 [1277.5, 1277.5, 1307.5, 1307.5],
 [1325.0, 1325.0, 1335.0, 1335.0],
 [1330.0, 1330.0, 1345.0, 1345.0],
 [1292.5, 1292.5, 1337.5, 1337.5],
 [1180.0, 1180.0, 1315.0, 1315.0],
 [532.236328125, 532.236328125, 1247.5, 1247.5]],
'dcoord': [[0.0, 1.8532145500212729, 1.8532145500212729, 0.0],
 [0.0, 1.8456515841592327, 1.8456515841592327, 0.0],
 [1.8532145500212729,
  3.0162940483033576,
  3.0162940483033576,
  1.8456515841592327],
 [0.0, 1.6510198648037537, 1.6510198648037537, 0.0],
 [1.6510198648037537, 2.6987507586592594, 2.6987507586592594,␣
↪0.0],
 [3.0162940483033576,
  3.202881700688482,
  3.202881700688482,
  2.6987507586592594],
 [0.0, 1.8061649278153957, 1.8061649278153957, 0.0],
 [1.8061649278153957, 2.330722522476597, 2.330722522476597, 0.
↪0],
 [0.0, 2.062672979625104, 2.062672979625104, 0.0],
 [2.330722522476597,
```

```
 3.0695543343572678,
 3.0695543343572678,
 2.062672979625104],
[3.202881700688482,
 4.127131573378693,
 4.127131573378693,
 3.0695543343572678],
[0.0, 1.1420094743570053, 1.1420094743570053, 0.0],
[0.0, 1.011108468334543, 1.011108468334543, 0.0],
[1.1420094743570053,
 2.368958575818999,
 2.368958575818999,
 1.011108468334543],
[0.0, 1.2110743431869655, 1.2110743431869655, 0.0],
[2.368958575818999,
 2.652240521202772,
 2.652240521202772,
 1.2110743431869655],
[4.127131573378693, 4.850647084098702, 4.850647084098702, 2.
↪652240521202772],
[0.0, 1.6618764419531307, 1.6618764419531307, 0.0],
[1.6618764419531307, 2.21030445573427, 2.21030445573427, 0.
↪0],
[0.0, 1.6239426993893555, 1.6239426993893555, 0.0],
[2.21030445573427,
 3.2836905484132592,
 3.2836905484132592,
 1.6239426993893555],
[0.0, 1.7834368238051554, 1.7834368238051554, 0.0],
[1.7834368238051554, 2.3836987706865274, 2.3836987706865274,␣
↪0.0],
[3.2836905484132592,
 3.6497020331077916,
 3.6497020331077916,
 2.3836987706865274],
```

```
[0.0, 2.8654854380148613, 2.8654854380148613, 0.0],
[2.8654854380148613, 3.1436198959418564, 3.1436198959418564,␣
↪0.0],
[0.0, 2.2276492449266225, 2.2276492449266225, 0.0],
[2.2276492449266225, 2.3821037302171946, 2.3821037302171946,␣
↪0.0],
[3.1436198959418564,
 3.261683361487948,
 3.261683361487948,
 2.3821037302171946],
[3.6497020331077916,
 4.701777570952728,
 4.701777570952728,
 3.261683361487948],
[4.850647084098702, 5.525155154456377, 5.525155154456377, 4.
↪701777570952728],
[0.0, 0.5223652348100113, 0.5223652348100113, 0.0],
[0.5223652348100113, 0.6930701774197707, 0.6930701774197707,␣
↪0.0],
[0.0, 0.5381155751240019, 0.5381155751240019, 0.0],
[0.5381155751240019, 0.6310169386326535, 0.6310169386326535,␣
↪0.0],
[0.6930701774197707,
 0.8274046324527276,
 0.8274046324527276,
 0.6310169386326535],
[0.0, 0.577579395035639, 0.577579395035639, 0.0],
[0.0, 0.4642111109291484, 0.4642111109291484, 0.0],
[0.577579395035639,
 0.7629738647926234,
 0.7629738647926234,
 0.4642111109291484],
[0.8274046324527276,
 0.9722512208438004,
 0.9722512208438004,
```

```
 0.7629738647926234],
[0.0, 0.5988724670909609, 0.5988724670909609, 0.0],
[0.5988724670909609, 0.6676394206813703, 0.6676394206813703,␣
↪0.0],
[0.6676394206813703, 0.7496976080475588, 0.7496976080475588,␣
↪0.0],
[0.9722512208438004,
 1.2023776809652837,
 1.2023776809652837,
 0.7496976080475588],
[0.0, 0.5226945668670357, 0.5226945668670357, 0.0],
[0.0, 0.5037924216717151, 0.5037924216717151, 0.0],
[0.5226945668670357,
 0.6099772044064197,
 0.6099772044064197,
 0.5037924216717151],
[0.0, 0.546017620819854, 0.546017620819854, 0.0],
[0.546017620819854, 0.5553450972070503, 0.5553450972070503,␣
↪0.0],
[0.6099772044064197,
 0.9822666261709275,
 0.9822666261709275,
 0.5553450972070503],
[1.2023776809652837,
 1.3728519086253423,
 1.3728519086253423,
 0.9822666261709275],
[0.0, 0.632244342052007, 0.632244342052007, 0.0],
[0.0, 0.5538267352968257, 0.5538267352968257, 0.0],
[0.632244342052007,
 0.7173744448505187,
 0.7173744448505187,
 0.5538267352968257],
[0.0, 0.6280370483755658, 0.6280370483755658, 0.0],
[0.7173744448505187,
```

```
 0.7993434543264835,
 0.7993434543264835,
 0.6280370483755658],
[0.7993434543264835, 0.8620960528488291, 0.8620960528488291,␣
↪0.0],
[0.0, 0.44429238513326913, 0.44429238513326913, 0.0],
[0.0, 0.3946797225789737, 0.3946797225789737, 0.0],
[0.44429238513326913,
 0.5229857243596863,
 0.5229857243596863,
 0.3946797225789737],
[0.0, 0.40656868391870754, 0.40656868391870754, 0.0],
[0.40656868391870754, 0.4853221294724772, 0.
↪4853221294724772, 0.0],
[0.5229857243596863,
 0.5597987975277805,
 0.5597987975277805,
 0.4853221294724772],
[0.8620960528488291,
 0.8762604596875789,
 0.8762604596875789,
 0.5597987975277805],
[0.0, 0.6794018628343776, 0.6794018628343776, 0.0],
[0.6794018628343776, 0.7400059739387083, 0.7400059739387083,␣
↪0.0],
[0.0, 0.5891178383428738, 0.5891178383428738, 0.0],
[0.7400059739387083,
 0.8226974859036109,
 0.8226974859036109,
 0.5891178383428738],
[0.8762604596875789,
 1.0128451509474072,
 1.0128451509474072,
 0.8226974859036109],
[0.0, 0.6711648132322598, 0.6711648132322598, 0.0],
```

```
 [0.6711648132322598, 0.7083527456286032, 0.7083527456286032,␣
↪0.0],
 [0.0, 0.6158491709368314, 0.6158491709368314, 0.0],
 [0.7083527456286032,
  0.9459585446054516,
  0.9459585446054516,
  0.6158491709368314],
 [1.0128451509474072,
  1.3183684480915157,
  1.3183684480915157,
  0.9459585446054516],
 [1.3728519086253423,
  1.7059209914806512,
  1.7059209914806512,
  1.3183684480915157],
 [0.0, 0.5335968237533587, 0.5335968237533587, 0.0],
 [0.0, 0.4765369089835595, 0.4765369089835595, 0.0],
 [0.5335968237533587,
  0.6712753427124933,
  0.6712753427124933,
  0.4765369089835595],
 [0.0, 0.4935903624917718, 0.4935903624917718, 0.0],
 [0.4935903624917718, 0.5726189498138283, 0.5726189498138283,␣
↪0.0],
 [0.5726189498138283, 0.6249588759286095, 0.6249588759286095,␣
↪0.0],
 [0.6712753427124933,
  0.8561959180911473,
  0.8561959180911473,
  0.6249588759286095],
 [0.0, 0.5844696606530146, 0.5844696606530146, 0.0],
 [0.5844696606530146, 0.6782450767096722, 0.6782450767096722,␣
↪0.0],
 [0.0, 0.3980966429401672, 0.3980966429401672, 0.0],
 [0.0, 0.3900498417224389, 0.3900498417224389, 0.0],
```

```
[0.3980966429401672,
 0.5118946026200807,
 0.5118946026200807,
 0.3900498417224389],
[0.0, 0.387703554436143, 0.387703554436143, 0.0],
[0.387703554436143, 0.4568505477917489, 0.4568505477917489,␣
↪0.0],
[0.5118946026200807,
 0.6134373863002032,
 0.6134373863002032,
 0.4568505477917489],
[0.6782450767096722,
 0.8370363524518297,
 0.8370363524518297,
 0.6134373863002032],
[0.8561959180911473,
 1.152071971107537,
 1.152071971107537,
 0.8370363524518297],
[1.7059209914806512, 1.84439510676065, 1.84439510676065, 1.
↪152071971107537],
[0.0, 0.42007803211029837, 0.42007803211029837, 0.0],
[0.42007803211029837, 0.4907305132216974, 0.
↪4907305132216974, 0.0],
[0.4907305132216974, 0.5816906829042745, 0.5816906829042745,␣
↪0.0],
[0.0, 0.4301266947060181, 0.4301266947060181, 0.0],
[0.4301266947060181, 0.49032658819854774, 0.
↪49032658819854774, 0.0],
[0.5816906829042745,
 0.9398975224002967,
 0.9398975224002967,
 0.49032658819854774],
[0.0, 0.4366244602521761, 0.4366244602521761, 0.0],
```

```
[0.4366244602521761, 0.450715776565841, 0.450715776565841, 0.
↪0],
[0.9398975224002967,
 1.034305932933774,
 1.034305932933774,
 0.450715776565841],
[0.0, 0.4191694379910241, 0.4191694379910241, 0.0],
[0.4191694379910241, 0.47330634665958254, 0.
↪47330634665958254, 0.0],
[1.034305932933774,
 1.1399168252819865,
 1.1399168252819865,
 0.47330634665958254],
[1.84439510676065,
 2.4153771200277774,
 2.4153771200277774,
 1.1399168252819865],
[5.525155154456377,
 6.893699916633943,
 6.893699916633943,
 2.4153771200277774],
[0.0, 0.8481592242371075, 0.8481592242371075, 0.0],
[0.8481592242371075, 1.2098195509339489, 1.2098195509339489,␣
↪0.0],
[0.0, 0.897158119893585, 0.897158119893585, 0.0],
[0.897158119893585, 1.1281469483745448, 1.1281469483745448,␣
↪0.0],
[1.2098195509339489,
 2.5049416522660364,
 2.5049416522660364,
 1.1281469483745448],
[0.0, 0.9023277295074331, 0.9023277295074331, 0.0],
[0.9023277295074331, 1.3277721349877274, 1.3277721349877274,␣
↪0.0],
[2.5049416522660364,
```

```
 2.778684968181474,
 2.778684968181474,
 1.3277721349877274],
[0.0, 0.6536934893011386, 0.6536934893011386, 0.0],
[0.6536934893011386, 1.3160977422067337, 1.3160977422067337,␣
↪0.0],
[0.0, 0.9102366106360873, 0.9102366106360873, 0.0],
[0.9102366106360873, 1.1054621474347133, 1.1054621474347133,␣
↪0.0],
[1.3160977422067337,
 2.029028798742102,
 2.029028798742102,
 1.1054621474347133],
[0.0, 0.6117639802700021, 0.6117639802700021, 0.0],
[0.6117639802700021, 1.2367296208221674, 1.2367296208221674,␣
↪0.0],
[2.029028798742102,
 2.588180529437198,
 2.588180529437198,
 1.2367296208221674],
[2.778684968181474, 5.149689693574178, 5.149689693574178, 2.
↪588180529437198],
[0.0, 0.8967460839650845, 0.8967460839650845, 0.0],
[0.8967460839650845, 1.2303066737507315, 1.2303066737507315,␣
↪0.0],
[0.0, 0.7748564615777291, 0.7748564615777291, 0.0],
[0.7748564615777291, 1.0974109174971909, 1.0974109174971909,␣
↪0.0],
[1.2303066737507315,
 2.5734439867711996,
 2.5734439867711996,
 1.0974109174971909],
[0.0, 0.8407192846726442, 0.8407192846726442, 0.0],
[0.8407192846726442, 1.2912211692125974, 1.2912211692125974,␣
↪0.0],
```

```
  [2.5734439867711996,
   2.992416207597669,
   2.992416207597669,
   1.2912211692125974],
  [5.149689693574178,
   5.9189277627777415,
   5.9189277627777415,
   2.992416207597669],
  [6.893699916633943, 8.47238109067575, 8.47238109067575, 5.
↪9189277627777415]],
 'ivl': ['135__H',
  '129__H',
  '132__H',
  '131__H',
  '133__H',
  '125__H',
  '118__H',
  '134__H',
  '127__H',
  '88__H',
  '120__H',
  '90__H',
  '29__H',
  '28__H',
  '26__H',
  '25__H',
  '122__H',
  '121__H',
  '100__H',
  '92__H',
  '102__H',
  '114__H',
  '110__H',
  '96__H',
  '94__H',
```

```
'86__H',
'108__H',
'106__H',
'98__H',
'112__H',
'104__H',
'116__H',
'119__O',
'117__O',
'93__O',
'91__O',
'89__O',
'87__O',
'99__O',
'95__O',
'97__O',
'85__O',
'128__O',
'124__O',
'126__O',
'130__O',
'31__O',
'30__O',
'32__O',
'27__P',
'23__C',
'22__C',
'24__C',
'44__O',
'18__O',
'5__O',
'2__O',
'8__O',
'1__O',
'71__O',
```

```
'35__Si',
'21__Si',
'19__Si',
'14__Si',
'4__Si',
'3__Si',
'17__Si',
'20__O',
'16__O',
'15__O',
'13__O',
'10__O',
'39__O',
'7__O',
'36__O',
'34__O',
'33__O',
'115__O',
'113__O',
'103__O',
'101__O',
'111__O',
'107__O',
'105__O',
'109__O',
'43__O',
'40__O',
'11__O',
'42__Si',
'37__Si',
'41__Si',
'38__Si',
'9__Si',
'6__Si',
'12__Si',
```

```
'72__Si',
'45__Si',
'58__Si',
'123__Cu',
'54__C',
'46__C',
'50__C',
'81__C',
'77__C',
'73__C',
'67__C',
'63__C',
'59__C',
'80__H',
'79__H',
'78__H',
'84__H',
'83__H',
'82__H',
'76__H',
'75__H',
'74__H',
'62__H',
'61__H',
'60__H',
'66__H',
'64__H',
'65__H',
'69__H',
'68__H',
'70__H',
'48__H',
'47__H',
'49__H',
'53__H',
```

```
 '52__H',
 '51__H',
 '57__H',
 '56__H',
 '55__H'],
'leaves': [134,
 128,
 131,
 130,
 132,
 124,
 117,
 133,
 126,
 87,
 119,
 89,
 28,
 27,
 25,
 24,
 121,
 120,
 99,
 91,
 101,
 113,
 109,
 95,
 93,
 85,
 107,
 105,
 97,
 111,
```

```
103,
115,
118,
116,
92,
90,
88,
86,
98,
94,
96,
84,
127,
123,
125,
129,
30,
29,
31,
26,
22,
21,
23,
43,
17,
4,
1,
7,
0,
70,
34,
20,
18,
13,
3,
```

2,
16,
19,
15,
14,
12,
9,
38,
6,
35,
33,
32,
114,
112,
102,
100,
110,
106,
104,
108,
42,
39,
10,
41,
36,
40,
37,
8,
5,
11,
71,
44,
57,
122,
53,

```
45,
49,
80,
76,
72,
66,
62,
58,
79,
78,
77,
83,
82,
81,
75,
74,
73,
61,
60,
59,
65,
63,
64,
68,
67,
69,
47,
46,
48,
52,
51,
50,
56,
55,
54],
```

```
'color_list': ['C1',
 'C1',
 'C1',
 'C1',
 'C1',
 'C1',
 'C1',
 'C1',
 'C1',
 'C1',
 'C1',
 'C1',
 'C1',
 'C1',
 'C1',
 'C1',
 'C1',
 'C1',
 'C1',
 'C1',
 'C1',
 'C1',
 'C1',
 'C1',
 'C1',
 'C1',
 'C1',
 'C1',
 'C1',
 'C1',
 'C1',
 'C2',
 'C2',
 'C2',
 'C2',
```

```
'C2',
'C2',
'C2',
'C2',
'C2',
'C2',
'C2',
'C2',
'C2',
'C2',
'C2',
'C2',
'C2',
'C2',
'C2',
'C2',
'C2',
'C2',
'C2',
'C2',
'C2',
'C2',
'C2',
'C2',
'C2',
'C2',
'C2',
'C2',
'C2',
'C2',
'C2',
'C2',
'C2',
'C2',
'C2',
```

```
'C2',
'C2',
'C2',
'C2',
'C2',
'C2',
'C2',
'C2',
'C2',
'C2',
'C2',
'C2',
'C2',
'C2',
'C2',
'C2',
'C2',
'C2',
'C2',
'C2',
'C2',
'C2',
'C2',
'C2',
'C2',
'C2',
'C2',
'C2',
'C2',
'C2',
'C2',
'C2',
'C2',
'C2',
'C2',
```

```
 'C2',
 'C0',
 'C3',
 'C3',
 'C3',
 'C3',
 'C3',
 'C3',
 'C3',
 'C3',
 'C3',
 'C3',
 'C3',
 'C3',
 'C3',
 'C3',
 'C3',
 'C3',
 'C3',
 'C3',
 'C3',
 'C3',
 'C3',
 'C3',
 'C3',
 'C3',
 'C3',
 'C3',
 'C0'],
'leaves_color_list': ['C1',
 'C1',
 'C1',
 'C1',
 'C1',
 'C1',
```

```
'C1',
'C1',
'C1',
'C1',
'C1',
'C1',
'C1',
'C1',
'C1',
'C1',
'C1',
'C1',
'C1',
'C1',
'C1',
'C1',
'C1',
'C1',
'C1',
'C1',
'C1',
'C1',
'C1',
'C1',
'C1',
'C1',
'C2',
'C2',
'C2',
'C2',
'C2',
'C2',
'C2',
'C2',
'C2',
```

```
'C2',
'C2',
'C2',
'C2',
'C2',
'C2',
'C2',
'C2',
'C2',
'C2',
'C2',
'C2',
'C2',
'C2',
'C2',
'C2',
'C2',
'C2',
'C2',
'C2',
'C2',
'C2',
'C2',
'C2',
'C2',
'C2',
'C2',
'C2',
'C2',
'C2',
'C2',
'C2',
'C2',
'C2',
'C2',
```

```
'C2',
'C2',
'C2',
'C2',
'C2',
'C2',
'C2',
'C2',
'C2',
'C2',
'C2',
'C2',
'C2',
'C2',
'C2',
'C2',
'C2',
'C2',
'C2',
'C2',
'C2',
'C2',
'C2',
'C2',
'C2',
'C2',
'C2',
'C2',
'C2',
'C2',
'C2',
'C2',
'C3',
'C3',
'C3',
```

```
'C3',
'C3',
'C3',
'C3',
'C3',
'C3',
'C3',
'C3',
'C3',
'C3',
'C3',
'C3',
'C3',
'C3',
'C3',
'C3',
'C3',
'C3',
'C3',
'C3',
'C3',
'C3',
'C3',
'C3']}
```

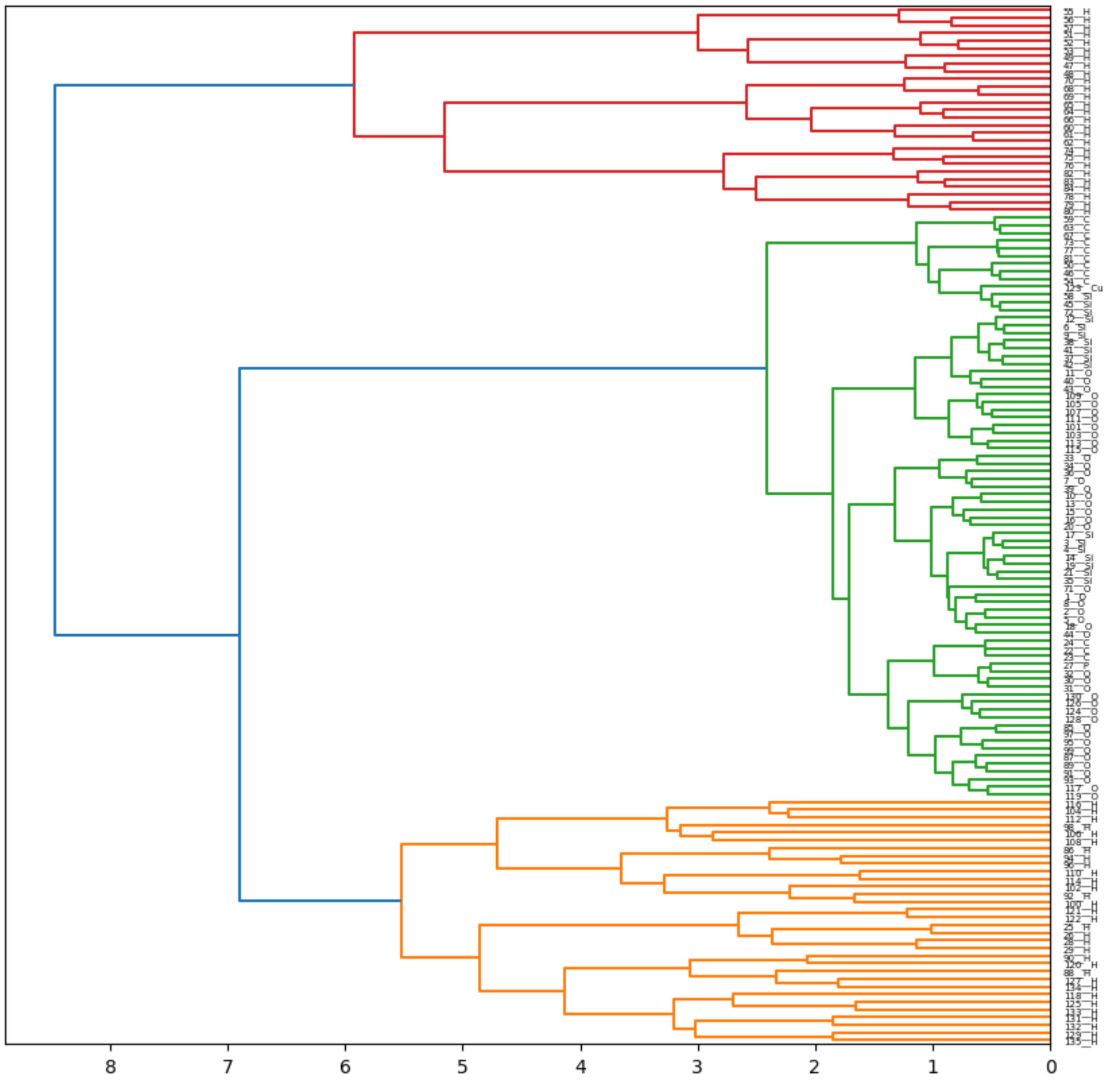

[9]:
```
plt.figure(figsize=(26,9))
sns.heatmap(df2.loc[:], cmap="Blues")#, xticklabels=1,␣
 ↪yticklabels=1)
plt.title(" ")
plt.xlabel(" ")
plt.ylabel(" ")
```

[9]: Text(295.58159722222223, 0.5, 'nazwa atomu')

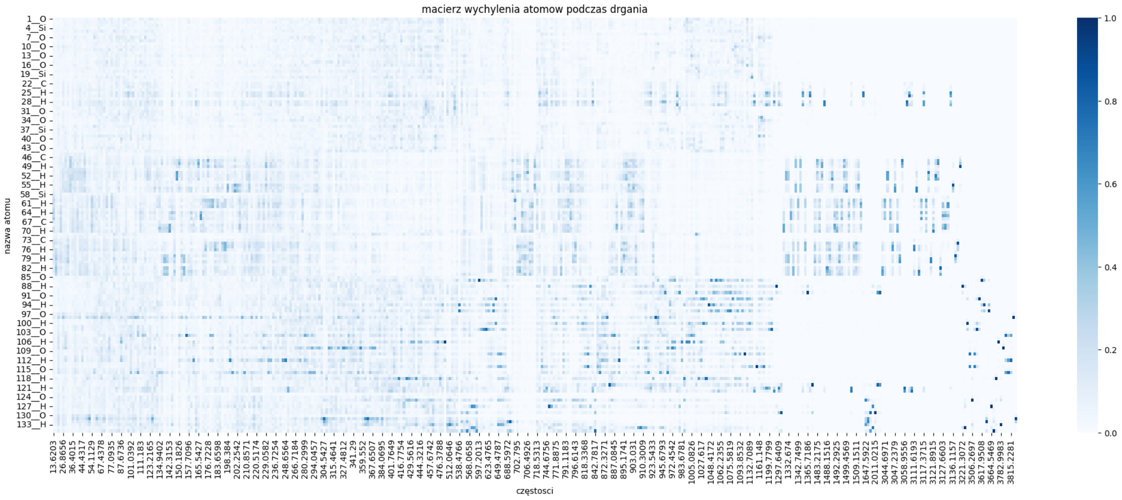

[10]:
```
linked
```

[10]:
```
array([[  5.        ,   8.        ,   0.38770355,   2.        ␣
→],
       [ 37.        ,  40.        ,   0.39004984,   2.        ␣
→],
       [ 13.        ,  18.        ,   0.39467972,   2.        ␣
→],
       [ 36.        ,  41.        ,   0.39809664,   2.        ␣
→],
       [  2.        ,   3.        ,   0.40656868,   2.        ␣
→],
       [ 62.        ,  66.        ,   0.41916944,   2.        ␣
→],
       [ 44.        ,  71.        ,   0.42007803,   2.        ␣
→],
       [ 45.        ,  53.        ,   0.43012669,   2.        ␣
→],
       [ 76.        ,  80.        ,   0.43662446,   2.        ␣
→],
       [ 20.        ,  34.        ,   0.44429239,   2.        ␣
→],
```

```
       [ 72.        , 143.        ,   0.45071578,   3.        ␣
↪],
       [ 11.        , 135.        ,   0.45685055,   3.        ␣
↪],
       [ 84.        ,  96.        ,   0.46421111,   2.        ␣
↪],
       [ 58.        , 140.        ,   0.47330635,   3.        ␣
↪],
       [100.        , 102.        ,   0.47653691,   2.        ␣
↪],
       [ 16.        , 139.        ,   0.48532213,   3.        ␣
↪],
       [ 49.        , 142.        ,   0.49032659,   3.        ␣
↪],
       [ 57.        , 141.        ,   0.49073051,   3.        ␣
↪],
       [106.        , 110.        ,   0.49359036,   2.        ␣
↪],
       [ 26.        ,  31.        ,   0.50379242,   2.        ␣
↪],
       [136.        , 138.        ,   0.5118946 ,   4.        ␣
↪],
       [116.        , 118.        ,   0.52236523,   2.        ␣
↪],
       [ 29.        ,  30.        ,   0.52269457,   2.        ␣
↪],
       [137.        , 144.        ,   0.52298572,   4.        ␣
↪],
       [112.        , 114.        ,   0.53359682,   2.        ␣
↪],
       [ 88.        ,  90.        ,   0.53811558,   2.        ␣
↪],
       [ 21.        ,  22.        ,   0.54601762,   2.        ␣
↪],
```

```
        [  1.        ,   4.        ,   0.55382674,   2.        ␣
↪],
        [ 23.        , 161.        ,   0.5553451 ,   3.        ␣
↪],
        [150.        , 158.        ,   0.5597988 ,   7.        ␣
↪],
        [104.        , 153.        ,   0.57261895,   3.        ␣
↪],
        [ 94.        ,  98.        ,   0.5775794 ,   2.        ␣
↪],
        [122.        , 152.        ,   0.58169068,   4.        ␣
↪],
        [ 39.        ,  42.        ,   0.58446966,   2.        ␣
↪],
        [  9.        ,  12.        ,   0.58911784,   2.        ␣
↪],
        [123.        , 127.        ,   0.59887247,   2.        ␣
↪],
        [154.        , 157.        ,   0.6099772 ,   4.        ␣
↪],
        [ 67.        ,  68.        ,   0.61176398,   2.        ␣
↪],
        [146.        , 155.        ,   0.61343739,   7.        ␣
↪],
        [ 32.        ,  33.        ,   0.61584917,   2.        ␣
↪],
        [108.        , 165.        ,   0.62495888,   4.        ␣
↪],
        [  0.        ,   7.        ,   0.62803705,   2.        ␣
↪],
        [ 86.        , 160.        ,   0.63101694,   3.        ␣
↪],
        [ 17.        ,  43.        ,   0.63224434,   2.        ␣
↪],
```

```
        [ 60.        ,  61.        ,   0.65369349,   2.        ␣
↪],
        [125.        , 170.        ,   0.66763942,   3.        ␣
↪],
        [  6.        ,  38.        ,   0.67116481,   2.        ␣
↪],
        [149.        , 159.        ,   0.67127534,   4.        ␣
↪],
        [ 10.        , 168.        ,   0.67824508,   3.        ␣
↪],
        [ 15.        ,  19.        ,   0.67940186,   2.        ␣
↪],
        [ 92.        , 156.        ,   0.69307018,   3.        ␣
↪],
        [ 35.        , 181.        ,   0.70835275,   3.        ␣
↪],
        [162.        , 178.        ,   0.71737444,   4.        ␣
↪],
        [ 14.        , 184.        ,   0.74000597,   3.        ␣
↪],
        [129.        , 180.        ,   0.74969761,   4.        ␣
↪],
        [147.        , 166.        ,   0.76297386,   4.        ␣
↪],
        [ 51.        ,  52.        ,   0.77485646,   2.        ␣
↪],
        [176.        , 187.        ,   0.79934345,   6.        ␣
↪],
        [169.        , 188.        ,   0.82269749,   5.        ␣
↪],
        [177.        , 185.        ,   0.82740463,   6.        ␣
↪],
        [173.        , 183.        ,   0.83703635,  10.        ␣
↪],
```

```
       [ 55.        ,  56.        ,   0.84071928,   2.        ␣
↪],
       [ 78.        ,  79.        ,   0.84815922,   2.        ␣
↪],
       [175.        , 182.        ,   0.85619592,   8.        ␣
↪],
       [ 70.        , 192.        ,   0.86209605,   7.        ␣
↪],
       [164.        , 199.        ,   0.87626046,  14.        ␣
↪],
       [ 46.        ,  47.        ,   0.89674608,   2.        ␣
↪],
       [ 82.        ,  83.        ,   0.89715812,   2.        ␣
↪],
       [ 74.        ,  75.        ,   0.90232773,   2.        ␣
↪],
       [ 63.        ,  65.        ,   0.91023661,   2.        ␣
↪],
       [151.        , 167.        ,   0.93989752,   7.        ␣
↪],
       [174.        , 186.        ,   0.94595854,   5.        ␣
↪],
       [190.        , 194.        ,   0.97225122,  10.        ␣
↪],
       [163.        , 171.        ,   0.98226663,   7.        ␣
↪],
       [ 24.        ,  25.        ,   1.01110847,   2.        ␣
↪],
       [193.        , 200.        ,   1.01284515,  19.        ␣
↪],
       [145.        , 205.        ,   1.03430593,  10.        ␣
↪],
       [ 50.        , 191.        ,   1.09741092,   3.        ␣
↪],
```

```
        [ 64.         , 204.         ,   1.10546215,   3.        ␣
↪],
        [ 81.         , 202.         ,   1.12814695,   3.        ␣
↪],
        [148.         , 211.         ,   1.13991683,  13.        ␣
↪],
        [ 27.         ,  28.         ,   1.14200947,   2.        ␣
↪],
        [195.         , 198.         ,   1.15207197,  18.        ␣
↪],
        [189.         , 207.         ,   1.20237768,  14.        ␣
↪],
        [ 77.         , 197.         ,   1.20981955,   3.        ␣
↪],
        [120.         , 121.         ,   1.21107434,   2.        ␣
↪],
        [ 48.         , 201.         ,   1.23030667,   3.        ␣
↪],
        [ 69.         , 172.         ,   1.23672962,   3.        ␣
↪],
        [ 54.         , 196.         ,   1.29122117,   3.        ␣
↪],
        [ 59.         , 179.         ,   1.31609774,   3.        ␣
↪],
        [206.         , 210.         ,   1.31836845,  24.        ␣
↪],
        [ 73.         , 203.         ,   1.32777213,   3.        ␣
↪],
        [208.         , 218.         ,   1.37285191,  21.        ␣
↪],
        [109.         , 113.         ,   1.6239427 ,   2.        ␣
↪],
        [124.         , 132.         ,   1.65101986,   2.        ␣
↪],
```

```
       [ 91.        ,  99.        ,   1.66187644,   2.        ␣
↪],
       [225.        , 227.        ,   1.70592099,  45.        ␣
↪],
       [ 93.        ,  95.        ,   1.78343682,   2.        ␣
↪],
       [126.        , 133.        ,   1.80616493,   2.        ␣
↪],
       [217.        , 231.        ,   1.84439511,  63.        ␣
↪],
       [130.        , 131.        ,   1.84565158,   2.        ␣
↪],
       [128.        , 134.        ,   1.85321455,   2.        ␣
↪],
       [213.        , 224.        ,   2.0290288 ,   6.        ␣
↪],
       [ 89.        , 119.        ,   2.06267298,   2.        ␣
↪],
       [101.        , 230.        ,   2.21030446,   3.        ␣
↪],
       [103.        , 111.        ,   2.22764924,   2.        ␣
↪],
       [ 87.        , 233.        ,   2.33072252,   3.        ␣
↪],
       [209.        , 216.        ,   2.36895858,   4.        ␣
↪],
       [115.        , 240.        ,   2.38210373,   3.        ␣
↪],
       [ 85.        , 232.        ,   2.38369877,   3.        ␣
↪],
       [215.        , 234.        ,   2.41537712,  76.        ␣
↪],
       [214.        , 219.        ,   2.50494165,   6.        ␣
↪],
```

```
        [212.          , 221.          ,   2.57344399,   6.          ␣
↪],
        [222.          , 237.          ,   2.58818053,   9.          ␣
↪],
        [220.          , 242.          ,   2.65224052,   6.          ␣
↪],
        [117.          , 229.          ,   2.69875076,   3.          ␣
↪],
        [226.          , 246.          ,   2.77868497,   9.          ␣
↪],
        [105.          , 107.          ,   2.86548544,   2.          ␣
↪],
        [223.          , 247.          ,   2.99241621,   9.          ␣
↪],
        [235.          , 236.          ,   3.01629405,   4.          ␣
↪],
        [238.          , 241.          ,   3.06955433,   5.          ␣
↪],
        [ 97.          , 252.          ,   3.1436199 ,   3.          ␣
↪],
        [250.          , 254.          ,   3.2028817 ,   7.          ␣
↪],
        [243.          , 256.          ,   3.26168336,   6.          ␣
↪],
        [228.          , 239.          ,   3.28369055,   5.          ␣
↪],
        [244.          , 259.          ,   3.64970203,   8.          ␣
↪],
        [255.          , 257.          ,   4.12713157,  12.          ␣
↪],
        [258.          , 260.          ,   4.70177757,  14.          ␣
↪],
        [249.          , 261.          ,   4.85064708,  18.          ␣
↪],
```

```
        [248.        , 251.        ,   5.14968969,  18.        ␣
↪],
        [262.        , 263.        ,   5.52515515,  32.        ␣
↪],
        [253.        , 264.        ,   5.91892776,  27.        ␣
↪],
        [245.        , 265.        ,   6.89369992, 108.        ␣
↪],
        [266.        , 267.        ,   8.47238109, 135.        ␣
↪]])
```

[11]:
```
###
```

[12]:
```
# MANUAL
#
FILENAMElog = NAZWAPLIKU
FILENAME = FILENAMElog.replace(".log", "")
print(FILENAME)
Y_RAMANmat = pd.read_csv(FILENAME+"_decomposition_RAMAN.csv",␣
↪header=0, dtype={'Unnamed: 0':str})
Y_RAMANmat.set_index('Unnamed: 0', inplace=True)







####### ALL
#
#
listaH = []
for i in Y_RAMANmat.index:
    #print(i.strip().split("_")[2] )
    if(i.strip().split("_")[2]=="ZZZZ"):
        print(i.strip().split("_")[2])
    else:
```

```
        listaH.append(int(i.strip().split("_")[0])-1)
print("Lista wszystkich atomów: "+ str([element + 1 for␣
 ↪element in listaH]))
print( Y_RAMANmat.index[listaH])
czasteczkaH = []
czasteczkaH.extend([(len(listaH)),"\n\n"] )

# + for i in listaH: str(i) )
file = open(FILENAME+"_XYZcenter.xyz", 'r')
lista_linii = file.readlines()
file.close()
for i in listaH:
    czasteczkaH.append(lista_linii[i+2])
#print(czasteczkaH)


with open('czasteczkaH.xyz', 'w') as file:
    for element in czasteczkaH:
        file.write(str(element) + '')


view = py3Dmol.view(width=650, height=650)
view.setStyle({'sphere':{}}, viewer=(0,2))
view.addModel(open(FILENAME+"_XYZcenter.xyz", 'r').read(),␣
 ↪"xyz")
view.setStyle({'stick':{'colorscheme':'default'}},␣
 ↪viewer=(0,1))

view.addModel(open("czasteczkaH.xyz", 'r').read(), "xyz")
view.setStyle({'model':1}, {'sphere':{'scale': 0.35}},␣
 ↪viewer=(0,2))

licznik =0
with open(FILENAME+"_XYZcenter.xyz", 'r') as plik:
    linie = plik.readlines()   #
```

```
    for linia in linie[2:]:  #
        licznik += 1
        podzielone = linia.strip().split()
        #print(str(licznik) + "  " +podzielone[0])
        #print(str(licznik)+ "__"+linia.strip())

        #print(listaH)
        element_do_sprawdzenia = licznik-1
        if listaH.count(element_do_sprawdzenia) > 0:
            view.addLabel(str(licznik) +"␣
 ↪"+podzielone[0],{'font':'sans-serif','fontSize':
 ↪16,'fontColor':'black','fontOpacity':2,'borderThickness':0.
 ↪0,'borderColor':'red','borderOpacity':0.0,'backgroundColor':
 ↪'black','backgroundOpacity':0.0,'position':{'x':
 ↪float(podzielone[1]),'y':float(podzielone[2]),'z':
 ↪float(podzielone[3])},'inFront':'true','showBackground':
 ↪'true'});
view.zoomTo()
view.render()
view.show()
view.write_html(FILENAME+'_czasteczkaALL.html')
print(listaH)
```

```
####### H
```

```
# Hydrogen
# ista atomow wodoru
listaH = []
for i in Y_RAMANmat.index:
    #print(i.strip().split("_")[2] )
    if(i.strip().split("_")[2]=="H"):
        #print(i.strip().split("_")[2])
        listaH.append(int(i.strip().split("_")[0])-1)
print("Lista atomow wodoru: "+ str([element + 1 for element in␣
  ↪listaH]))
print( Y_RAMANmat.index[listaH])
czasteczkaH = []
czasteczkaH.extend([(len(listaH)),"\n\n"] )

# + for i in listaH: str(i) )
file = open(FILENAME+"_XYZcenter.xyz", 'r')
lista_linii = file.readlines()
file.close()
for i in listaH:
    czasteczkaH.append(lista_linii[i+2])
#print(czasteczkaH)


with open('czasteczkaH.xyz', 'w') as file:
    for element in czasteczkaH:
        file.write(str(element) + '')


view = py3Dmol.view(width=650, height=650)
view.setStyle({'sphere':{}}, viewer=(0,2))
view.addModel(open(FILENAME+"_XYZcenter.xyz", 'r').read(),␣
  ↪"xyz")
view.setStyle({'stick':{'colorscheme':'default'}},␣
  ↪viewer=(0,1))
```

```
view.addModel(open("czasteczkaH.xyz", 'r').read(), "xyz")
view.setStyle({'model':1}, {'sphere':{'scale': 0.35}},␣
↪viewer=(0,2))

licznik =0
with open(FILENAME+"_XYZcenter.xyz", 'r') as plik:
    linie = plik.readlines()  # Wczytaj wszystkie linie do␣
↪listy
    for linia in linie[2:]:  # Pomiń pierwsze dwie linie
        licznik += 1
        podzielone = linia.strip().split()
        #print(str(licznik) + "  " +podzielone[0])
        #print(str(licznik)+ "__"+linia.strip())

        #print(listaH)
        element_do_sprawdzenia = licznik-1
        if listaH.count(element_do_sprawdzenia) > 0:
            view.addLabel(str(licznik) +"␣
↪"+podzielone[0],{'font':'sans-serif','fontSize':
↪16,'fontColor':'black','fontOpacity':2,'borderThickness':0.
↪0,'borderColor':'red','borderOpacity':0.0,'backgroundColor':
↪'black','backgroundOpacity':0.0,'position':{'x':
↪float(podzielone[1]),'y':float(podzielone[2]),'z':
↪float(podzielone[3])},'inFront':'true','showBackground':
↪'true'});
view.zoomTo()
view.render()
view.show()
view.write_html(FILENAME+'_czasteczkaH.html')



####### Si

listaH = []
```

```
for i in Y_RAMANmat.index:
    #print(i.strip().split("_")[2] )
    if(i.strip().split("_")[2]=="Si"):
        #print(i.strip().split("_")[2])
        listaH.append(int(i.strip().split("_")[0])-1)
print("Lista atomow krzemu: "+ str([element + 1 for element in␣
 ↪listaH]))
print( Y_RAMANmat.index[listaH])
czasteczkaH = []
czasteczkaH.extend([(len(listaH)),"\n\n"] )

# + for i in listaH: str(i) )
file = open(FILENAME+"_XYZcenter.xyz", 'r')
lista_linii = file.readlines()
file.close()
for i in listaH:
    czasteczkaH.append(lista_linii[i+2])
#print(czasteczkaH)


with open('czasteczkaSi.xyz', 'w') as file:
    for element in czasteczkaH:
        file.write(str(element) + '')


view = py3Dmol.view(width=650, height=650)
view.setStyle({'sphere':{}}, viewer=(0,2))
view.addModel(open(FILENAME+"_XYZcenter.xyz", 'r').read(),␣
 ↪"xyz")
view.setStyle({'stick':{'colorscheme':'default'}},␣
 ↪viewer=(0,1))

view.addModel(open("czasteczkaSi.xyz", 'r').read(), "xyz")
view.setStyle({'model':1}, {'sphere':{'scale': 0.35}},␣
 ↪viewer=(0,2))
```

```
licznik =0
with open(FILENAME+"_XYZcenter.xyz", 'r') as plik:
    linie = plik.readlines()   #
    for linia in linie[2:]:   #
        licznik += 1
        podzielone = linia.strip().split()
        #print(str(licznik) + "   " +podzielone[0])
        #print(str(licznik)+ "__"+linia.strip())

        #print(listaH)
        element_do_sprawdzenia = licznik-1
        if listaH.count(element_do_sprawdzenia) > 0:
            view.addLabel(str(licznik) +"␣
 ↪"+podzielone[0],{'font':'sans-serif','fontSize':
 ↪18,'fontColor':'black','fontOpacity':2,'borderThickness':0.
 ↪0,'borderColor':'red','borderOpacity':0.0,'backgroundColor':
 ↪'black','backgroundOpacity':0.0,'position':{'x':
 ↪float(podzielone[1]),'y':float(podzielone[2]),'z':
 ↪float(podzielone[3])},'inFront':'true','showBackground':
 ↪'true'});
view.zoomTo()
view.render()
view.show()
view.write_html(FILENAME+'_czasteczkaSi.html')


####### O
# t
# i
listaH = []
for i in Y_RAMANmat.index:
    #print(i.strip().split("_")[2] )
    if(i.strip().split("_")[2]=="O"):
        #print(i.strip().split("_")[2])
```

```
        listaH.append(int(i.strip().split("_")[0])-1)
print("Oxygen: "+ str([element + 1 for element in listaH]))
print( Y_RAMANmat.index[listaH])
czasteczkaH = []
czasteczkaH.extend([(len(listaH)),"\n\n"] )

# + for i in listaH: str(i) )
file = open(FILENAME+"_XYZcenter.xyz", 'r')
lista_linii = file.readlines()
file.close()
for i in listaH:
    czasteczkaH.append(lista_linii[i+2])
#print(czasteczkaH)


with open('czasteczkaO.xyz', 'w') as file:
    for element in czasteczkaH:
        file.write(str(element) + '')



view = py3Dmol.view(width=650, height=650)
view.setStyle({'sphere':{}}, viewer=(0,2))
view.addModel(open(FILENAME+"_XYZcenter.xyz", 'r').read(),␣
 ↪"xyz")
view.setStyle({'stick':{'colorscheme':'default'}},␣
 ↪viewer=(0,1))

view.addModel(open("czasteczkaO.xyz", 'r').read(), "xyz")
view.setStyle({'model':1}, {'sphere':{'scale': 0.35}},␣
 ↪viewer=(0,2))

licznik =0
with open(FILENAME+"_XYZcenter.xyz", 'r') as plik:
    linie = plik.readlines()  # Wczytaj wszystkie linie do␣
 ↪listy
```

```
    for linia in linie[2:]:  # Pomiń pierwsze dwie linie
        licznik += 1
        podzielone = linia.strip().split()
        #print(str(licznik) + "   " +podzielone[0])
        #print(str(licznik)+ "__"+linia.strip())

        #print(listaH)
        element_do_sprawdzenia = licznik-1
        if listaH.count(element_do_sprawdzenia) > 0:
            view.addLabel(str(licznik) +"␣
 ↪"+podzielone[0],{'font':'sans-serif','fontSize':
 ↪18,'fontColor':'black','fontOpacity':2,'borderThickness':0.
 ↪0,'borderColor':'red','borderOpacity':0.0,'backgroundColor':
 ↪'black','backgroundOpacity':0.0,'position':{'x':
 ↪float(podzielone[1]),'y':float(podzielone[2]),'z':
 ↪float(podzielone[3])},'inFront':'true','showBackground':
 ↪'true'});
view.zoomTo()
view.render()
view.show()
view.write_html(FILENAME+'_czasteczka0.html')




####### C

listaH = []
for i in Y_RAMANmat.index:
    #print(i.strip().split("_")[2] )
    if(i.strip().split("_")[2]=="C"):
        #print(i.strip().split("_")[2])
        listaH.append(int(i.strip().split("_")[0])-1)
print("Carbon: "+ str([element + 1 for element in listaH]))
print( Y_RAMANmat.index[listaH])
```

```
czasteczkaH = []
czasteczkaH.extend([(len(listaH)),"\n\n"] )

# + for i in listaH: str(i) )
file = open(FILENAME+"_XYZcenter.xyz", 'r')
lista_linii = file.readlines()
file.close()
for i in listaH:
    czasteczkaH.append(lista_linii[i+2])
#print(czasteczkaH)


with open('czasteczkaC.xyz', 'w') as file:
    for element in czasteczkaH:
        file.write(str(element) + '')


view = py3Dmol.view(width=650, height=650)
view.setStyle({'sphere':{}}, viewer=(0,2))
view.addModel(open(FILENAME+"_XYZcenter.xyz", 'r').read(), "xyz")
view.setStyle({'stick':{'colorscheme':'default'}}, viewer=(0,1))

view.addModel(open("czasteczkaC.xyz", 'r').read(), "xyz")
view.setStyle({'model':1}, {'sphere':{'scale': 0.35}}, viewer=(0,2))

licznik =0
with open(FILENAME+"_XYZcenter.xyz", 'r') as plik:
    linie = plik.readlines()   #
    for linia in linie[2:]:   #
        licznik += 1
        podzielone = linia.strip().split()
        #print(str(licznik) + "  " +podzielone[0])
```

```
        #print(str(licznik)+ "__"+linia.strip())

        #print(listaH)
        element_do_sprawdzenia = licznik-1
        if listaH.count(element_do_sprawdzenia) > 0:
            view.addLabel(str(licznik) +"␣
 ↪"+podzielone[0],{'font':'sans-serif','fontSize':
 ↪18,'fontColor':'black','fontOpacity':2,'borderThickness':0.
 ↪0,'borderColor':'red','borderOpacity':0.0,'backgroundColor':
 ↪'black','backgroundOpacity':0.0,'position':{'x':
 ↪float(podzielone[1]),'y':float(podzielone[2]),'z':
 ↪float(podzielone[3])},'inFront':'true','showBackground':
 ↪'true'});
view.zoomTo()
view.render()
view.show()
view.write_html(FILENAME+'_czasteczkaC.html')


#######
#
#
listaH = []
for i in Y_RAMANmat.index:
    #print(i.strip().split("_")[2] )
    if(i.strip().split("_")[2]=="H"):
        #print("")
        smiec = 1
    else:
        if(i.strip().split("_")[2]=="O"):
            smiec = 1
        else:
            if(i.strip().split("_")[2]=="Si"):
                smiec = 1
            else:
```

```
                if(i.strip().split("_")[2]=="C"):
                    smiec = 1
                else:
                    listaH.append(int(i.strip().
 ↪split("_")[0])-1)
print("Pozostałe atomy: "+ str([element + 1 for element in␣
 ↪listaH]))
print( Y_RAMANmat.index[listaH])
czasteczkaH = []
czasteczkaH.extend([(len(listaH)),"\n\n"] )

# + for i in listaH: str(i) )
file = open(FILENAME+"_XYZcenter.xyz", 'r')
lista_linii = file.readlines()
file.close()
for i in listaH:
    czasteczkaH.append(lista_linii[i+2])
#print(czasteczkaH)


with open('czasteczkaPALL.xyz', 'w') as file:
    for element in czasteczkaH:
        file.write(str(element) + '')


view = py3Dmol.view(width=650, height=650)
view.setStyle({'sphere':{}}, viewer=(0,2))
view.addModel(open(FILENAME+"_XYZcenter.xyz", 'r').read(),␣
 ↪"xyz")
view.setStyle({'stick':{'colorscheme':'default'}},␣
 ↪viewer=(0,1))

view.addModel(open("czasteczkaPALL.xyz", 'r').read(), "xyz")
view.setStyle({'model':1}, {'sphere':{'scale': 0.35}},␣
 ↪viewer=(0,2))
```

```
licznik =0
with open(FILENAME+"_XYZcenter.xyz", 'r') as plik:
    linie = plik.readlines()   #
    for linia in linie[2:]:   #
        licznik += 1
        podzielone = linia.strip().split()
        #print(str(licznik) + "   " +podzielone[0])
        #print(str(licznik)+ "__"+linia.strip())

        #print(listaH)
        element_do_sprawdzenia = licznik-1
        if listaH.count(element_do_sprawdzenia) > 0:
            view.addLabel(str(licznik) +"␣
 ↪"+podzielone[0],{'font':'sans-serif','fontSize':
 ↪18,'fontColor':'black','fontOpacity':2,'borderThickness':0.
 ↪0,'borderColor':'red','borderOpacity':0.0,'backgroundColor':
 ↪'black','backgroundOpacity':0.0,'position':{'x':
 ↪float(podzielone[1]),'y':float(podzielone[2]),'z':
 ↪float(podzielone[3])},'inFront':'true','showBackground':
 ↪'true'});
view.zoomTo()
view.render()
view.show()
view.write_html(FILENAME+'_czasteczkaPALL.html')
listaH = []
```

```
gv5_SBA15_Dh2o_D4_173_50_FREQ_2_Cu_4H2O_FREQ_17_FREQ
Lista wszystkich atomów: [1, 2, 3, 4, 5, 6, 7, 8, 9, 10, 11,␣
 ↪12, 13, 14, 15, 16,
17, 18, 19, 20, 21, 22, 23, 24, 25, 26, 27, 28, 29, 30, 31, 32,␣
 ↪33, 34, 35, 36,
37, 38, 39, 40, 41, 42, 43, 44, 45, 46, 47, 48, 49, 50, 51, 52,␣
 ↪53, 54, 55, 56,
57, 58, 59, 60, 61, 62, 63, 64, 65, 66, 67, 68, 69, 70, 71, 72,␣
 ↪73, 74, 75, 76,
```

```
77, 78, 79, 80, 81, 82, 83, 84, 85, 86, 87, 88, 89, 90, 91, 92,␣
 ↪93, 94, 95, 96,
97, 98, 99, 100, 101, 102, 103, 104, 105, 106, 107, 108, 109,␣
 ↪110, 111, 112,
113, 114, 115, 116, 117, 118, 119, 120, 121, 122, 123, 124,␣
 ↪125, 126, 127, 128,
129, 130, 131, 132, 133, 134, 135]
Index(['1__O', '2__O', '3__Si', '4__Si', '5__O', '6__Si',␣
 ↪'7__O', '8__O',
       '9__Si', '10__O',
       ...
       '126__O', '127__H', '128__O', '129__H', '130__O',␣
 ↪'131__H', '132__H',
       '133__H', '134__H', '135__H'],
      dtype='object', name='Unnamed: 0', length=135)
```

```
[0, 1, 2, 3, 4, 5, 6, 7, 8, 9, 10, 11, 12, 13, 14, 15, 16, 17,␣
 ↪18, 19, 20, 21,
22, 23, 24, 25, 26, 27, 28, 29, 30, 31, 32, 33, 34, 35, 36, 37,␣
 ↪38, 39, 40, 41,
42, 43, 44, 45, 46, 47, 48, 49, 50, 51, 52, 53, 54, 55, 56, 57,␣
 ↪58, 59, 60, 61,
62, 63, 64, 65, 66, 67, 68, 69, 70, 71, 72, 73, 74, 75, 76, 77,␣
 ↪78, 79, 80, 81,
82, 83, 84, 85, 86, 87, 88, 89, 90, 91, 92, 93, 94, 95, 96, 97,␣
 ↪98, 99, 100,
101, 102, 103, 104, 105, 106, 107, 108, 109, 110, 111, 112,␣
 ↪113, 114, 115, 116,
117, 118, 119, 120, 121, 122, 123, 124, 125, 126, 127, 128,␣
 ↪129, 130, 131, 132,
133, 134]
Lista atomow wodoru: [25, 26, 28, 29, 47, 48, 49, 51, 52, 53,␣
 ↪55, 56, 57, 60,
61, 62, 64, 65, 66, 68, 69, 70, 74, 75, 76, 78, 79, 80, 82, 83,␣
 ↪84, 86, 88, 90,
```

```
92, 94, 96, 98, 100, 102, 104, 106, 108, 110, 112, 114, 116,␣
 ↪118, 120, 121, 122,
125, 127, 129, 131, 132, 133, 134, 135]
Index(['25__H', '26__H', '28__H', '29__H', '47__H', '48__H',␣
 ↪'49__H', '51__H',
       '52__H', '53__H', '55__H', '56__H', '57__H', '60__H',␣
 ↪'61__H', '62__H',
       '64__H', '65__H', '66__H', '68__H', '69__H', '70__H',␣
 ↪'74__H', '75__H',
       '76__H', '78__H', '79__H', '80__H', '82__H', '83__H',␣
 ↪'84__H', '86__H',
       '88__H', '90__H', '92__H', '94__H', '96__H', '98__H',␣
 ↪'100__H',
       '102__H', '104__H', '106__H', '108__H', '110__H',␣
 ↪'112__H', '114__H',
       '116__H', '118__H', '120__H', '121__H', '122__H',␣
 ↪'125__H', '127__H',
       '129__H', '131__H', '132__H', '133__H', '134__H',␣
 ↪'135__H'],
      dtype='object', name='Unnamed: 0')
Lista atomow krzemu: [3, 4, 6, 9, 12, 14, 17, 19, 21, 35, 37,␣
 ↪38, 41, 42, 45,
58, 72]
Index(['3__Si', '4__Si', '6__Si', '9__Si', '12__Si', '14__Si',␣
 ↪'17__Si',
       '19__Si', '21__Si', '35__Si', '37__Si', '38__Si',␣
 ↪'41__Si', '42__Si',
       '45__Si', '58__Si', '72__Si'],
      dtype='object', name='Unnamed: 0')
Lista atomow tlenu: [1, 2, 5, 7, 8, 10, 11, 13, 15, 16, 18, 20,␣
 ↪30, 31, 32, 33,
34, 36, 39, 40, 43, 44, 71, 85, 87, 89, 91, 93, 95, 97, 99,␣
 ↪101, 103, 105, 107,
109, 111, 113, 115, 117, 119, 124, 126, 128, 130]
```

```
Index(['1__O', '2__O', '5__O', '7__O', '8__O', '10__O',␣
↪'11__O', '13__O',
       '15__O', '16__O', '18__O', '20__O', '30__O', '31__O',␣
↪'32__O', '33__O',
       '34__O', '36__O', '39__O', '40__O', '43__O', '44__O',␣
↪'71__O', '85__O',
       '87__O', '89__O', '91__O', '93__O', '95__O', '97__O',␣
↪'99__O', '101__O',
       '103__O', '105__O', '107__O', '109__O', '111__O',␣
↪'113__O', '115__O',
       '117__O', '119__O', '124__O', '126__O', '128__O',␣
↪'130__O'],
      dtype='object', name='Unnamed: 0')
Lista atomow węgla: [22, 23, 24, 46, 50, 54, 59, 63, 67, 73,␣
↪77, 81]
Index(['22__C', '23__C', '24__C', '46__C', '50__C', '54__C',␣
↪'59__C', '63__C',
       '67__C', '73__C', '77__C', '81__C'],
      dtype='object', name='Unnamed: 0')
Pozostałe atomy: [27, 123]
Index(['27__P', '123__Cu'], dtype='object', name='Unnamed: 0')
```

[ ]:

```

```

[13]:

```
FILENAMElog
```

[13]: 'gv5_SBA15_Dh2o_D4_173_50_FREQ_2_Cu_4H2O_FREQ_17_FREQ.log'

[14]:

```
FILENAMElog = NAZWAPLIKU
FILENAME = FILENAMElog.replace(".log", "")
print(FILENAME)
```

```
Y_RAMANmat = pd.read_csv(FILENAME+"_decomposition_RAMAN.csv",␣
  ↪header=0, dtype={'Unnamed: 0':str})
Y_RAMANmat.set_index('Unnamed: 0', inplace=True)
Y_IRmat = pd.read_csv(FILENAME+"_decomposition_IR.csv",␣
  ↪header=0, dtype={'Unnamed: 0':str})
Y_IRmat.set_index('Unnamed: 0', inplace=True)


x = np.linspace(0, 4200,42000)
sumaGrupaRAMAN = pd.DataFrame(x).T#3
sumaGrupaIR = pd.DataFrame(x).T#3



Y_RAMANmatALL = pd.read_csv(FILENAME+"_ALL_RAMAN.csv",␣
  ↪header=0, dtype={'Unnamed: 0':str})
#sumaGrupaRAMAN
sumaGrupaRAMANindex =["x", "x-factor 0.9750", "x-factor 0.
  ↪9480" ,"x-factor 0.9462", "Spectrum All"]   # Publikacje AI␣
  ↪Raman, biocinal, Sara
sumaGrupaRAMAN = pd.concat([sumaGrupaRAMAN, pd.DataFrame(x*0.
  ↪9750).T], ignore_index=True)
sumaGrupaRAMAN = pd.concat([sumaGrupaRAMAN, pd.DataFrame(x*0.
  ↪9480).T], ignore_index=True)
sumaGrupaRAMAN = pd.concat([sumaGrupaRAMAN, pd.DataFrame(x*0.
  ↪9480).T], ignore_index=True)
sumaGrupaRAMAN = pd.concat([sumaGrupaRAMAN, pd.
  ↪DataFrame(Y_RAMANmatALL.iloc[:,1]).T ], ignore_index=True)



Y_IRmatALL = pd.read_csv(FILENAME+"_ALL_IR.csv", header=0,␣
  ↪dtype={'Unnamed: 0':str})
#sumaGrupaRAMAN
```

```
sumaGrupaIRindex =["x", "x-factor 0.9750", "x-factor 0.9480"␣
↪,"x-factor 0.9462", "Spectrum All"]    # Publikacje AI␣
↪Raman, biocinal, Sara
sumaGrupaIR = pd.concat([sumaGrupaIR, pd.DataFrame(x*0.9750).
↪T], ignore_index=True)
sumaGrupaIR = pd.concat([sumaGrupaIR, pd.DataFrame(x*0.9480).
↪T], ignore_index=True)
sumaGrupaIR = pd.concat([sumaGrupaIR, pd.DataFrame(x*0.9480).
↪T], ignore_index=True)
sumaGrupaIR = pd.concat([sumaGrupaIR, pd.DataFrame(Y_IRmatALL.
↪iloc[:,1]).T ], ignore_index=True)
```

gv5_SBA15_Dh2o_D4_173_50_FREQ_2_Cu_4H2O_FREQ_17_FREQ

[ ]:

```

```

[15]:

```
#########################

def exportuje_grupe(GRUPA, ATOMS, Y_RAMANmat=Y_RAMANmat ): #,␣
↪sumaGrupaRAMAN=sumaGrupaRAMAN,␣
↪sumaGrupaRAMANindex=sumaGrupaRAMANindex,␣
↪sumaGrupaIR=sumaGrupaIR):
    global sumaGrupaRAMAN
    global sumaGrupaRAMANindex
    global sumaGrupaIRindex
    global sumaGrupaIR
    # RAMAN
    plt.figure(figsize=(20,6))
    plt.subplot(2, 1, 1)
    sumaGrupa = np.array(Y_RAMANmat.iloc[0,:]*0)
    for i in ATOMS:
        plt.plot(x, Y_RAMANmat.iloc[i-1,:], label=Y_RAMANmat.
↪index[i-1])        # i-1
        #print("testuje")
        #print(Y_RAMANmat.index[i])                              ␣
↪                #
```

```
        #print("testuje")
        print(Y_RAMANmat.index[i-1])                                    ␣
↪                   #
        sumaGrupa = sumaGrupa + np.array(Y_RAMANmat.iloc[i-1,:
↪])
    ### Y_RAMANmat.iloc[ATOMS2].sum() #
    plt.title(" ")
    plt.legend()
    #plt.show()
    plt.subplot(2, 1, 2)
    #plt.figure(figsize=(20,6))
    plt.title(GRUPA[0])
    plt.plot(x, sumaGrupa, label=GRUPA)
    plt.legend(prop={'size': 6})
    #sumaGrupa= sumaGrupa.reset_index(drop=True)
    sumaGrupaRAMAN = pd.concat([sumaGrupaRAMAN, pd.
↪DataFrame(sumaGrupa).T])
    sumaGrupaRAMANindex.append(GRUPA)
    plt.savefig(FILENAME+"_____"+str(GRUPA[0])+"_RAMAN.png")
    plt.savefig(FILENAME+"_____"+str(GRUPA[0])+"_RAMAN.pdf")


    #IR
    plt.figure(figsize=(20,6))
    plt.subplot(2, 1, 1)
    sumaGrupa = Y_IRmat.iloc[0,:]*0
    maxOY = 0
    for i in ATOMS:
        plt.plot(x, Y_IRmat.iloc[i-1,:], label=Y_IRmat.
↪index[i-1])
        maxOY = max(max(Y_IRmat.iloc[i-1,:]), maxOY)
        #print(Y_RAMANmat.index[i-1])
        sumaGrupa = sumaGrupa + Y_IRmat.iloc[i-1,:]
    plt.title(" ")
    plt.legend()
```

```
    plt.ylim(maxOY+1,-1)
    plt.xlim(4220,-20)
    #plt.show()
    #plt.figure(figsize=(20,6))
    plt.subplot(2, 1, 2)
    plt.title(GRUPA[0])
    plt.plot(x, sumaGrupa, label=GRUPA)
    plt.legend(prop={'size': 6})
    plt.ylim(max(sumaGrupa)+1,-1)
    plt.xlim(4220,-20)
    sumaGrupa= sumaGrupa.reset_index(drop=True)
    sumaGrupaIR = pd.concat([sumaGrupaIR, pd.
 ↪DataFrame(sumaGrupa).T])
    sumaGrupaIRindex.append(GRUPA)
    plt.savefig(FILENAME+"_____"+str(GRUPA[0])+"_IR.png")
    plt.savefig(FILENAME+"_____"+str(GRUPA[0])+"_IR.pdf")

    return sumaGrupa, sumaGrupaRAMAN, sumaGrupaRAMANindex,␣
 ↪sumaGrupaIRindex, sumaGrupaIR

############################
```

[16]:
```
def wizualizacja_czasteczki(ATOMS, GRUPA):
    czasteczkaH = []
    czasteczkaH.extend([(len(ATOMS)),"\n\n"] )

    # + for i in listaH: str(i) )
    file = open(FILENAME+"_XYZcenter.xyz", 'r')
    lista_linii = file.readlines()
    lista_linii.append("\n")
    file.close()
    for i in ATOMS:
        if not lista_linii[i+1].endswith('\n'):
            #print("aaa")
```

```
            lista_linii[i+1] = lista_linii[i+1].rstrip() +␣
↪'\n'   #   \n
        czasteczkaH.append(lista_linii[i+1])
        #print(lista_linii[i+1])
    #print(czasteczkaH)


    with open('czasteczka_TEMP1.xyz', 'w') as file:
        for element in czasteczkaH:
            file.write(str(element) + '')
    view = py3Dmol.view(width=800, height=800)
    view.setStyle({'sphere':{}}, viewer=(0,2))
    view.addModel(open(FILENAME+"_XYZcenter.xyz", 'r').read(),␣
↪"xyz")
    view.setStyle({'stick':{'colorscheme':'default'}},␣
↪viewer=(0,1))

    view.addModel(open("czasteczka_TEMP1.xyz", 'r').read(),␣
↪"xyz")
    view.setStyle({'model':1}, {'sphere':{'scale': 0.35}},␣
↪viewer=(0,2))

    licznik =0
    with open(FILENAME+"_XYZcenter.xyz", 'r') as plik:
        linie = plik.readlines()   #
        for linia in linie[2:]:   #
            licznik += 1
            podzielone = linia.strip().split()
            #print(str(licznik) + "   " +podzielone[0])
            #print(str(licznik)+ "__"+linia.strip())

            #print(listaH)
            element_do_sprawdzenia = licznik
            if ATOMS.count(element_do_sprawdzenia) > 0:
```

```
                    view.addLabel(str(licznik) +"␣
↪"+podzielone[0],{'font':'sans-serif','fontSize':
↪18,'fontColor':'black','fontOpacity':2,'borderThickness':0.
↪0,'borderColor':'red','borderOpacity':0.0,'backgroundColor':
↪'black','backgroundOpacity':0.0,'position':{'x':
↪float(podzielone[1]),'y':float(podzielone[2]),'z':
↪float(podzielone[3])},'inFront':'true','showBackground':
↪'true'});
    view.zoomTo()
    view.render()
    view.show()
    view.
↪write_html(str(str(FILENAME)+str('_____')+str(GRUPA[0])+str('.
↪html') ))
```

[17]:
```
GRUPA = ["SiO"]
ATOMS = [1, 2, 5, 7, 8, 10, 11, 13,  16, 18, 20, 35, 36, 38,␣
↪41, 42,        3, 4, 6, 9, 12, 14, 17, 19, 21, 37, 39, 40,␣
↪43,  33,34  ]
ATOMS =␣
↪[68,74,70,64,66,78,62,54,58,48,56,15,52,50,80,72,60,76,46,45,44␣
↪   , 47, 49, 51, 53, 55, 57, 59, 61, 63, 65, 67, 69, 71,␣
↪73, 75, 77, 79, 81,    86, 84,85]
ATOMS = [27, 30, 31, 32]
ATOMS = [22, 23, 24, 25, 26, 28, 29, 82, 83]
ATOMS = [100,101]
ATOMS =␣
↪[85,87,89,91,93,95,97,99,101,103,105,107,109,101,111,113,115,117,119]
ATOMS = [ 46, 50, 54, 59, 63, 67, 73, 77, 81,    47,48, 49,␣
↪51, 52, 53, 55, 56, 57, 60, 61, 62, 64, 65, 66, 68, 69, 70,␣
↪74, 75, 76, 78, 79, 80, 82, 83, 84 ]
#ATOMS = [86, 88, 90, 92, 94, 96, 98, 100, 102, 104, 106, 108,␣
↪110, 112, 114, 116, 118, 120]
ATOMS = [ 125, 127, 129, 131, 132, 133, 134, 135]
print(sorted(ATOMS))
```

```
#exportuje_grupe(GRUPA = GRUPA, ATOMS = ATOMS,␣
 ↪Y_RAMANmat=Y_RAMANmat)#, sumaGrupaRAMAN=sumaGrupaRAMAN,␣
 ↪sumaGrupaRAMANindex=sumaGrupaRAMANindex,␣
 ↪sumaGrupaIR=sumaGrupaIR)
wizualizacja_czasteczki(GRUPA = GRUPA, ATOMS = ATOMS)
```

```
[125, 127, 129, 131, 132, 133, 134, 135]
```

[18]:
```
import time



#OK
GRUPA = ["SiO"]
ATOMS = [3, 4, 6, 9, 12, 14, 17, 19, 21, 35, 37, 38, 41, 42,  ␣
 ↪    1, 2, 5, 7, 8, 10, 11, 13, 15, 16, 18, 20,  33, 34, 36,␣
 ↪39, 40, 43, 44, 71]
print(sorted(ATOMS))
exportuje_grupe(GRUPA = GRUPA, ATOMS = ATOMS,␣
 ↪Y_RAMANmat=Y_RAMANmat)#, sumaGrupaRAMAN=sumaGrupaRAMAN,␣
 ↪sumaGrupaRAMANindex=sumaGrupaRAMANindex,␣
 ↪sumaGrupaIR=sumaGrupaIR)
wizualizacja_czasteczki(GRUPA = GRUPA, ATOMS = ATOMS)

#ok
GRUPA = ["-OH from SiOH"]
ATOMS =␣
 ↪[85,87,89,91,93,95,97,99,101,103,105,107,109,101,111,113,115,117,119␣
 ↪  , 86, 88, 90, 92, 94, 96, 98, 100, 102, 104, 106, 108,␣
 ↪110, 112, 114, 116, 118, 120]
print(sorted(ATOMS))
exportuje_grupe(GRUPA = GRUPA, ATOMS = ATOMS,␣
 ↪Y_RAMANmat=Y_RAMANmat)#, sumaGrupaRAMAN=sumaGrupaRAMAN,␣
 ↪sumaGrupaRAMANindex=sumaGrupaRAMANindex,␣
 ↪sumaGrupaIR=sumaGrupaIR)
wizualizacja_czasteczki(GRUPA = GRUPA, ATOMS = ATOMS)
```

```
#ok
GRUPA = ["O-Si(CH3)3"]
ATOMS = [14, 15,71    ,  45,58,72, 46, 50, 54, 59, 63, 67, 73,␣
 ↪77, 81,    47,48, 49, 51, 52, 53, 55, 56, 57, 60, 61, 62,␣
 ↪64, 65, 66, 68, 69, 70, 74, 75, 76, 78, 79, 80, 82, 83, 84]
print(sorted(ATOMS))
exportuje_grupe(GRUPA = GRUPA, ATOMS = ATOMS,␣
 ↪Y_RAMANmat=Y_RAMANmat)#, sumaGrupaRAMAN=sumaGrupaRAMAN,␣
 ↪sumaGrupaRAMANindex=sumaGrupaRAMANindex,␣
 ↪sumaGrupaIR=sumaGrupaIR)
wizualizacja_czasteczki(GRUPA = GRUPA, ATOMS = ATOMS)


GRUPA = ["CH2CH2CH2"]
ATOMS = [22, 23, 24,   25, 26, 28, 29, 121,122]
print(sorted(ATOMS))
exportuje_grupe(GRUPA = GRUPA, ATOMS = ATOMS,␣
 ↪Y_RAMANmat=Y_RAMANmat)#, sumaGrupaRAMAN=sumaGrupaRAMAN,␣
 ↪sumaGrupaRAMANindex=sumaGrupaRAMANindex,␣
 ↪sumaGrupaIR=sumaGrupaIR)
wizualizacja_czasteczki(GRUPA = GRUPA, ATOMS = ATOMS)


# OK
GRUPA = ["PO3Cu"]
ATOMS = [27, 123, 30, 31, 32]
print(sorted(ATOMS))
exportuje_grupe(GRUPA = GRUPA, ATOMS = ATOMS,␣
 ↪Y_RAMANmat=Y_RAMANmat)#, sumaGrupaRAMAN=sumaGrupaRAMAN,␣
 ↪sumaGrupaRAMANindex=sumaGrupaRAMANindex,␣
 ↪sumaGrupaIR=sumaGrupaIR)
wizualizacja_czasteczki(GRUPA = GRUPA, ATOMS = ATOMS)
```

```
#OK
GRUPA = ["H2O"]
ATOMS =[ 125, 127, 129, 131, 132, 133, 134, 135, ␣
 ↪124,130,126,128   ]
print(sorted(ATOMS))
exportuje_grupe(GRUPA = GRUPA, ATOMS = ATOMS,␣
 ↪Y_RAMANmat=Y_RAMANmat)#, sumaGrupaRAMAN=sumaGrupaRAMAN,␣
 ↪sumaGrupaRAMANindex=sumaGrupaRAMANindex,␣
 ↪sumaGrupaIR=sumaGrupaIR)
wizualizacja_czasteczki(GRUPA = GRUPA, ATOMS = ATOMS)



GRUPA = ["ALL-test"]
ATOMS = [1, 2, 3, 4, 5, 6, 7, 8, 9, 10, 11, 12, 13, 14, 15,␣
 ↪16, 17, 18, 19, 20, 21, 22, 23, 24, 25, 26, 27, 28, 29, 30,␣
 ↪31, 32, 33, 34, 35, 36, 37, 38, 39, 40, 41, 42, 43, 44, 45,␣
 ↪46, 47, 48, 49, 50, 51, 52, 53, 54, 55, 56, 57, 58, 59, 60,␣
 ↪61, 62, 63, 64, 65, 66, 67, 68, 69, 70, 71, 72, 73, 74, 75,␣
 ↪76, 77, 78, 79, 80, 81, 82, 83, 84, 85, 86, 87, 88, 89, 90,␣
 ↪91, 92, 93, 94, 95, 96, 97, 98, 99, 100, 101, 102, 103,␣
 ↪104, 105, 106, 107, 108, 109, 110, 111, 112, 113, 114, 115,␣
 ↪116, 117, 118, 119, 120, 121, 122, 123, 124, 125, 126, 127,␣
 ↪128, 129, 130, 131, 132, 133, 134, 135]
exportuje_grupe(GRUPA = GRUPA, ATOMS = ATOMS,␣
 ↪Y_RAMANmat=Y_RAMANmat)#, sumaGrupaRAMAN=sumaGrupaRAMAN,␣
 ↪sumaGrupaRAMANindex=sumaGrupaRAMANindex,␣
 ↪sumaGrupaIR=sumaGrupaIR)
wizualizacja_czasteczki(GRUPA = GRUPA, ATOMS = ATOMS),
```

```
[1, 2, 3, 4, 5, 6, 7, 8, 9, 10, 11, 12, 13, 14, 15, 16, 17, 18,␣
 ↪19, 20, 21, 33,
34, 35, 36, 37, 38, 39, 40, 41, 42, 43, 44, 71]
3__Si
```

```
4__Si
6__Si
9__Si
12__Si
14__Si
17__Si
19__Si
21__Si
35__Si
37__Si
38__Si
41__Si
42__Si
1__O
2__O
5__O
7__O
8__O
10__O
11__O
13__O
15__O
16__O
18__O
20__O
33__O
34__O
36__O
39__O
40__O
43__O
44__O
71__O
```

```
[85, 86, 87, 88, 89, 90, 91, 92, 93, 94, 95, 96, 97, 98, 99,␣
↪100, 101, 101, 102,
```

```
103, 104, 105, 106, 107, 108, 109, 110, 111, 112, 113, 114,␣
 ↪115, 116, 117, 118,
119, 120]
85__O
87__O
89__O
91__O
93__O
95__O
97__O
99__O
101__O
103__O
105__O
107__O
109__O
101__O
111__O
113__O
115__O
117__O
119__O
86__H
88__H
90__H
92__H
94__H
96__H
98__H
100__H
102__H
104__H
106__H
108__H
110__H
112__H
```

```
114__H
116__H
118__H
120__H
```

```
[14, 15, 45, 46, 47, 48, 49, 50, 51, 52, 53, 54, 55, 56, 57,␣
 ↪58, 59, 60, 61, 62,
63, 64, 65, 66, 67, 68, 69, 70, 71, 72, 73, 74, 75, 76, 77, 78,␣
 ↪79, 80, 81, 82,
83, 84]
14__Si
15__O
71__O
45__Si
58__Si
72__Si
46__C
50__C
54__C
59__C
63__C
67__C
73__C
77__C
81__C
47__H
48__H
49__H
51__H
52__H
53__H
55__H
56__H
57__H
60__H
61__H
```

62__H
64__H
65__H
66__H
68__H
69__H
70__H
74__H
75__H
76__H
78__H
79__H
80__H
82__H
83__H
84__H

[22, 23, 24, 25, 26, 28, 29, 121, 122]
22__C
23__C
24__C
25__H
26__H
28__H
29__H
121__H
122__H

[27, 30, 31, 32, 123]
27__P
123__Cu
30__O
31__O
32__O

[124, 125, 126, 127, 128, 129, 130, 131, 132, 133, 134, 135]
125__H
127__H

129__H
131__H
132__H
133__H
134__H
135__H
124__O
130__O
126__O
128__O

1__O
2__O
3__Si
4__Si
5__O
6__Si
7__O
8__O
9__Si
10__O
11__O
12__Si
13__O
14__Si
15__O
16__O
17__Si
18__O
19__Si
20__O
21__Si
22__C
23__C
24__C
25__H
26__H

27__P
28__H
29__H
30__O
31__O
32__O
33__O
34__O
35__Si
36__O
37__Si
38__Si
39__O
40__O
41__Si
42__Si
43__O
44__O
45__Si
46__C
47__H
48__H
49__H
50__C
51__H
52__H
53__H
54__C
55__H
56__H
57__H
58__Si
59__C
60__H
61__H
62__H

63__C
64__H
65__H
66__H
67__C
68__H
69__H
70__H
71__O
72__Si
73__C
74__H
75__H
76__H
77__C
78__H
79__H
80__H
81__C
82__H
83__H
84__H
85__O
86__H
87__O
88__H
89__O
90__H
91__O
92__H
93__O
94__H
95__O
96__H
97__O
98__H

99__O
100__H
101__O
102__H
103__O
104__H
105__O
106__H
107__O
108__H
109__O
110__H
111__O
112__H
113__O
114__H
115__O
116__H
117__O
118__H
119__O
120__H
121__H
122__H
123__Cu
124__O
125__H
126__O
127__H
128__O
129__H
130__O
131__H
132__H
133__H
134__H

135__H

[18]: (None,)

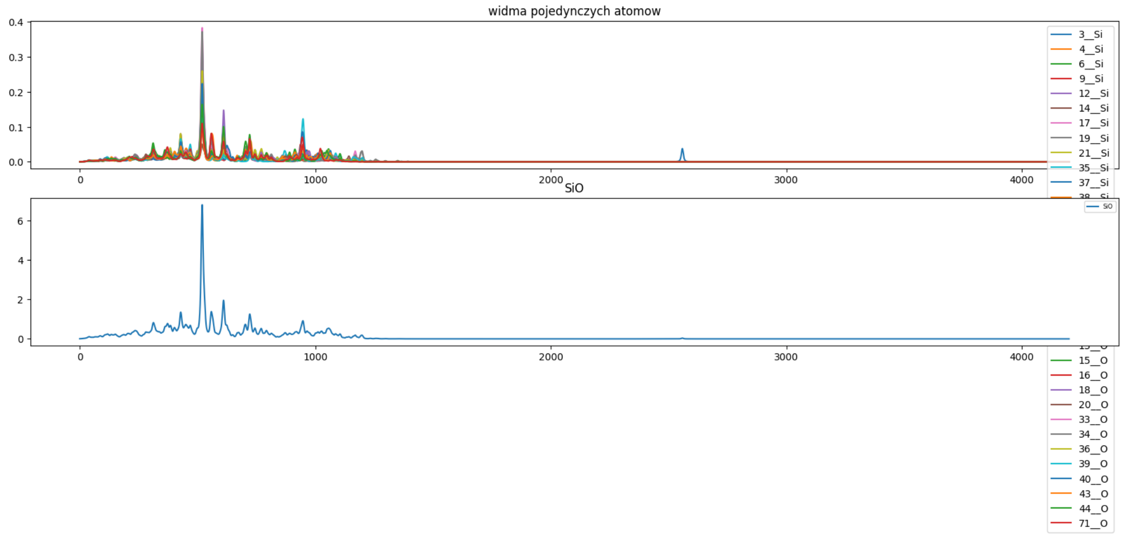
widma pojedynczych atomow
0.4
0.3
0.2
0.1
0.0
0
1000
2000
3000
4000
3__Si
4__Si
6__Si
9__Si
12__Si
14__Si
17__Si
19__Si
21__Si
35__Si
37__Si
SiO
6
4
2
0
15__O
16__O
18__O
20__O
33__O
34__O
36__O
39__O
40__O
43__O
44__O
71__O

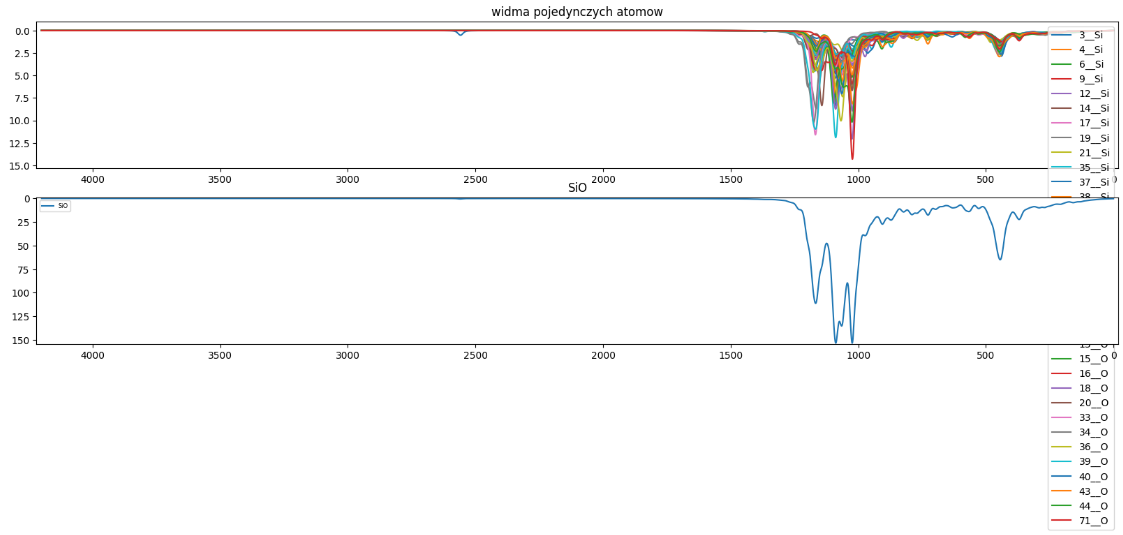
widma pojedynczych atomow
0.0
2.5
5.0
7.5
10.0
12.5
15.0
4000
3500
3000
2500
2000
1500
1000
500
3__Si
4__Si
6__Si
9__Si
12__Si
14__Si
17__Si
19__Si
21__Si
35__Si
37__Si
SiO
0
25
50
75
100
125
150
15__O
16__O
18__O
20__O
33__O
34__O
36__O
39__O
40__O
43__O
44__O
71__O

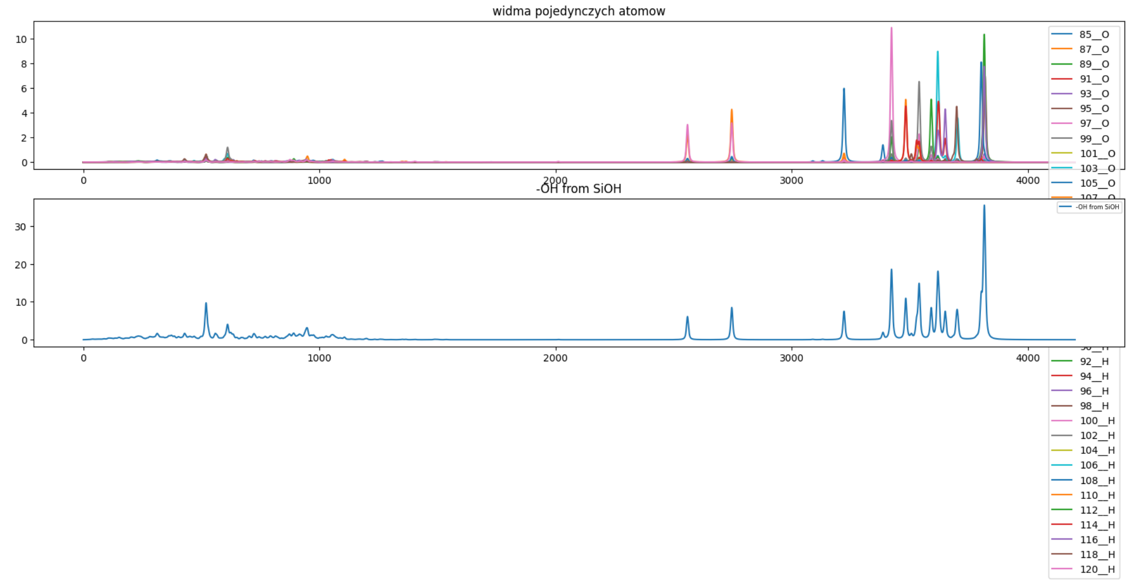
widma pojedynczych atomow
-OH from SiOH
85__O
87__O
89__O
91__O
93__O
95__O
97__O
99__O
101__O
103__O
105__O
92__H
94__H
96__H
98__H
100__H
102__H
104__H
106__H
108__H
110__H
112__H
114__H
116__H
118__H
120__H

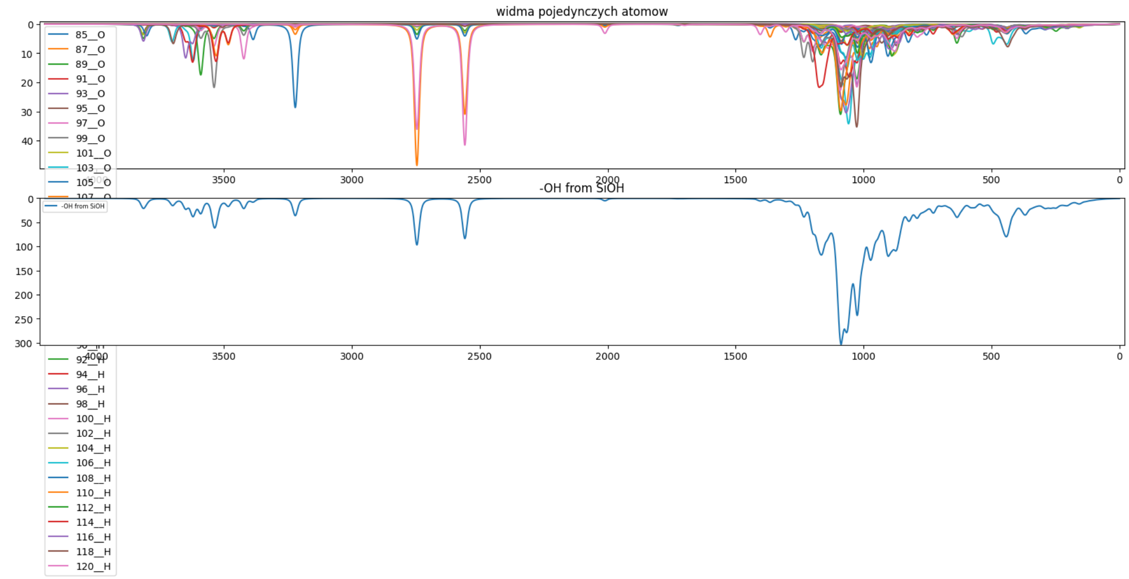
widma pojedynczych atomow
-OH from SiOH
85__O
87__O
89__O
91__O
93__O
95__O
97__O
99__O
101__O
103__O
105__O
92__H
94__H
96__H
98__H
100__H
102__H
104__H
106__H
108__H
110__H
112__H
114__H
116__H
118__H
120__H

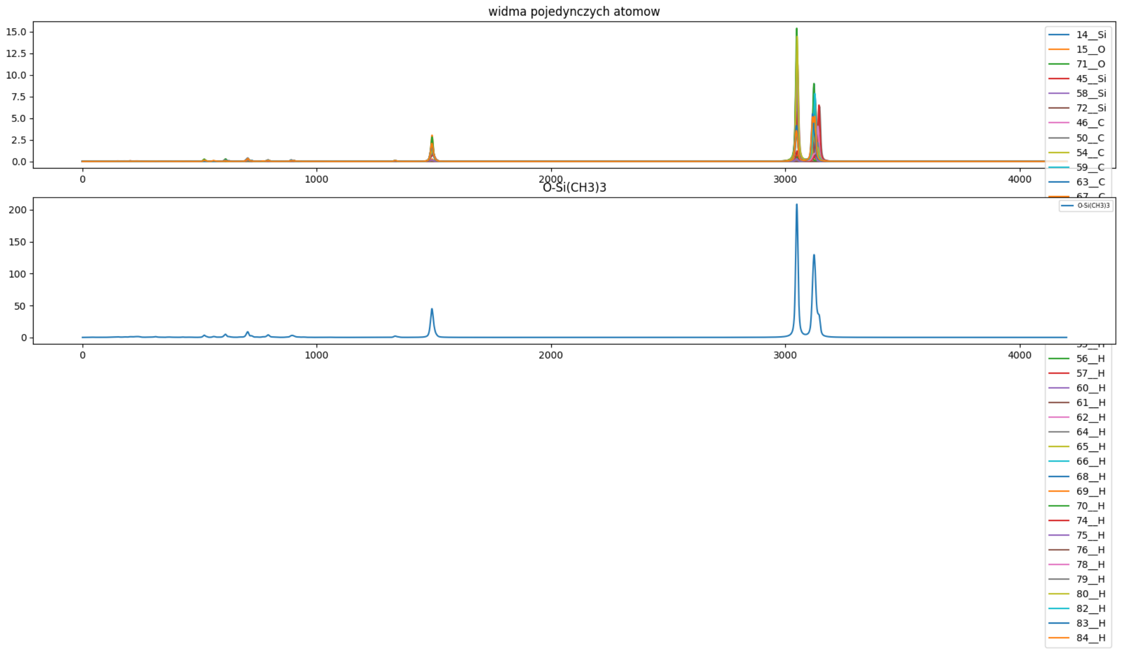
widma pojedynczych atomow
14__Si
15__O
71__O
45__Si
58__Si
72__Si
46__C
50__C
54__C
59__C
63__C
O-Si(CH3)3
56__H
57__H
60__H
61__H
62__H
64__H
65__H
66__H
68__H
69__H
70__H
74__H
75__H
76__H
78__H
79__H
80__H
82__H
83__H
84__H

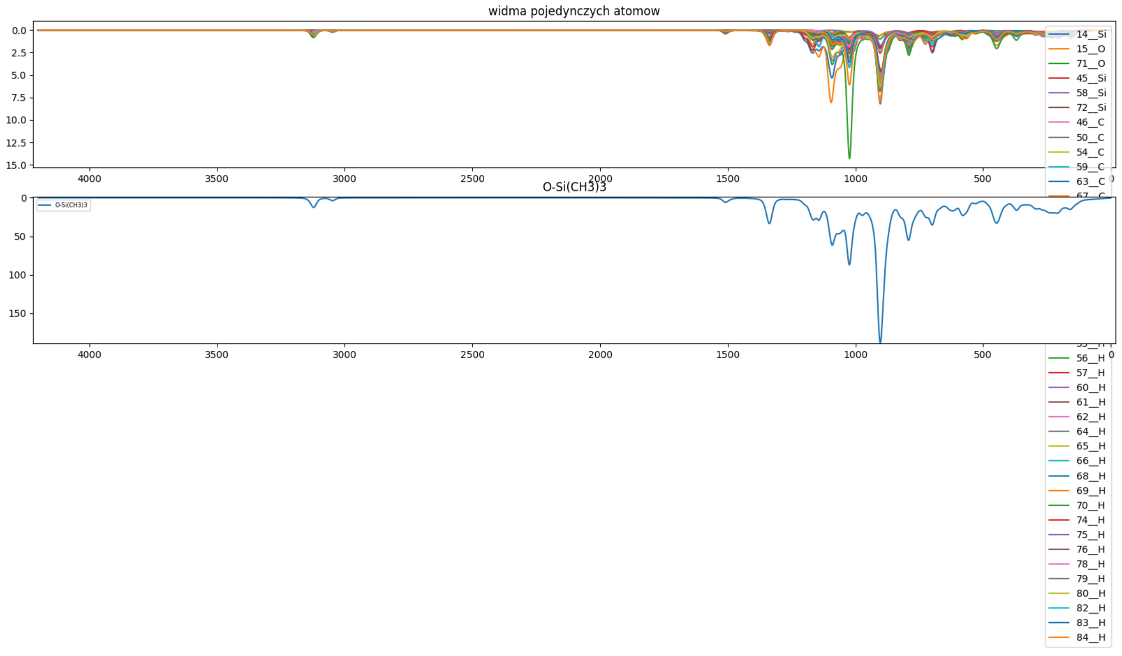
widma pojedynczych atomow
14__Si
15__O
71__O
45__Si
58__Si
72__Si
46__C
50__C
54__C
59__C
63__C
O-Si(CH3)3
56__H
57__H
60__H
61__H
62__H
64__H
65__H
66__H
68__H
69__H
70__H
74__H
75__H
76__H
78__H
79__H
80__H
82__H
83__H
84__H

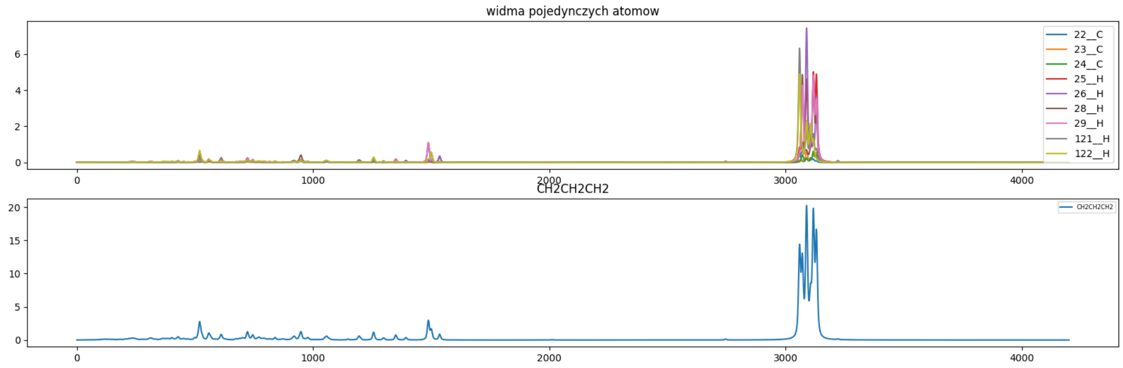
widma pojedynczych atomow
22__C
23__C
24__C
25__H
26__H
28__H
29__H
121__H
122__H
CH2CH2CH2

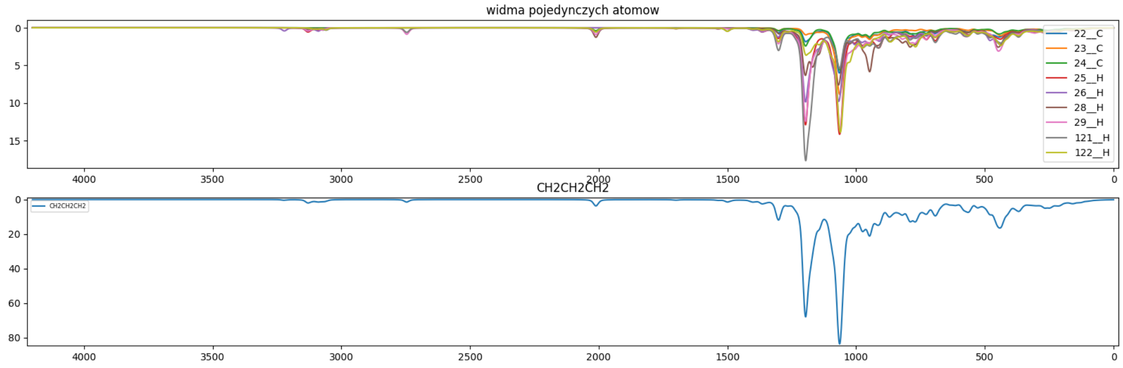
widma pojedynczych atomow
22__C
23__C
24__C
25__H
26__H
28__H
29__H
121__H
122__H
CH2CH2CH2

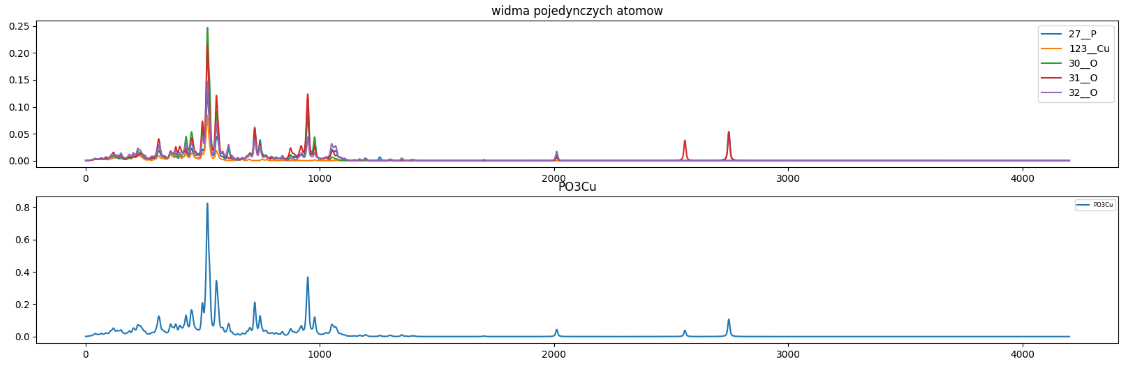
widma pojedynczych atomow
27__P
123__Cu
30__O
31__O
32__O
PO3Cu

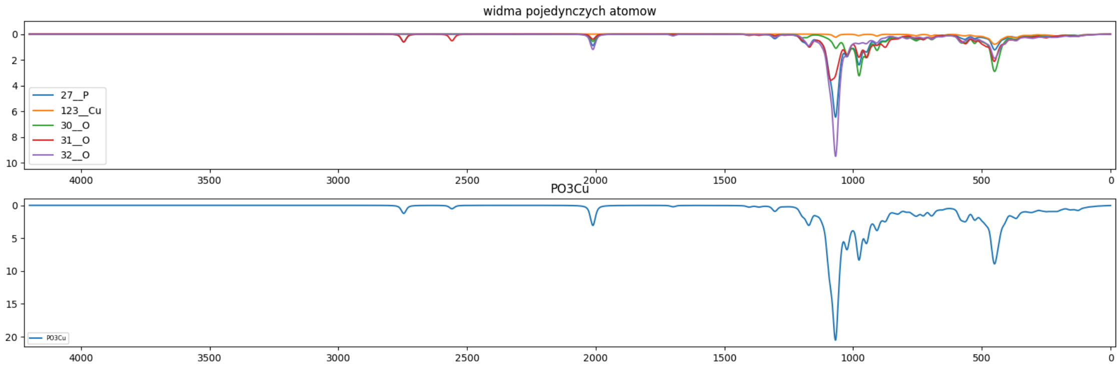

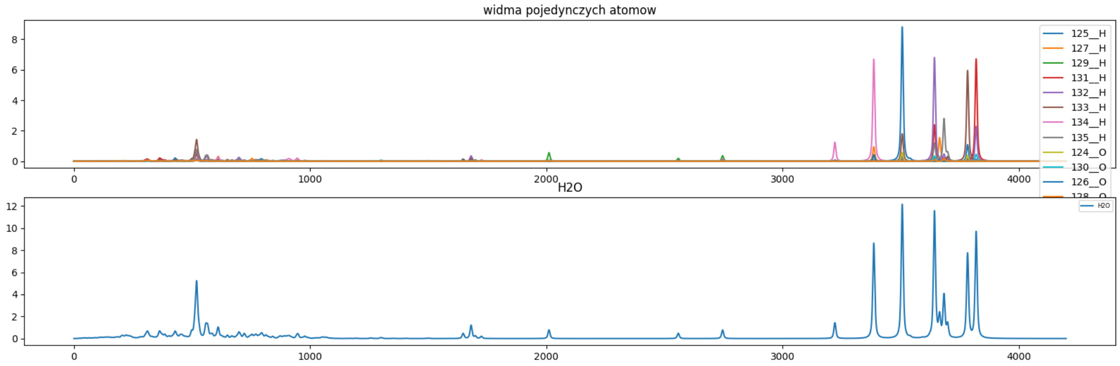

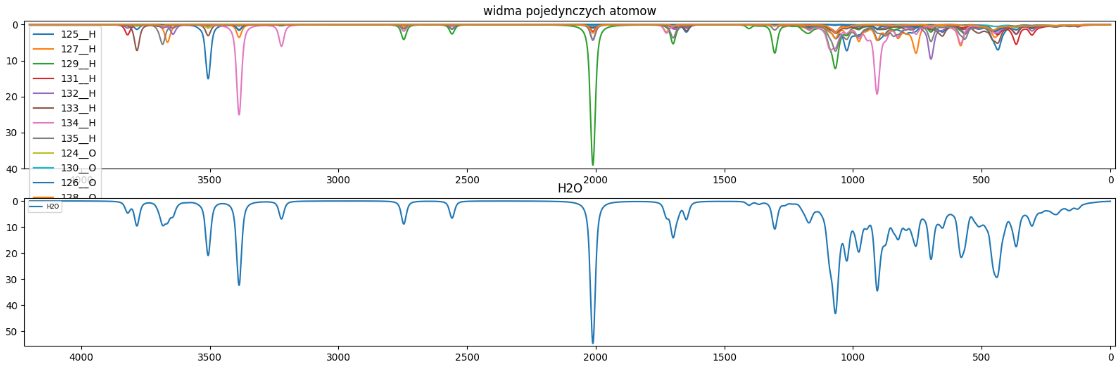

[19]:
```
########### END first anal.
```

[20]:
```
plt.figure(figsize=(20,6))
sumaGrupaRAMAN.index = sumaGrupaRAMANindex
```

```
for i in range(4,len(sumaGrupaRAMAN.index),1):
    print(i)
    plt.plot(sumaGrupaRAMAN.iloc[0],  np.array(sumaGrupaRAMAN.
  ↪iloc[i])+50*i, label= sumaGrupaRAMAN.index[i])
    print(sumaGrupaRAMAN.index[i])
plt.title("RAMAN")
plt.xlim(0,4000)
plt.legend()
plt.savefig(FILENAME+"__________ALL_RAMAN.png")
plt.savefig(FILENAME+"__________ALL_RAMAN.pdf")
pd.DataFrame(sumaGrupaRAMAN.T).
  ↪to_csv(FILENAME+"__________ALL_RAMAN.csv")
pd.DataFrame(sumaGrupaRAMAN.T).
  ↪to_csv(FILENAME+"__________ALL_RAMAN_orgin.csv")
with open(FILENAME+"__________ALL_RAMAN_orgin.csv", 'r+') as␣
  ↪file:
    content = file.read()
    file.seek(0)  # Przenieś kursor na początek pliku
    file.write('LP' + content)  # Dodaj "LP" na początek i␣
  ↪zapisz \n
    file.truncate()  # Usuń ewentualne pozostałości starej␣
  ↪zawartości po nowej
```

```
4
Spectrum All
5
['SiO']
6
['-OH from SiOH']
7
['O-Si(CH3)3']
8
['CH2CH2CH2']
9
['PO3Cu']
10
```

```
['H2O']
11
['ALL-test']
```

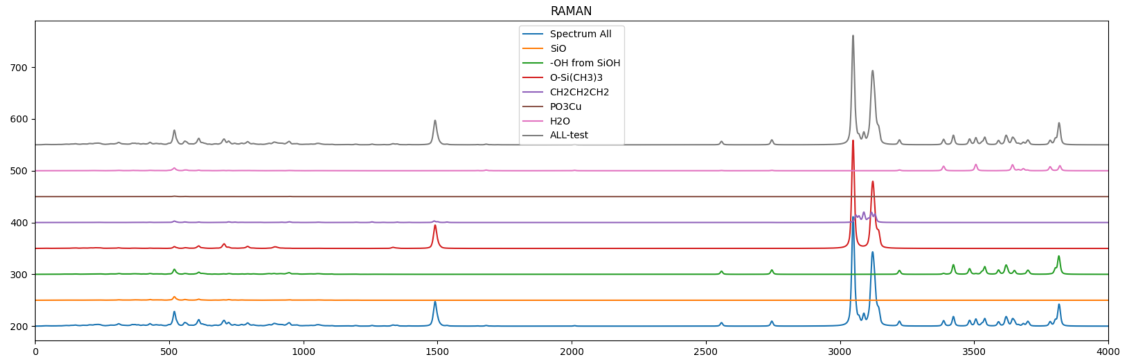

[30]:
```
plt.figure(figsize=(20,6))
plt.stackplot(sumaGrupaRAMAN.iloc[2],  np.array(sumaGrupaRAMAN.
 ↪iloc[5]), np.array(sumaGrupaRAMAN.iloc[6]), np.
 ↪array(sumaGrupaRAMAN.iloc[7]), np.array(sumaGrupaRAMAN.
 ↪iloc[8]),np.array(sumaGrupaRAMAN.iloc[9]),np.
 ↪array(sumaGrupaRAMAN.iloc[10]),
              labels= [sumaGrupaRAMAN.index[5],sumaGrupaRAMAN.
 ↪index[6],sumaGrupaRAMAN.index[7],sumaGrupaRAMAN.
 ↪index[8],sumaGrupaRAMAN.index[9],sumaGrupaRAMAN.index[10]],
             colors=["red", "steelblue","black", "gray",␣
 ↪"orange", "blue" ])
#plt.stackplot(sumaGrupaRAMAN.iloc[0],  np.
 ↪array(sumaGrupaRAMAN.iloc[10]), labels= [sumaGrupaRAMAN.
 ↪index[i]])
#plt.stackplot(sumaGrupaRAMAN.iloc[0],  np.
 ↪array(sumaGrupaRAMAN.iloc['H2O']), labels= [sumaGrupaRAMAN.
 ↪index[i]])
plt.legend()
plt.xlim(0,4000)
plt.ylim(0,38)
plt.savefig(FILENAME+"__________ALL_RAMANcumulative.pdf")
```

```
#plt.plot(sumaGrupaRAMAN.iloc[0],  np.array(sumaGrupaRAMAN.
↪iloc[i]), label= sumaGrupaRAMAN.index[i])
```

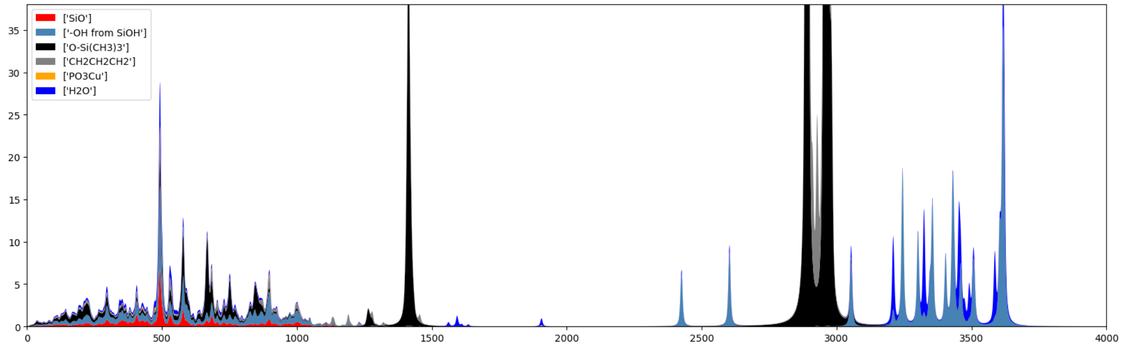

[29]:
```
plt.figure(figsize=(20,6))

i=4
plt.plot(sumaGrupaRAMAN.iloc[2],  np.array(sumaGrupaRAMAN.
↪iloc[i])+50*i, label= sumaGrupaRAMAN.index[i],␣
↪color="green")
i=5
plt.plot(sumaGrupaRAMAN.iloc[2],  np.array(sumaGrupaRAMAN.
↪iloc[i])+50*i, label= sumaGrupaRAMAN.index[i], color="red")
i=6
plt.plot(sumaGrupaRAMAN.iloc[2],  np.array(sumaGrupaRAMAN.
↪iloc[i])+50*i, label= sumaGrupaRAMAN.index[i],␣
↪color="steelblue")
i=7
plt.plot(sumaGrupaRAMAN.iloc[2],  np.array(sumaGrupaRAMAN.
↪iloc[i])+50*i, label= sumaGrupaRAMAN.index[i],␣
↪color="black")
i=8
plt.plot(sumaGrupaRAMAN.iloc[2],  np.array(sumaGrupaRAMAN.
↪iloc[i])+50*i, label= sumaGrupaRAMAN.index[i], color="gray")
i=9
```

```
plt.plot(sumaGrupaRAMAN.iloc[2],  np.array(sumaGrupaRAMAN.
 ↪iloc[i])+50*i, label= sumaGrupaRAMAN.index[i],␣
 ↪color="orange")
i=10
plt.plot(sumaGrupaRAMAN.iloc[2],  np.array(sumaGrupaRAMAN.
 ↪iloc[i])+50*i, label= sumaGrupaRAMAN.index[i], color="blue")


plt.title("RAMAN")
plt.xlim(0,4000)
plt.legend()
#plt.savefig(FILENAME+"__________ALL_RAMAN.png")
plt.savefig(FILENAME+"__________ALL_RAMAN.pdf")
#pd.DataFrame(sumaGrupaRAMAN.T).
 ↪to_csv(FILENAME+"__________ALL_RAMAN.csv")
#pd.DataFrame(sumaGrupaRAMAN.T).
 ↪to_csv(FILENAME+"__________ALL_RAMAN_orgin.csv")
```

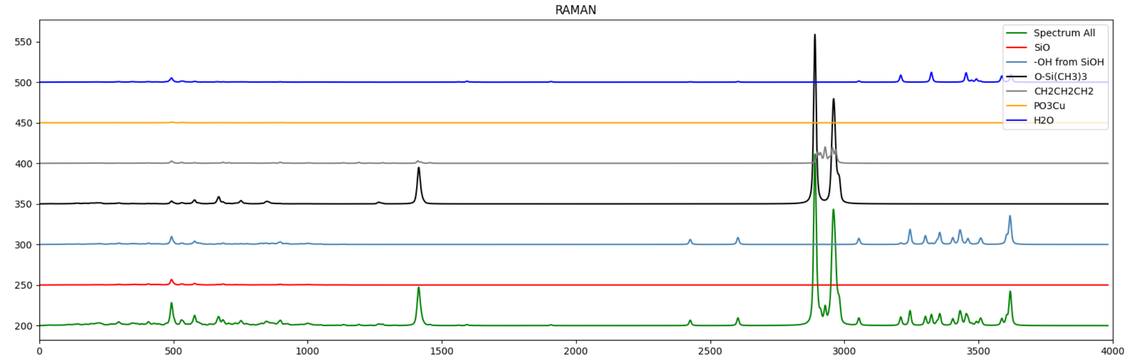

[23]:
```
plt.figure(figsize=(20,6))
sumaGrupaIR.index = sumaGrupaIRindex
minIR = 1000
maxIR = 0
for i in range(4,len(sumaGrupaRAMAN.index),1):
    print(i)
```

```
    plt.plot(sumaGrupaIR.iloc[0],  np.array(sumaGrupaIR.
 ↪iloc[i])+50*i, label= sumaGrupaIR.index[i])
    print(sumaGrupaIR.index[i])
    minIR = min(min(np.array(sumaGrupaIR.iloc[i])+50*i), minIR)
    maxIR = max(max(np.array(sumaGrupaIR.iloc[i])+50*i), maxIR)
    #print(minIR)
    #print(maxIR)
plt.title("IR")
plt.ylim(maxIR, minIR-2)
plt.xlim(4220,-20)
plt.legend()
plt.savefig(FILENAME+"__________ALL_IR.png")
plt.savefig(FILENAME+"__________ALL_IR.pdf")
pd.DataFrame(sumaGrupaIR.T).to_csv(FILENAME+"__________ALL_IR.
 ↪csv")
pd.DataFrame(sumaGrupaIR.T).
 ↪to_csv(FILENAME+"__________ALL_IR_orgin.csv")
with open(FILENAME+"__________ALL_IR_orgin.csv", 'r+') as file:
    content = file.read()
    file.seek(0)  #
    file.write('LP' + content)  #
    file.truncate()  #
```

```
4
Spectrum All
5
['SiO']
6
['-OH from SiOH']
7
['O-Si(CH3)3']
8
['CH2CH2CH2']
9
['PO3Cu']
10
```

```
['H2O']
11
['ALL-test']
```

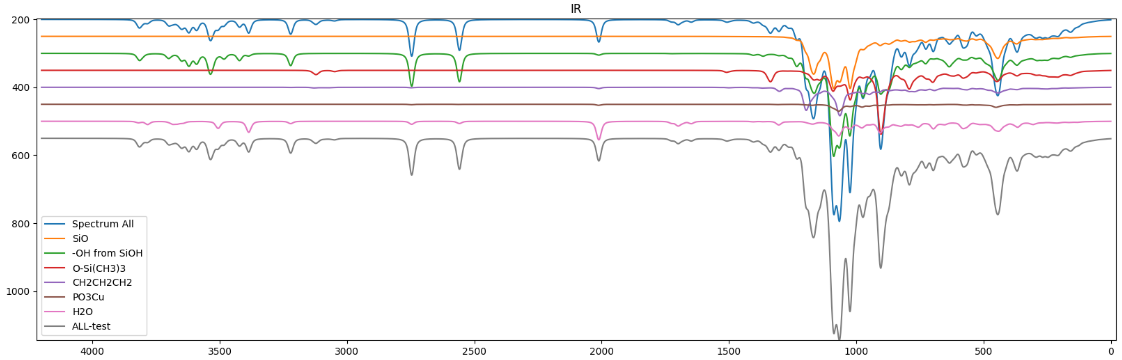

7 Structural database of structures from publication

Below, we present the XYZ coordinates for the models presented in the article. Each model molecule can be reproduced on this basis. B3LYP-D3(BJ)/6-31G(d).

7.1 SBA-15

O	-1.78050900	-2.70645900	1.18547300
O	-4.08978000	-1.74773000	2.06038400
Si	-5.24981800	-1.31805600	0.99371400
Si	-2.47315000	-1.49487000	2.07517200
O	-4.54716100	-1.12865800	-0.49163200
Si	1.14376000	-0.16270000	1.68373500
O	1.01814900	0.81434100	0.39296800
O	0.61848500	-1.64131900	1.23859700
Si	1.46222000	2.10340600	-0.50082100
O	0.34758000	3.26908100	-0.27954800
O	2.94369400	2.62099700	-0.01508300
Si	-0.71971600	3.74340200	0.89064600
O	-1.28775600	2.39263000	1.67613800
Si	-2.62343200	1.46975400	1.39787700
O	-3.71256100	1.69539500	2.58038500
O	-2.11827100	-0.08931900	1.28582700
Si	-5.12656300	-0.47053300	-1.88204700
O	-4.20704900	0.83501000	-2.30404500
Si	-2.92307500	1.55951200	-1.61372700
O	-3.25367700	1.87467400	-0.05869600
Si	-0.40609100	-2.51224900	0.31996500
O	-1.59926700	0.61169800	-1.78555200
O	-0.75575000	-1.86367000	-1.12197700
Si	-1.04964600	-0.82609800	-2.32649200
O	2.70446700	-0.32210000	2.13850100
Si	3.85302200	-1.48713600	1.98462600
Si	4.45292700	2.01895300	-0.15658600
O	3.64547000	-2.30849800	0.59393100
O	4.35407300	0.46133600	-0.69874400

Si	5.55961600	-0.49755400	-1.35405200
Si	3.38463100	-2.34641600	-1.01843600
O	4.79424800	-1.89915600	-1.75767300
O	-2.60696700	3.04754700	-2.25704000
O	-6.65764200	0.00606600	-1.49899900
O	-1.89061400	4.60847700	0.15804100
H	-2.19833500	4.26162100	-0.70154800
O	-5.10313500	-1.52172500	-3.13038200
H	-4.18465700	-1.63289600	-3.44712200
O	-2.20303900	-1.43420900	-3.35933800
H	-1.82894000	-2.03933600	-4.01768700
O	0.33840000	-0.55355400	-3.15937400
H	1.11319500	-0.99615900	-2.72643700
O	-1.91704200	-1.43717100	3.60373100
H	-1.13413500	-0.85593900	3.68779000
O	0.28050400	0.41505500	2.95824900
H	-0.19646600	1.24396500	2.74654300
O	0.01084000	4.59639800	2.07853500
H	0.37131400	5.45536800	1.81940400
O	1.64972200	1.65877900	-2.06146400
H	0.98542100	1.09373000	-2.50746600
O	2.23045900	-1.23389100	-1.46172200
H	2.52645300	-0.31222800	-1.34472600
O	2.86709300	-3.80230100	-1.51637600
H	1.99171000	-4.03651700	-1.14551700
O	5.42776700	2.89045500	-1.14371600
H	5.72839300	2.41342900	-1.93680100
O	5.12877100	2.00355000	1.36438300
H	5.97906500	2.46560100	1.41088900
O	6.78227900	-0.64710700	-0.29481900
H	6.47397600	-0.82505400	0.62226700
O	6.13313700	0.27365200	-2.69277300
H	5.92039100	-0.13912500	-3.54097400
O	5.30146400	-0.68909600	2.04194900
H	5.21008000	0.29092200	1.98346400
O	3.78285100	-2.62984300	3.15622600

H	4.10334800	-2.34893000	4.02443800
O	-5.84488900	0.14350500	1.49335200
H	-6.44797500	0.54383900	0.84372200
O	-6.47068800	-2.40694000	0.96197300
H	-6.25446000	-3.28660300	0.62253700
H	-4.57740200	1.26597500	2.40873500
H	-7.21031600	0.33934300	-2.21939400
H	-2.05444400	3.07502800	-3.05062000
O	0.32869400	-3.95708000	-0.02929200
H	0.55939300	-4.47838600	0.75526200

7.2 SBAH-15

O	1.81391600	-2.51974800	-1.78321700
O	4.18228700	-1.36695400	-1.20969600
Si	4.68036700	-2.05892400	0.20706200
Si	2.85512900	-1.31252200	-2.17532200
O	4.37871500	-1.06275100	1.48383300
H	6.14108600	-2.26932300	0.08660900
H	3.95385700	-3.32270100	0.44301400
H	3.27549800	-1.46824100	-3.57319200
H	-0.89366300	0.25227500	-2.80965600
Si	-1.41454500	-0.38990200	-1.59935900
O	-1.10840300	0.51363600	-0.26787000
O	-0.74998500	-1.86880500	-1.34701800
Si	-1.49085700	1.91106600	0.50384000
O	-0.43033200	3.08735600	0.06013500
H	-1.43874600	1.72659100	1.95286300
O	-2.99019400	2.39188900	0.04208800
Si	0.10768200	3.67512100	-1.38962500
H	-1.02394700	3.86874900	-2.32180300
O	1.13218400	2.58954100	-2.09747200
Si	2.45021500	1.69925100	-1.69977800
O	2.07305100	0.11997400	-1.96049100
Si	4.94323000	0.30294100	2.22172900
O	3.72135000	1.40412200	2.32523500
H	5.36113800	-0.02666900	3.60027000
Si	2.40561300	1.83101000	1.43980000
O	2.86304700	1.95709200	-0.13541700
Si	0.57227200	-2.59701200	-0.70286500
O	1.22608500	0.70463700	1.58002900
O	1.05669200	-1.86023600	0.67437500
Si	0.84090000	-0.85324100	1.96061600
H	-0.57023500	-0.86197700	2.38015600
H	1.73655400	-1.32703700	3.03655700
O	-3.03607100	-0.59948600	-1.75416400
Si	-4.10888200	-1.84729900	-1.57018000
H	-5.46322600	-1.26267400	-1.61820000

H	-3.90572000	-2.84307500	-2.64588000
Si	-4.57578500	2.00806900	-0.18651000
H	-5.37449700	3.18445600	0.22779900
H	-4.82714800	1.65930300	-1.59679000
O	-3.88547200	-2.61965200	-0.13098700
O	-5.00221700	0.71336800	0.74885400
Si	-4.65039600	0.01617800	2.20038300
H	-5.86391300	-0.69878500	2.65721400
H	-4.20952300	1.04346100	3.17021500
Si	-3.22649000	-2.56262100	1.37372800
H	-1.78042400	-2.84701300	1.32363400
H	-3.94133100	-3.55770800	2.20491900
O	-3.40323000	-1.05405800	2.02867400
H	0.25508900	-4.00761300	-0.46208700
H	6.06666400	0.86176000	1.43131600
H	3.58790000	2.08866800	-2.54508100
H	0.82154200	4.93756100	-1.10314000
H	1.92332600	3.12101600	1.93687700

7.3 SBA-Si$(CH_3)_3$

O	-0.41405400	-3.45576900	-0.75376000
O	-2.84955900	-3.52816800	0.28341100
Si	-3.97544200	-2.98819100	-0.78077000
Si	-1.31849400	-3.06776200	0.57984600
O	-3.72102300	-1.36292900	-0.94244300
Si	2.14375400	-1.23347800	1.24741800
O	2.01820000	0.26258700	0.56738000
O	1.78986600	-2.26970900	0.04098300
Si	2.14051400	1.89684000	0.77386500
O	0.92648700	2.42454400	1.71323300
O	3.55701500	2.22348100	1.53221100
Si	-0.34602500	2.08496400	2.68794500
O	-0.73020300	0.48761200	2.46771000
Si	-1.93358200	-0.28209800	1.61458600
O	-3.01869300	-0.93407900	2.60292900
O	-1.19183100	-1.42394300	0.71609800
Si	-4.11464300	-0.34773700	-2.17980500
O	-3.37327400	1.10141700	-1.87841000
Si	-2.23508800	1.49968300	-0.78168200
O	-2.61042000	0.86161300	0.66690900
Si	0.88868100	-2.61970500	-1.27352700
O	-0.77535900	0.93073900	-1.27184200
O	0.40524000	-1.32014100	-2.11424600
Si	0.00244100	0.19757400	-2.49685700
O	3.69187700	-1.50174800	1.68772300
Si	5.00405300	-2.22200200	1.00343000
Si	5.12270700	1.93037800	1.17590800
O	4.89664000	-2.14500000	-0.61922100
O	5.18583500	0.90416000	-0.11114500
Si	6.47237600	0.63809300	-1.14017500
Si	4.60954100	-1.36315400	-2.02440900
O	5.88914100	-0.36146600	-2.31055600
O	-2.13353200	3.10092500	-0.51962700
Si	-1.62011800	4.45292500	-1.41660300
C	-0.28541600	5.26509500	-0.38823700

H	0.03468600	6.21676900	-0.82881300
H	-0.63919400	5.46615400	0.62948400
H	0.58982700	4.61066800	-0.31805800
C	-3.14163100	5.52676400	-1.62871800
H	-2.91453200	6.44040300	-2.19068900
H	-3.92691400	4.98439500	-2.16801700
H	-3.54906600	5.82344700	-0.65556600
C	-0.96688700	3.85204200	-3.07326300
H	-0.00394300	3.34305000	-2.96555400
H	-1.66792700	3.15844400	-3.55269900
H	-0.81927700	4.69775600	-3.75549700
Si	-3.80728100	-0.46204200	4.02548700
C	-4.39327600	1.31005400	3.81653800
H	-4.85365100	1.67571400	4.74267400
H	-3.56688800	1.98465000	3.56539200
H	-5.13835300	1.38594700	3.01796100
C	-2.55370900	-0.58611600	5.41645800
H	-2.14090300	-1.59935800	5.48238200
H	-1.72161100	0.10861100	5.25309400
H	-3.00757900	-0.34470300	6.38521500
C	-5.23281300	-1.65970900	4.21632600
H	-5.89694900	-1.60133500	3.34689800
H	-4.86831800	-2.69042300	4.28742100
H	-5.82402600	-1.44407200	5.11421000
O	-5.71964800	-0.14412500	-2.25282400
Si	-6.75904800	0.57534500	-1.12546400
C	-6.24590900	0.04693100	0.60134100
H	-5.23535400	0.39403700	0.84030400
H	-6.93282300	0.46104800	1.34997300
H	-6.24355500	-1.04394500	0.69719500
C	-6.60611200	2.43828500	-1.32253800
H	-5.57698600	2.76078500	-1.12745100
H	-6.86753700	2.75083900	-2.33992800
H	-7.26510800	2.96954600	-0.62543500
C	-8.47921800	-0.03470000	-1.55355800
H	-8.74156500	0.21830400	-2.58685300

H	-8.54572600	-1.12340800	-1.44723400
H	-9.23293700	0.41238300	-0.89448800
O	-1.56892100	3.11531000	2.36057800
H	-1.84527800	3.19553400	1.42616700
O	-3.54729600	-0.99657300	-3.58124900
H	-2.66960100	-0.63887800	-3.83543300
O	-1.01684900	0.18057100	-3.81133700
H	-0.58121000	0.36750200	-4.65554700
O	1.34447000	1.07579700	-2.86230800
H	2.17368300	0.53904000	-2.76460700
O	-0.75646600	-3.75828600	1.94736700
H	-0.05821300	-3.22488600	2.37209500
O	1.19389600	-1.45222600	2.55791400
H	0.52274500	-0.75050800	2.70781700
O	0.04296700	2.17466600	4.27620600
H	0.23428600	3.05872100	4.61826000
O	2.15585900	2.61231900	-0.68703300
H	1.72967900	2.17037200	-1.45248000
O	3.23688100	-0.43896200	-1.87783500
H	3.07696500	-0.05692900	-0.99499400
O	4.37239100	-2.36798200	-3.27819500
H	3.51821200	-2.83887100	-3.19808500
O	5.99275200	3.28430300	0.87144100
H	6.35396500	3.33301800	-0.03207000
O	5.79266600	1.15862200	2.49159200
H	6.56973500	1.61343400	2.84904500
O	7.73776500	0.06056800	-0.29764400
H	7.47708600	-0.61354600	0.36849000
O	6.92075200	2.09276600	-1.77883700
H	6.65392700	2.24331000	-2.69625000
O	6.32320300	-1.41837100	1.59232500
H	6.10190400	-0.58175100	2.06522000
O	5.11516000	-3.81942800	1.34614700
H	5.43590000	-4.02762100	2.23445200
O	-5.43588300	-3.33776100	-0.11162200
H	-6.09272300	-3.63318500	-0.75720500

O	-3.88929600	-3.66772600	-2.27452900
H	-3.61413000	-3.04548000	-2.97350700
O	1.80852500	-3.51252300	-2.32220100
H	1.99601100	-4.41243500	-2.01441500

7.4 SBA-PO(OH)$_2$

O	2.01212300	1.48461800	1.78086500
O	4.04228800	-0.06791500	2.41482100
Si	5.00805000	0.14098500	1.10656400
Si	2.41161600	0.03872700	2.46791000
O	4.31256900	-0.58489700	-0.21201500
Si	-1.48022400	-0.22639600	1.83005800
O	-1.55447200	-0.99643700	0.39836700
O	-0.60850900	1.13531200	1.65845700
Si	-2.09943300	-1.94457400	-0.80155100
O	-1.14441500	-3.26601500	-0.89856600
O	-3.63896500	-2.42369000	-0.48975200
Si	-0.54229200	-4.25035300	0.28998000
O	0.29590400	-3.32661600	1.38236300
Si	1.83396100	-2.72367400	1.31061700
O	2.77264100	-3.32600300	2.50369400
O	1.73217100	-1.11636500	1.50195500
Si	4.93044800	-1.11339600	-1.63729200
O	3.85218700	-2.21249600	-2.23024900
Si	2.35159500	-2.56160100	-1.68966600
O	2.44581800	-3.09933300	-0.15630900
Si	0.67329500	1.80579400	0.87477100
C	0.31908900	3.62299700	0.66489000
C	0.65377000	4.25796800	-0.70622400
C	1.92347600	5.12406700	-0.72932400
H	-0.17777300	4.89775800	-1.01312300
H	0.73079100	3.49200600	-1.48430000
P	3.46070300	4.23814400	-0.42132400
H	2.04065700	5.60239300	-1.70803000
H	1.86611200	5.92452800	0.01731300
O	4.54727700	5.39079000	-0.75325300
O	3.68790800	2.97544000	-1.22263500
O	3.52330700	3.97062400	1.15186400
H	3.94499200	3.09456500	1.35200600
H	5.46277500	5.07876900	-0.64944300
O	1.36355700	-1.27010300	-1.83562400

O	0.87604100	1.08926600	-0.57910200
Si	1.21477700	0.35639900	-1.97459200
O	-2.98428800	0.23021400	2.29026900
Si	-3.93330000	1.54820000	2.07572000
Si	-5.05316600	-1.61390200	-0.39856200
O	-3.53363100	2.35358800	0.71455400
O	-4.73841200	-0.03854400	-0.73884000
Si	-5.76345700	1.13698200	-1.31737900
Si	-3.29970200	2.60785800	-0.88060900
O	-4.75619600	2.39530200	-1.64027900
O	1.68560500	-3.82558600	-2.52526700
O	6.38931600	-1.78749000	-1.24180900
O	0.38394300	-5.37700000	-0.43864500
H	0.89214100	-5.04483700	-1.20680800
O	5.20360500	0.05916000	-2.73881600
H	4.35807900	0.44847100	-3.04580400
O	2.59108000	0.98635100	-2.61087300
H	2.92047600	1.79091400	-2.13108400
O	-0.09957400	0.52384500	-2.96889800
H	-0.69461900	1.24882400	-2.68060100
O	1.85189000	-0.12898100	3.98855300
H	0.93574700	-0.46693400	4.00124500
O	-0.84480600	-1.21054600	2.98394700
H	-0.57682800	-2.08192900	2.62947800
O	-1.73916200	-4.90087200	1.19683700
H	-2.44822100	-5.33646300	0.70414900
O	-2.13757200	-1.09753100	-2.20575300
H	-1.28987900	-0.77061600	-2.59247300
O	-2.17023100	1.62085400	-1.54905800
H	-2.43777900	0.68064700	-1.65279100
O	-2.72557000	4.12742600	-1.10714500
H	-3.11810300	4.79240000	-0.52419200
O	-6.20478600	-2.21153700	-1.39903700
H	-6.47566900	-1.59868500	-2.10599400
O	-5.61706700	-1.69763000	1.16472000
H	-6.47912100	-2.13099900	1.24906800

O	-6.94675300	1.43820600	-0.24604100
H	-6.62013000	1.43174800	0.68234800
O	-6.47618600	0.57328400	-2.69578500
H	-6.07920200	0.88094000	-3.52262400
O	-5.48457200	0.96722600	2.04294600
H	-5.52338100	-0.00777500	1.90809900
O	-3.78772700	2.67509200	3.25698800
H	-4.11521800	2.40315500	4.12562000
O	6.47151600	-0.51675400	1.40445800
H	6.79391100	-1.06492300	0.66565900
O	5.07919900	1.76075200	0.75897100
H	4.76454200	1.95660600	-0.15389300
H	-0.76024000	3.70574200	0.83416500
H	0.79895600	4.17318200	1.48184300
H	2.98004700	-4.27004600	2.47159600
H	7.02294100	-1.87174000	-1.96843200
H	1.04870900	-3.57479500	-3.20974400

7.5 SBA-PO(OH)$_2$+3(Si(CH$_3$)$_3$)

O	-0.65128100	2.62722600	-1.78059400
O	-3.07515100	1.78802900	-2.39322300
Si	-3.89722100	2.25613800	-1.05456900
Si	-1.49032500	1.38557300	-2.47131700
O	-3.45798300	1.32003100	0.23106300
Si	2.11568000	-0.09525200	-1.93599300
O	1.95733300	-0.90561300	-0.53411800
O	1.72055900	1.46419700	-1.70691900
Si	2.19206200	-2.06459000	0.58034900
O	0.91596400	-3.07587400	0.57647300
O	3.53802800	-2.93609200	0.22924600
Si	-0.06366700	-3.71625700	-0.58403600
O	-0.53972000	-2.49362600	-1.59358200
Si	-1.80736700	-1.43344100	-1.45649300
O	-2.87159800	-1.66816400	-2.64027800
O	-1.18346300	0.06973400	-1.52337100
Si	-4.24761400	0.90912200	1.62476700
O	-3.53886100	-0.49202600	2.14337200
Si	-2.22612700	-1.25848200	1.54522800
O	-2.50502000	-1.68773800	-0.00276400
Si	0.72900700	2.49125200	-0.88711900
C	1.64584400	4.09775300	-0.65702600
C	1.54740400	4.77492900	0.73118500
C	0.60608400	5.98812400	0.80215800
H	2.54008800	5.12302900	1.02809300
H	1.25471400	4.05064100	1.49755300
P	-1.13551400	5.62478000	0.52547600
H	0.66426700	6.45630000	1.79089400
H	0.88956900	6.74938100	0.06609000
O	-1.81052000	7.04264100	0.91922000
O	-1.72526400	4.46981700	1.30333700
O	-1.30462500	5.43870100	-1.05210600
H	-1.98884800	4.75204700	-1.26341200
H	-2.78243000	7.00552100	0.90266600
O	-0.90064100	-0.31098900	1.64281300

O	0.34218800	1.85392200	0.56464200
Si	-0.23951000	1.15773600	1.90156400
O	3.68537200	-0.11513800	-2.40906700
Si	4.98546400	0.84605400	-2.13954800
Si	5.13228900	-2.60573000	0.18407400
O	4.84074900	1.65698900	-0.73196600
O	5.31942000	-1.03154000	0.61362300
Si	6.66733100	-0.24809200	1.19293000
Si	4.74065600	1.89697200	0.87919900
O	6.09934600	1.24762400	1.56992200
O	-1.95881600	-2.66011300	2.33532900
Si	-1.42135600	-3.00350000	3.91373900
C	0.20780800	-3.91211500	3.74722400
H	0.53946600	-4.30384400	4.71634500
H	0.11777200	-4.75457900	3.05260500
H	0.98855100	-3.25040500	3.36027600
C	-2.75621900	-4.08072400	4.67106800
H	-2.49886800	-4.37980500	5.69403600
H	-3.71191000	-3.54518000	4.70648500
H	-2.90523900	-4.99307000	4.08215000
C	-1.22677500	-1.38453900	4.84521600
H	-0.39775900	-0.78581500	4.45407500
H	-2.13903200	-0.77919600	4.78721300
H	-1.02550700	-1.57681300	5.90601900
Si	-3.37577500	-2.98733500	-3.56894400
C	-3.90940900	-4.35559300	-2.39855800
H	-4.18141800	-5.26269600	-2.95194800
H	-3.10999600	-4.61823800	-1.69604100
H	-4.77925800	-4.04681300	-1.80896600
C	-1.91314900	-3.53048500	-4.61403000
H	-1.52807400	-2.69388000	-5.20886700
H	-1.09774700	-3.89961200	-3.98183900
H	-2.19222700	-4.33357100	-5.30663600
C	-4.79818500	-2.34819000	-4.60542400
H	-5.60830100	-1.98674000	-3.96240100
H	-4.47395400	-1.51226900	-5.23510700

H	-5.20427900	-3.12967500	-5.25855600
O	-5.82089800	0.67691200	1.26208800
Si	-6.69109700	-0.70440400	0.79904000
C	-5.88889600	-1.42342300	-0.73590700
H	-4.88639000	-1.80573400	-0.52429400
H	-6.48979200	-2.24996200	-1.13531500
H	-5.79121600	-0.66515800	-1.52077600
C	-6.66785800	-1.92631800	2.22297100
H	-5.63966400	-2.22141800	2.45805300
H	-7.10760500	-1.48868600	3.12631200
H	-7.23457400	-2.83199200	1.97573900
C	-8.42034400	-0.07909400	0.42915600
H	-8.85547200	0.42153100	1.30120500
H	-8.41204900	0.63653500	-0.40172400
H	-9.08532000	-0.90378300	0.14626700
O	-1.29303300	-4.47175200	0.18021600
H	-1.65295300	-3.97959800	0.94720300
O	-4.13078900	2.07213600	2.76103400
H	-3.19337600	2.19246500	3.02007700
O	-1.33239100	2.16423100	2.60373400
H	-1.38256500	3.05322600	2.16815200
O	1.04917200	0.81099500	2.88658600
H	1.85450700	1.31733900	2.64659600
O	-1.04215700	1.06655800	-4.00684700
H	-0.25768000	0.48420600	-4.03437800
O	1.19440100	-0.78726200	-3.11172300
H	0.66835500	-1.54197000	-2.77697900
O	0.72490900	-4.73854700	-1.59299700
H	1.09552600	-5.53241300	-1.18361000
O	2.44399800	-1.36632400	2.04324900
H	1.74359700	-0.78018100	2.42027400
O	3.39503600	1.24868300	1.56115700
H	3.36145800	0.26836500	1.60031600
O	4.63761100	3.50516400	1.18365000
H	5.21188200	4.05267700	0.63011500
O	6.03056100	-3.58014400	1.14836400

H	6.47840600	-3.11495100	1.87698900
O	5.66304200	-2.78445900	-1.38341900
H	6.37447300	-3.43409000	-1.48297500
O	7.86726000	-0.29013100	0.09829400
H	7.53962700	-0.14521100	-0.81829100
O	7.19638400	-1.04601600	2.53732500
H	6.93757600	-0.65087200	3.38152100
O	6.29316700	-0.17050200	-2.16178300
H	6.04398900	-1.11908600	-2.06742000
O	5.18578000	2.02807900	-3.25780100
H	5.41692300	1.71741900	-4.14424400
O	-5.49760700	2.10019400	-1.37086900
H	-6.01197900	1.90737200	-0.56834800
O	-3.46511500	3.80873600	-0.66381200
H	-3.06935400	3.86299500	0.23752400
H	2.69295500	3.83308200	-0.84316000
H	1.35923100	4.79039400	-1.45608400

7.6 SBA-POO$_2$Cu+3(Si(CH$_3$)$_3$)+4H$_2$O

O	0.81108600	-1.74794000	-2.08954200
O	2.86317800	-0.16764000	-2.69549300
Si	3.77922500	-0.60775200	-1.41403400
Si	1.21325800	-0.28447700	-2.70797400
O	3.20732200	0.19127400	-0.09076800
Si	-2.62733600	-0.17381500	-1.98386800
O	-2.70586000	0.59494300	-0.54794200
O	-1.78984000	-1.55719700	-1.87769200
Si	-3.17744700	1.53630800	0.68311700
O	-2.20757200	2.84481300	0.77176600
O	-4.71625200	2.06261100	0.44537600
Si	-1.61027700	3.90743700	-0.33605900
O	-0.79500400	3.05541700	-1.49587600
Si	0.73750900	2.43026100	-1.43981700
O	1.67196000	3.10941000	-2.56445800
O	0.60626200	0.82872500	-1.65347800
Si	3.86840000	0.64088600	1.34497100
O	2.86389100	1.76831000	1.99616600
Si	1.32665700	2.15828700	1.56363200
O	1.34573300	2.74550500	0.04600700
Si	-0.44836600	-2.17330900	-1.09633800
C	-0.77553000	-3.99490900	-0.82354400
C	-0.62891700	-4.50231100	0.64255600
C	0.61377900	-5.37162600	0.93941000
H	-1.51719900	-5.08153900	0.90585300
H	-0.62644400	-3.65572300	1.33449000
P	2.12230900	-4.64831700	0.22574300
H	0.73612700	-5.49133700	2.02136100
H	0.50923500	-6.37127700	0.50530100
O	3.38972600	-4.82911400	1.15705500
O	1.83474200	-3.10229600	0.15074300
O	2.38156700	-5.26830200	-1.15426600
O	0.36745700	0.85329100	1.70554500
O	-0.38896500	-1.29604100	0.28895500
Si	0.12719900	-0.76394800	1.71483800

O	-4.14901800	-0.57472800	-2.45366600
Si	-5.11873500	-1.86403000	-2.16688400
Si	-6.15065100	1.28830400	0.38484100
O	-4.68591200	-2.64259600	-0.80275700
O	-5.85769500	-0.29378200	0.70211700
Si	-6.89031300	-1.46646700	1.27034500
Si	-4.45354900	-2.97307900	0.77872400
O	-5.90193400	-2.74448500	1.55493600
O	0.75418800	3.37131000	2.48776800
Si	0.13771800	3.41557000	4.07438200
C	-1.70692800	3.70996400	3.94556500
H	-2.14224200	3.90390600	4.93328800
H	-1.92253300	4.57086100	3.30324300
H	-2.21494200	2.84374200	3.51160600
C	1.02063300	4.84742700	4.90344700
H	0.67698400	4.98566200	5.93541400
H	2.10291500	4.67599200	4.92868500
H	0.84217900	5.78378900	4.36241500
C	0.52567300	1.78125000	4.91499600
H	-0.05673600	0.95914800	4.48657600
H	1.58868800	1.52804800	4.82370300
H	0.28974300	1.83582300	5.98462200
Si	1.76359300	4.63931000	-3.27482100
C	1.89100500	5.91460800	-1.90150100
H	1.92896200	6.93115300	-2.31175200
H	1.03242000	5.85864000	-1.22255500
H	2.79656000	5.75876800	-1.30478600
C	0.20436600	4.89478700	-4.28749400
H	0.09284300	4.10300700	-5.03734900
H	-0.68307200	4.87671900	-3.64513500
H	0.22141800	5.85670000	-4.81376700
C	3.30381400	4.60110100	-4.34207600
H	4.19342800	4.41157200	-3.73122400
H	3.23594800	3.80396000	-5.09072400
H	3.45008300	5.55119600	-4.86951600
O	5.34885700	1.26350100	1.04962000

Si	5.79148300	2.87672000	0.72092500
C	4.77416900	3.44095700	-0.75222100
H	3.70310700	3.43756000	-0.52824700
H	5.05091600	4.46017500	-1.04842000
H	4.92386900	2.79360800	-1.62472400
C	5.45955600	3.92255100	2.23935400
H	4.39333900	3.91393000	2.48729000
H	6.01198500	3.54406800	3.10682600
H	5.76450300	4.96265500	2.07336800
C	7.61723900	2.78903800	0.30140000
H	8.19312200	2.37125700	1.13464200
H	7.79638500	2.16092800	-0.57957500
H	8.01761500	3.78567800	0.08119500
O	-0.67641900	4.97661700	0.47664000
H	-0.11269400	4.58080700	1.17144400
O	3.98453000	-0.64409400	2.37010700
H	3.05801700	-0.99642300	2.50339600
O	1.50145000	-1.56208500	2.14566100
H	1.70123000	-2.25667200	1.41292700
O	-1.11048400	-0.93857400	2.80773300
H	-1.71832900	-1.66887000	2.56364200
O	0.64707500	0.00324000	-4.20914800
H	-0.29970200	0.24892500	-4.17603200
O	-1.97781000	0.85263600	-3.10438900
H	-1.72222500	1.71341700	-2.71904800
O	-2.78939300	4.66335400	-1.18514000
H	-3.48389900	5.08254200	-0.65868500
O	-3.17041800	0.68778900	2.08863200
H	-2.31252100	0.34848200	2.44079000
O	-3.30058800	-2.04461600	1.49071500
H	-3.53126900	-1.09477500	1.59009800
O	-3.95430200	-4.52545200	0.93556100
H	-4.21512300	-5.09839000	0.20103000
O	-7.26422700	1.89477000	1.42427200
H	-7.52624800	1.27168900	2.12580900
O	-6.75373900	1.41300400	-1.16138800

H	-7.63551000	1.81148300	-1.20309300
O	-8.09551200	-1.72074800	0.20974800
H	-7.77740800	-1.70742700	-0.72178300
O	-7.57502100	-0.91694100	2.67040000
H	-7.17682900	-1.26147000	3.48197900
O	-6.65557700	-1.24494600	-2.09030500
H	-6.66880900	-0.27188200	-1.94214900
O	-5.05769600	-3.02297600	-3.32551800
H	-5.39125500	-2.75176200	-4.19197000
O	5.39992200	-0.25269800	-1.57260200
H	5.68223100	0.40661200	-0.91271500
O	3.75571400	-2.21981400	-1.18919300
H	2.86296200	-2.55251700	-0.79314500
H	-1.80809100	-4.15025800	-1.15751000
H	-0.13932000	-4.57466200	-1.50199500
Cu	4.87890000	-3.83220000	0.42217100
O	6.31786800	-2.48765900	-0.04827600
H	6.13404600	-1.82876200	-0.75615100
O	4.99982100	-3.15447400	2.27198600
H	4.20336300	-3.60253600	2.62014800
O	4.76848600	-4.66970700	-1.39305900
H	3.76833600	-5.07094800	-1.39580800
O	6.85905600	-5.14861200	0.16958100
H	7.02000600	-5.90677900	0.75107300
H	6.39103900	-5.49274200	-0.61595800
H	7.14572700	-2.95822500	-0.23811700
H	4.82969500	-2.17864300	2.33462900
H	4.65509700	-3.87828700	-1.95417200

7.7 SBA-POO$_2$Cu+3(Si(CH$_3$)$_3$)

O	0.69182800	2.53537700	1.42318500
O	3.14291800	1.74716200	2.02831000
Si	4.10494000	1.68671300	0.72029900
Si	1.54087100	1.38626300	2.20255700
O	3.48836600	0.69076700	-0.41557600
Si	-2.26765400	0.25407800	1.90937100
O	-2.21166300	-0.76566700	0.64147100
O	-1.81774900	1.74604100	1.45149500
Si	-2.55504600	-2.03148900	-0.31547900
O	-1.36991800	-3.14019600	-0.17749900
O	-3.96750900	-2.73324600	0.14236800
Si	-0.43759300	-3.72342600	1.04774700
O	0.21458500	-2.44368400	1.87331700
Si	1.60210200	-1.59271800	1.55652500
O	2.69754800	-1.81880200	2.71479200
O	1.19248000	-0.02539200	1.43627500
Si	4.11935800	0.07705300	-1.82623100
O	3.22016100	-1.26195800	-2.17235700
Si	1.86515100	-1.83020400	-1.45347400
O	2.18016500	-2.11509100	0.12149600
Si	-0.67876800	2.50023600	0.52117300
C	-1.35750800	4.18450900	0.09128100
C	-1.05951600	4.77291600	-1.31014000
C	0.09801100	5.79078000	-1.35925600
H	-1.95636900	5.28121100	-1.67841600
H	-0.85419200	3.97210200	-2.02702400
P	1.66027200	5.07443500	-0.80592800
H	0.24645700	6.15157600	-2.38353200
H	-0.10584800	6.65945000	-0.72435000
O	2.95817700	5.97936400	-1.01344700
O	1.86953900	3.68592900	-1.42693400
O	1.72200400	5.05521300	0.79164400
O	0.63827300	-0.76899400	-1.61479500
O	-0.43487800	1.58540400	-0.80752000
Si	0.11311200	0.71741500	-2.05247400

O	-3.81725300	0.38547500	2.43435900
Si	-5.03416200	1.40781900	2.02138200
Si	-5.52708800	-2.26342800	0.11766500
O	-4.82104300	2.00057900	0.51868900
O	-5.57302900	-0.74316900	-0.50126900
Si	-6.85049700	0.09562100	-1.15953800
Si	-4.72063600	2.05732500	-1.10945800
O	-6.15675600	1.48085300	-1.70699400
O	1.44092500	-3.28024400	-2.06357900
Si	0.80670500	-3.76505500	-3.56633500
C	-0.89778600	-4.46751600	-3.23727700
H	-1.30299900	-4.95137400	-4.13412500
H	-0.86572800	-5.21216200	-2.43419200
H	-1.59376600	-3.68172000	-2.92875800
C	1.99393500	-5.06757900	-4.20781200
H	1.66878700	-5.46563900	-5.17629800
H	2.99787000	-4.64662400	-4.33626600
H	2.07020600	-5.90794900	-3.50824000
C	0.74425700	-2.26891800	-4.69889900
H	0.00632200	-1.53251600	-4.36423900
H	1.71937600	-1.77044300	-4.75293900
H	0.47210400	-2.57169700	-5.71727500
Si	3.07819600	-3.06141300	3.79570900
C	3.38661400	-4.62589200	2.80469000
H	3.57028000	-5.48075900	3.46692000
H	2.52968900	-4.87247100	2.16726400
H	4.26005600	-4.50955800	2.15406800
C	1.61363100	-3.27323700	4.95112700
H	1.36802000	-2.32579100	5.44503300
H	0.72698700	-3.60661200	4.40024900
H	1.82364300	-4.01413200	5.73183500
C	4.61708600	-2.48024200	4.69198700
H	5.43489600	-2.31401200	3.98187500
H	4.42909200	-1.53498100	5.21314100
H	4.95388300	-3.21583800	5.43204100
O	5.67196000	-0.31319700	-1.52243400

Si	6.39160200	-1.75469200	-0.99448800
C	5.60622900	-2.24774100	0.63680900
H	4.55338900	-2.51660100	0.51191400
H	6.12714200	-3.11052200	1.07037900
H	5.64816500	-1.42803300	1.36285000
C	6.13319400	-3.07172700	-2.30518800
H	5.06432000	-3.25577300	-2.45729300
H	6.56205600	-2.76321200	-3.26529000
H	6.60406400	-4.01796300	-2.01302400
C	8.20559500	-1.33089900	-0.76718800
H	8.64157400	-0.94854700	-1.69672900
H	8.34406500	-0.56745800	0.00820000
H	8.78026100	-2.21369100	-0.46264300
O	0.67300000	-4.71588600	0.37495100
H	1.05407500	-4.38058600	-0.46219300
O	4.04969800	1.16272000	-3.03578300
H	3.11462800	1.35338500	-3.26763000
O	1.29581900	1.55231900	-2.82579900
H	1.45324000	2.43844600	-2.39091100
O	-1.18910600	0.36727300	-3.02057900
H	-1.93983700	0.97865200	-2.86245100
O	1.19540800	1.26923200	3.79346700
H	0.32401700	0.84756700	3.92674300
O	-1.30860200	-0.32057800	3.12079300
H	-0.87654200	-1.16688700	2.88561800
O	-1.30784500	-4.49684600	2.20055100
H	-1.81960400	-5.26067700	1.90063600
O	-2.76324000	-1.52087100	-1.85985700
H	-2.02163500	-1.05808900	-2.32080100
O	-3.47260000	1.19936100	-1.74107400
H	-3.52822600	0.22411200	-1.64889700
O	-4.45111000	3.60319200	-1.58492200
H	-4.92505200	4.26872900	-1.06706800
O	-6.50540800	-3.26241400	-0.73821200
H	-6.91000000	-2.84506900	-1.51920700
O	-6.07520500	-2.21475900	1.68901900

H	-6.87232300	-2.74477600	1.83705000
O	-8.03159800	0.28426500	-0.05901100
H	-7.67725300	0.50469900	0.83232100
O	-7.46976500	-0.79711200	-2.40239200
H	-7.20440500	-0.51063800	-3.28741300
O	-6.42403100	0.51787900	2.16900800
H	-6.26263200	-0.45380500	2.18396200
O	-5.13942900	2.74103000	2.97032400
H	-5.40568600	2.56967800	3.88434600
O	5.62270900	1.30478100	1.19442100
H	6.18592300	1.03843800	0.44885000
O	4.15773700	3.19968100	-0.03906100
H	3.45925700	3.19878200	-0.76847000
H	-2.44126100	4.05131200	0.18537100
H	-1.06591500	4.88643500	0.88165700
Cu	3.57917600	5.02555400	0.53087700

References

[1] Sara El Houbbadi, Magdalena Laskowska, Alain Walcarius, Marek Doskocz, Alexey Maximenko, Zbigniew Olejniczak, and Łukasz Laskowski. Revealing the molecular structure of copper phosphonate groups anchored inside sba-15 silica channels: Theoretical and experimental study. *Applied Surface Science*, 669:160425, 2024.

[2] M. J. Frisch, G. W. Trucks, H. B. Schlegel, G. E. Scuseria, M. A. Robb, J. R. Cheeseman, G. Scalmani, V. Barone, G. A. Petersson, H. Nakatsuji, X. Li, M. Caricato, A. V. Marenich, J. Bloino, B. G. Janesko, R. Gomperts, B. Mennucci, H. P. Hratchian, J. V. Ortiz, A. F. Izmaylov, J. L. Sonnenberg, D. Williams-Young, F. Ding, F. Lipparini, F. Egidi, J. Goings, B. Peng, A. Petrone, T. Henderson, D. Ranasinghe, V. G. Zakrzewski, J. Gao, N. Rega, G. Zheng, W. Liang, M. Hada, M. Ehara, K. Toyota, R. Fukuda, J. Hasegawa, M. Ishida, T. Nakajima, Y. Honda, O. Kitao, H. Nakai, T. Vreven, K. Throssell, J. A. Montgomery, Jr., J. E. Peralta, F. Ogliaro, M. J. Bearpark, J. J. Heyd, E. N. Brothers, K. N. Kudin, V. N. Staroverov, T. A. Keith, R. Kobayashi, J. Normand, K. Raghavachari, A. P. Rendell, J. C. Burant, S. S. Iyengar, J. Tomasi, M. Cossi, J. M. Millam, M. Klene, C. Adamo, R. Cammi, J. W. Ochterski, R. L. Martin, K. Morokuma, O. Farkas, J. B. Foresman, and D. J. Fox. Gaussian~16 Revision C.01, 2016. Gaussian Inc. Wallingford CT.

[3] Małgorzata A. Małecka, Piotr Kraszkiewicz, Krzysztof Matus, Magdalena Laskowska, and Marek Doskocz. A novel method for the synthesis of bubble-like mesoporous silica - the influence of dopants. *Materials Chemistry and Physics*, 333:130405, March 2025.

[4] Łukasz Laskowski, Magdalena Laskowska, Alain Walcarius, Marek Doskocz, Neus Vila, Agnieszka Karczmarska, Piotr Pawlik, Jerzy Goraus, Katarzyna Balin, and Mateusz Dulski. Synthesis and characterization of sba-15 silica containing cyclam inside pores for capturing iron chloride: Analysis of interactions between ferrous chloride and cyclam in a system with strongly dispersed functional groups on the sio2 surface. *Microporous and Mesoporous Materials*, page 113243, 2024.
